# APEX2 Proximity Proteomics Resolves Flagellum Subdomains and Identifies Flagellum Tip-Specific Proteins in Trypanosoma brucei

**DOI:** 10.1128/mSphere.01090-20

**Published:** 2021-02-10

**Authors:** Daniel E. Vélez-Ramírez, Michelle M. Shimogawa, Sunayan S. Ray, Andrew Lopez, Shima Rayatpisheh, Gerasimos Langousis, Marcus Gallagher-Jones, Samuel Dean, James A. Wohlschlegel, Kent L. Hill

**Affiliations:** a Department of Microbiology, Immunology, and Molecular Genetics, University of California Los Angeles, Los Angeles, California, USA; b Posgrado en Ciencias Biológicas, Universidad Nacional Autónoma de México, Coyoacán, Ciudad de México, México; c Department of Biological Chemistry, University of California Los Angeles, Los Angeles, California, USA; d Department of Chemistry and Biochemistry, UCLA-DOE Institute for Genomics and Proteomics, Los Angeles, California, USA; e Warwick Medical School, University of Warwick, Coventry, United Kingdom; f Molecular Biology Institute, University of California Los Angeles, Los Angeles, California, USA; g California NanoSystems Institute, University of California Los Angeles, Los Angeles, California, USA; University of Texas Southwestern

**Keywords:** *Trypanosoma*, cell signaling, flagella

## Abstract

Trypanosoma brucei is the protozoan parasite responsible for sleeping sickness, a lethal vector-borne disease. T. brucei has a single flagellum (cilium) that plays critical roles in transmission and pathogenesis. An emerging concept is that the flagellum is organized into subdomains, each having specialized composition and function. The overall flagellum proteome has been well studied, but a critical knowledge gap is the protein composition of individual subdomains. We have tested whether APEX-based proximity proteomics could be used to examine the protein composition of T. brucei flagellum subdomains. As APEX-based labeling has not previously been described in T. brucei, we first fused APEX2 to the DRC1 subunit of the nexin-dynein regulatory complex, a well-characterized axonemal complex. We found that DRC1-APEX2 directs flagellum-specific biotinylation, and purification of biotinylated proteins yields a DRC1 “proximity proteome” having good overlap with published proteomes obtained from purified axonemes. Having validated the use of APEX2 in T. brucei, we next attempted to distinguish flagellar subdomains by fusing APEX2 to a flagellar membrane protein that is restricted to the flagellum tip, AC1, and another one that is excluded from the tip, FS179. Fluorescence microscopy demonstrated subdomain-specific biotinylation, and principal-component analysis showed distinct profiles between AC1-APEX2 and FS179-APEX2. Comparing these two profiles allowed us to identify an AC1 proximity proteome that is enriched for tip proteins, including proteins involved in signaling. Our results demonstrate that APEX2-based proximity proteomics is effective in T. brucei and can be used to resolve the proteome composition of flagellum subdomains that cannot themselves be readily purified.

**IMPORTANCE** Sleeping sickness is a neglected tropical disease caused by the protozoan parasite Trypanosoma brucei. The disease disrupts the sleep-wake cycle, leading to coma and death if left untreated. T. brucei motility, transmission, and virulence depend on its flagellum (cilium), which consists of several different specialized subdomains. Given the essential and multifunctional role of the T. brucei flagellum, there is need for approaches that enable proteomic analysis of individual subdomains. Our work establishes that APEX2 proximity labeling can, indeed, be implemented in the biochemical environment of T. brucei and has allowed identification of proximity proteomes for different flagellar subdomains that cannot be purified. This capacity opens the possibility to study the composition and function of other compartments. We expect this approach may be extended to other eukaryotic pathogens and will enhance the utility of T. brucei as a model organism to study ciliopathies, heritable human diseases in which cilium function is impaired.

## Abstract (precis)

Sleeping sickness is a neglected tropical disease caused by the protozoan parasite Trypanosoma brucei. The disease disrupts the sleep-wake cycle, leading to coma and death if left untreated. T. brucei motility, transmission, and virulence depend on its flagellum (cilium), which consists of several different specialized subdomains.

## INTRODUCTION

Trypanosoma brucei is a flagellated parasite that is transmitted between mammalian hosts by a hematophagous vector, the tsetse fly, and is of medical relevance as the causative agent of sleeping sickness in humans (1). T. brucei also presents a substantial economic burden in regions of endemicity due to infection of livestock, causing an estimated loss of more than $1 billion/year (2). As such, T. brucei is considered both cause and consequence of poverty in some of the poorest regions in the world. T. brucei also provides an excellent model system for understanding the cell and molecular biology of related flagellates T. cruzi and *Leishmania* spp., which together present a tremendous health burden across the globe.

T. brucei has a single flagellum (cilium), which is essential for parasite viability, infection, and transmission (3–5). The flagellum drives parasite motility, which is necessary for infection of the mammalian host (4) and for transmission by the tsetse fly (5). In addition to its canonical function in motility, the flagellum plays important roles in cell division and morphogenesis (6–8) and mediates direct interaction with host tissues (9). Moreover, recent work has demonstrated that the trypanosome flagellum is the site of signaling pathways that control the parasite’s response to external signals and are required for transmission and virulence (4, 10–15). Human cilia are likewise used for motility and sensory functions, and defects in these functions underlie a broad spectrum of inherited diseases termed ciliopathies (16). The ease of forward and reverse genetic manipulation of T. brucei makes this parasite an ideal model organism to study ciliopathies (17).

**FIG 1:**
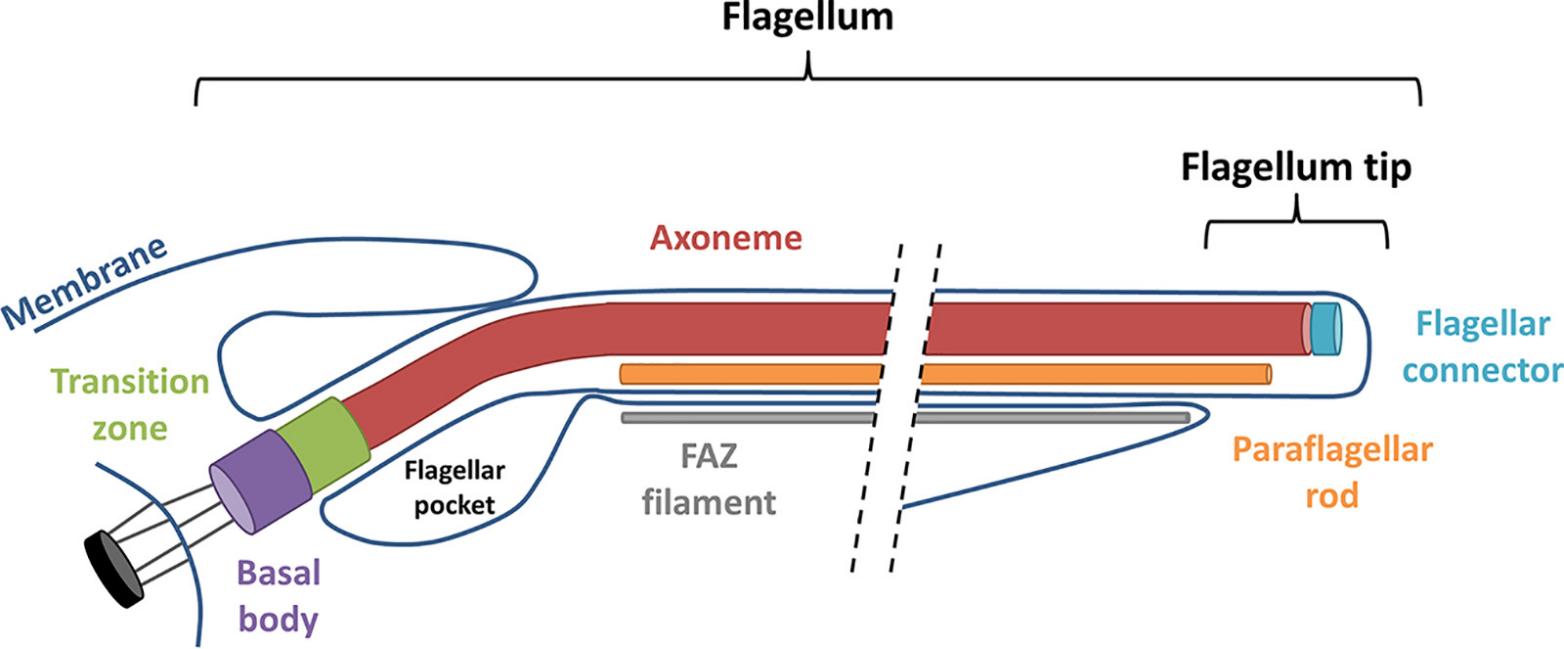
Schematic diagram of major flagellum substructures in T. brucei. Schematic depicts emergence of the flagellum from the basal body near the cell’s posterior end (left), extending to the cell’s anterior end (right). Major substructures are labeled.

The trypanosome flagellum (Fig. 1) is built on a canonical “9+2” axoneme that originates at the basal body in the cytoplasm near the posterior end of the cell (3). Triplet microtubules of the basal body extend to become doublets in the transition zone, which marks the boundary between the basal body and the 9+2 axoneme (18). The axoneme exits the cytoplasm through a specialized invagination of the plasma membrane, termed the flagellar pocket (FP) (19). As it emerges from the flagellar pocket, the axoneme is attached to an additional filament, termed the paraflagellar rod (PFR) (20), that extends alongside the axoneme to the anterior end of the cell. The axoneme and PFR remain surrounded by flagellar membrane that is distinct from but contiguous with the cell and flagellar pocket membrane. The flagellum is laterally attached to the cell body along its length, except for a small region at the distal tip that extends beyond the cell’s anterior end (21). Lateral flagellum attachment is mediated by proteins in the flagellum and cell body that hold the flagellum and plasma membranes in tight apposition, constituting a specialized flagellar attachment zone (FAZ) that extends from the flagellar pocket to the anterior end of the cell (21, 22).

As in other flagellated eukaryotes, the trypanosome flagellar apparatus (Fig. 1) can be subdivided into multiple subdomains, each having specialized function and protein composition. The FP, for example, demarcates the boundary between the flagellar membrane and cell membrane, which have distinct protein and lipid compositions (19, 23). In T. brucei, the FP is the sole site for endocytosis and secretion, thus presenting a critical portal for host-parasite interaction (19). The basal body functions in flagellum duplication, segregation, and axoneme assembly (24). The region encompassing the transition zone lies between the cytoplasm and flagellar compartment and includes proteins that control access into and out of the flagellum (13, 18). The 9+2 axoneme is the engine of motility (7), while the PFR in trypanosomes is considered to physically influence flagellum beating and to serve as a scaffold for assembly of signaling and regulatory proteins (25–28). The FAZ is a trypanosome-specific structure and is critical for parasite motility and cell morphogenesis (29). The anterior flagellum mediates attachment to the tsetse fly salivary gland epithelium, which is crucial for development into human infectious parasites (30). The flagellum tip marks the site of cleavage furrow initiation during cytokinesis (31), and it is the site of signaling proteins that function in social motility (11, 32).

Given the essential and multifunctional roles of the trypanosome flagellum, much effort has been made to define the protein composition of the organelle. However, although we know a lot about the protein composition of the flagellum as a whole, a critical knowledge gap is the protein composition of individual flagellum subdomains. Classical proteomics approaches typically require purification of the flagellum or subfractions of the flagellum (18, 33–37). This approach is useful and has been used to determine proteomes of the detergent-insoluble transition zone (18) and flagellar connector (34), but it can be cumbersome, is dependent on quality of the purified fraction, and cannot resolve subdomains that are not part of a specific structure that can be purified.

To overcome limitations of conventional flagellum proteomics approaches, we applied APEX2 proximity labeling (38) in T. brucei. APEX2 is an engineered monomeric ascorbate peroxidase that converts biotin-phenol into a short-lived biotin radical that is highly reactive. The biotin-phenol radical interacts with nearby proteins, resulting in covalent attachment of a biotin tag. Biotinylated proteins can be affinity purified with streptavidin and identified by shotgun proteomics, allowing for facile identification of proteins within a specific subcellular location from a complex and largely unfractionated sample (38, 39). Here, we report successful implementation of APEX2 proximity labeling in T. brucei to define the proximity proteome of flagellar proteins that are either distributed along the axoneme or restricted to the tip of the flagellum membrane. Our results establish APEX-based proximity proteomics as a powerful tool for T. brucei, demonstrate that the approach can resolve flagellum subdomains that are not separated by a physical boundary, and support the idea that the flagellum tip subdomain functions in cell signaling.

(This article was submitted to an online preprint archive [40].)

## RESULTS

**FIG 2:**
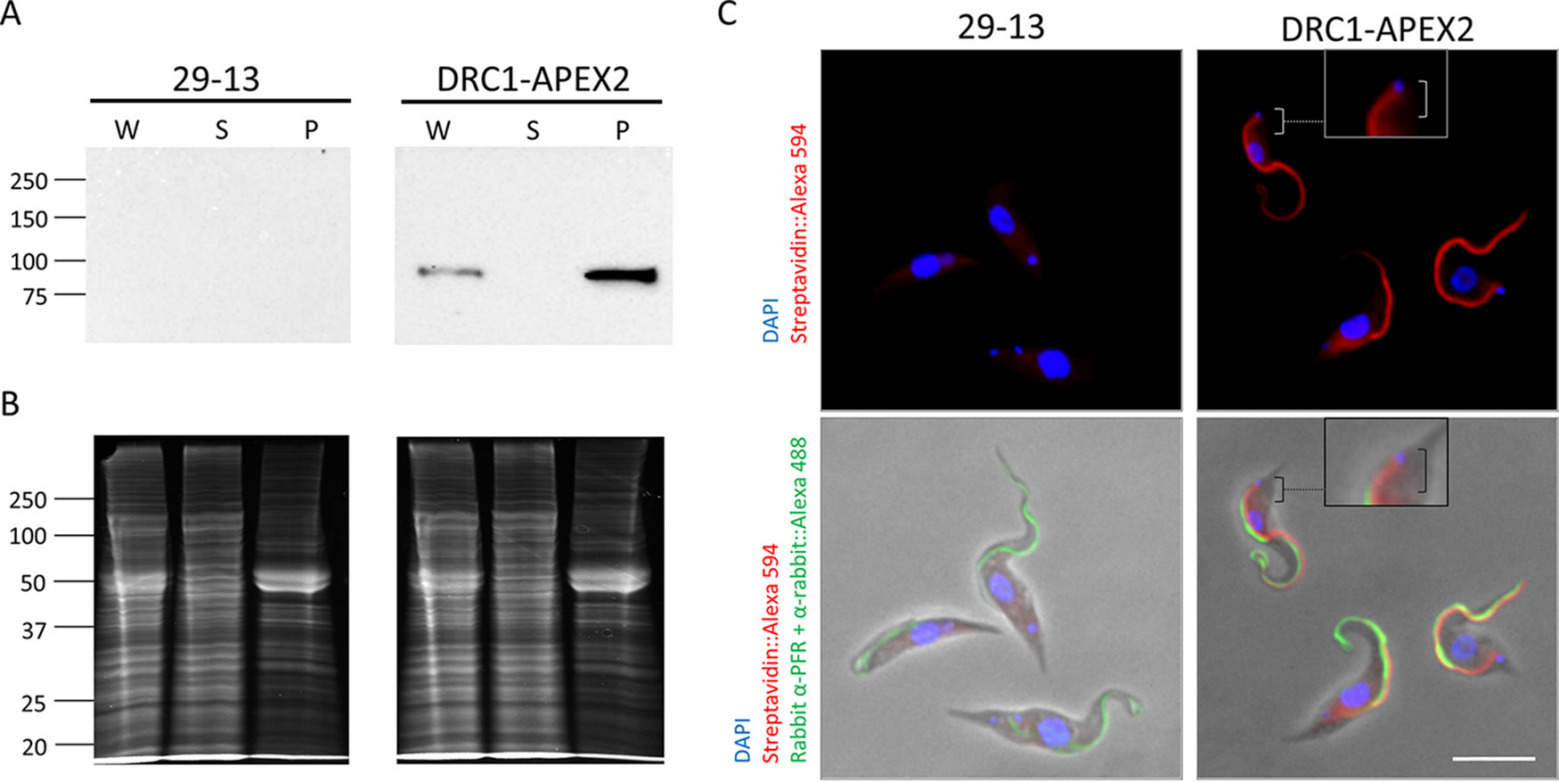
APEX2 directs organelle-specific biotinylation in T. brucei. (A) Western blot of whole-cell lysate (W), NP-40-extracted supernatant (S), and pellet (P) samples from 29-13 and DRC1-APEX2-expressing cells. Samples were probed with anti-HA antibody. (B) Samples in panel A were stained with SYPRO Ruby to assess loading. (C) 29-23 and DRC1-APEX2 cells were examined by immunofluorescence with anti-PFR antibody (Alexa 488, green), streptavidin (Alexa 594, red) and DAPI (blue). Boxes show zoomed-in versions of the cells. Brackets point out that streptavidin (Alexa 594, red) extends up to the kinetoplast. Scale bar, 5 μm.

To evaluate APEX2 labeling in T. brucei, we selected an axonemal protein as bait, because the axoneme is a well-defined cellular component whose protein composition in T. brucei has been examined in prior studies (18, 33, 34, 37). We selected the DRC1 subunit of the nexin-dynein regulatory complex (N-DRC) (41), because this protein has a defined localization along the axoneme (K. L. Hill and G. Langousis, unpublished data) and its position relative to major axonemal substructures, e.g., microtubule doublets, radial spokes, and dynein arms, is known (42). Having selected DRC1 as bait, we used *in situ* gene tagging (43) to generate cell lines expressing DRC1 fused to a C-terminal APEX2 tag that includes APEX2 followed by 3× hemagglutinin (3×HA), referred to as DRC1-APEX2. Expression of DRC1-APEX2 was demonstrated in Western blots of whole-cell lysates (Fig. 2A). Extraction with nonionic detergent leaves the axoneme intact in a detergent-insoluble cytoskeleton fraction (see Fig. S1A in the supplemental material) that can be isolated from detergent-soluble proteins by centrifugation (Fig. S1B) (44). We found that DRC1-APEX2 fractionates almost completely with the detergent-insoluble cytoskeleton, as expected for an N-DRC protein (Fig. 2A). Growth curves demonstrated that expression of DRC1-APEX2 does not affect growth of T. brucei *in vitro* (see Fig. S2).

10.1128/mSphere.01090-20.1 FIG S1 Fractionation controls. (A) Western blot of whole-cell lysate (W), NP-40-extracted supernatant (S), and pellet (P) samples from 29-13 cells. Samples were probed with anti-BiP antibody. *, nonspecific binding. (B) Samples in panel A were stained with SYPRO Ruby to assess loading. (C) Western blot of whole-cell lysate (W), NP-40-extracted supernatant (S), and pellet (P) samples from 29-13 cells. Samples were probed with anti-tubulin antibody. (D) Samples in panel C were stained with SYPRO Ruby to assess loading. Download FIG S1, TIF file, 0.2 MB. Copyright © 2021 Vélez-Ramírez et al. 2021 Vélez-Ramírez et al. https://creativecommons.org/licenses/by/4.0/ This content is distributed under the terms of the Creative Commons Attribution 4.0 International license.

10.1128/mSphere.01090-20.2 FIG S2 Growth curves of APEX2-tagged cell lines. Comparison of doubling times of 29-13 (parental cell line), AC1-APEX2, DRC1-APEX2, FS179-APEX2, and AC1Δ45-APEX2 cell lines in suspension culture over the course of 8 days. Cell densities were adjusted daily to 1e6 cells/ml in order to ensure logarithmic growth. Download FIG S2, TIF file, 0.06 MB. Copyright © 2021 Vélez-Ramírez et al. 2021 Vélez-Ramírez et al. https://creativecommons.org/licenses/by/4.0/ This content is distributed under the terms of the Creative Commons Attribution 4.0 International license.

We next asked if APEX2 is functional within the biochemical environment of the T. brucei cell. Cells expressing DRC1-APEX2 were incubated with biotin-phenol, which was then activated with brief H_2_O_2_ treatment followed by quenching with Trolox (a water-soluble vitamin E analog), and l-ascorbate. To assess if APEX2 labeling conditions affected parasite viability, we performed trypan blue exclusion assays. After the biotin-phenol incubation, 99.3% of cells were viable, and after the consecutive H_2_O_2_ treatment, 98.9% were viable.

To assess biotinylation, cells were probed with streptavidin-Alexa 594 and subjected to fluorescence microscopy. As shown in Fig. 2C, we observed APEX2-dependent biotinylation, and this was highly enriched in the flagellum. There was some background staining in the cytoplasm, as revealed by parallel analysis of parental cells lacking the APEX2-tagged protein (Fig. 2C), but flagellum staining was only observed in cells expressing DRC1-APEX2. In the proximal region of the flagellum, the streptavidin signal extended further than the PFR (Fig. 2C), indicating streptavidin labeling is on the axoneme. Therefore, DRC1-APEX2 directs specific biotinylation in the flagellum.

**FIG 3:**
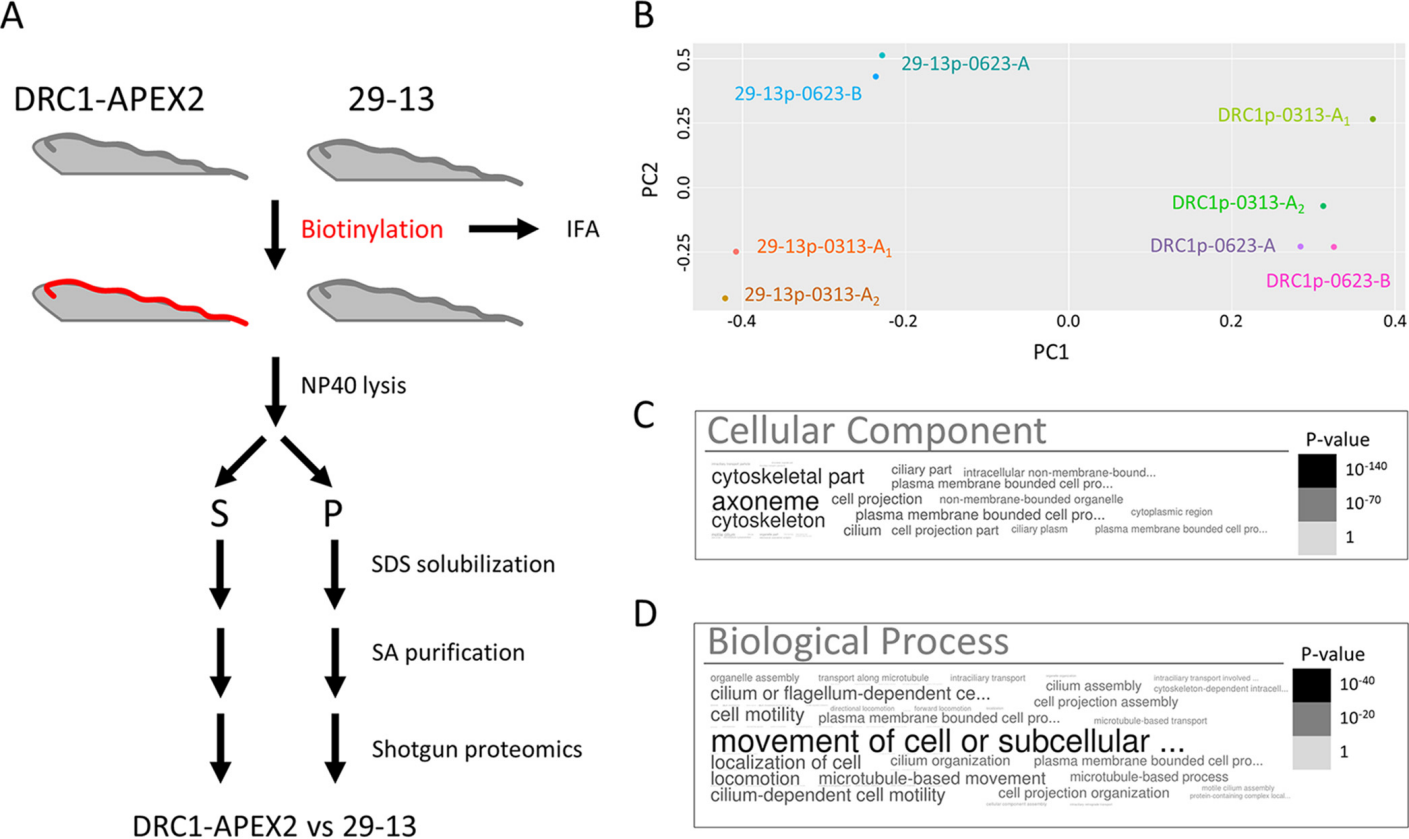
DRC1-APEX2 proximity proteome is enriched for flagellar proteins. (A) Scheme used to identify biotinylated proteins from 29-13 and DRC1-APEX2-expressing T. brucei cells. (B) Principal-component analysis of proteins identified in pellet fractions from 29-13 (29-13p) and DRC1-APEX2 cells (DRC1p). The experiment was performed using three independent biological replicates (0313, 0623-A, and 0623-B), and the 0313 protein sample was split into two aliquots and shotgun proteomics was performed on each in parallel (0313-A1 and 0313-A2). (C and D) Word clouds showing GO analysis of the DRC1p proximity proteome. Size and shading of text reflects the *P* value according to the scale shown.

Having established that DRC1-APEX2 directs flagellum-specific biotinylation, we used shotgun proteomics to identify biotinylated proteins. Samples were extracted with nonionic detergent and separated into detergent-soluble supernatant and detergent-insoluble pellet fractions. Biotinylated proteins in each fraction were then isolated using streptavidin purification and subjected to shotgun proteomics for protein identification. DRC1-APEX2 cells were processed in parallel with the parental cell line (29-13) (45) as a control (Fig. 3A). Our focus was the detergent-insoluble pellet because this fraction includes the axoneme and PFR, and the protein composition of these structures has been characterized (21, 28, 33, 37, 46). The pellet fraction also includes nonaxonemal structures such as the basal body, tripartite attachment complex, FAZ filament, and subpellicular cytoskeleton, thus enabling us to test for enrichment of axonemal proteins. The analysis was performed using three independent biological replicates. In one case, the sample was split into two aliquots, and shotgun proteomics was performed on both in parallel, giving a total of four replicates each for DRC1-APEX2 and 29-13 (control) pellet samples.

Principal-component analysis demonstrated that the biotinylated protein profile of the DRC1-APEX2 pellet samples (DRC1p) was distinct from that of 29-13 pellet samples (29-13p) processed in parallel (Fig. 3B). We detected bona fide axonemal proteins, including DRC1 and axonemal dynein subunits in some 29-13 replicates, but these were enriched in DRC1-APEX2 samples relative to that in 29-13 samples. We therefore assembled a “DRC1p proximity proteome” that included only proteins meeting the following three criteria: (i) detected in all four replicates of the DRC1p sample, (ii) had a normalized spectrum count of two or more, and (iii) were enriched in the DRC1p versus 29-13p sample. This yielded a DRC1p proximity proteome of 697 proteins (Supplemental Table S1). The DRC1p proximity proteome included all known DRC subunits, except DRC6, for which there is no clear T. brucei homologue. Human homologues were identified for 372 proteins in the DRC1p proximity proteome, and among these, 38 are linked to human diseases that have been connected to cilium defects (Table S2). This exemplifies that the T. brucei flagellum could be used as a model system for cilia in other organisms.

10.1128/mSphere.01090-20.7 TABLE S1 (A) Proteins in the DRC1p proximity proteome that were not identified in prior flagellum proteome studies. (B) Proteins in the DRC1p proximity proteome that were identified in prior flagellum proteome studies. Download Table S1, PDF file, 0.2 MB. Copyright © 2021 Vélez-Ramírez et al. 2021 Vélez-Ramírez et al. https://creativecommons.org/licenses/by/4.0/ This content is distributed under the terms of the Creative Commons Attribution 4.0 International license.

10.1128/mSphere.01090-20.6 TABLE S2 Human homologues with disease association. E values come from running the Python script to identify human homologs of the DRC1p proximity proteome genes. *, no specific phenotype on NCBI. Download Table S2, PDF file, 0.2 MB. Copyright © 2021 Vélez-Ramírez et al. 2021 Vélez-Ramírez et al. https://creativecommons.org/licenses/by/4.0/ This content is distributed under the terms of the Creative Commons Attribution 4.0 International license.

To evaluate whether APEX proximity labeling was effective in identifying flagellar proteins, we used Gene Ontology (GO) analysis (47), comparison to prior T. brucei flagellar proteomes (33, 35, 37), and independent tests of localization (48). GO analysis demonstrated significant enrichment of flagellar proteins in the DRC1p proximity proteome compared to those encoded in the genome as a whole (Fig. 3C and D). As discussed above, our efforts were focused on the pellet fraction. We did, however, complete GO analysis on the detergent-soluble “DRC1s proximity proteome,” which also showed significant enrichment of flagellar proteins as well as signaling proteins (see Fig. S3).

10.1128/mSphere.01090-20.3 FIG S3 Word cloud representing GO analysis for cellular component (A) and biological process (B) of the proteins identified in the DRC1s proximity proteome. Download FIG S3, TIF file, 0.1 MB. Copyright © 2021 Vélez-Ramírez et al. 2021 Vélez-Ramírez et al. https://creativecommons.org/licenses/by/4.0/ This content is distributed under the terms of the Creative Commons Attribution 4.0 International license.

Compared with prior proteomic analyses of T. brucei flagella, the detergent-insoluble DRC1p proximity proteome encompassed a larger fraction (45%) of the flagellum skeleton proteome (33) than of the intact flagellum proteome (36%) (37), perhaps due to the fact that the latter includes detergent-soluble proteins, which are not expected in the DRC1p proximity proteome. As anticipated, minimal overlap was observed with the flagellum surface plus matrix proteome (35), which includes only detergent-soluble proteins.

TrypTag localization data (48) were available for 677 proteins in the DRC1p proximity proteome, and 509 of these (75%) are annotated as having TrypTag localization that includes one or more flagellum structures. The DRC1p proximity proteome includes 346 proteins that were not identified in prior proteomic analyses of the T. brucei flagellum or axoneme fragments (18, 28, 33–35, 37) (Table S1A). TrypTag localization data were available for 333 of these, and 241 (72%) are annotated as having a TrypTag localization to one or more flagellum structures. In some cases, localization was specific to flagellum structures, while in others, the protein showed multiple locations. This finding supports the idea that many of these 346 proteins are bona fide flagellar proteins despite going undetected in earlier flagellum proteome studies. The combined results demonstrate that APEX2 proximity labeling is functional in T. brucei and enables identification of flagellar proteins without the need to purify the flagellum. The data also indicate the protein composition of the T. brucei flagellum is more complex than indicated by earlier studies alone.

**FIG 4:**
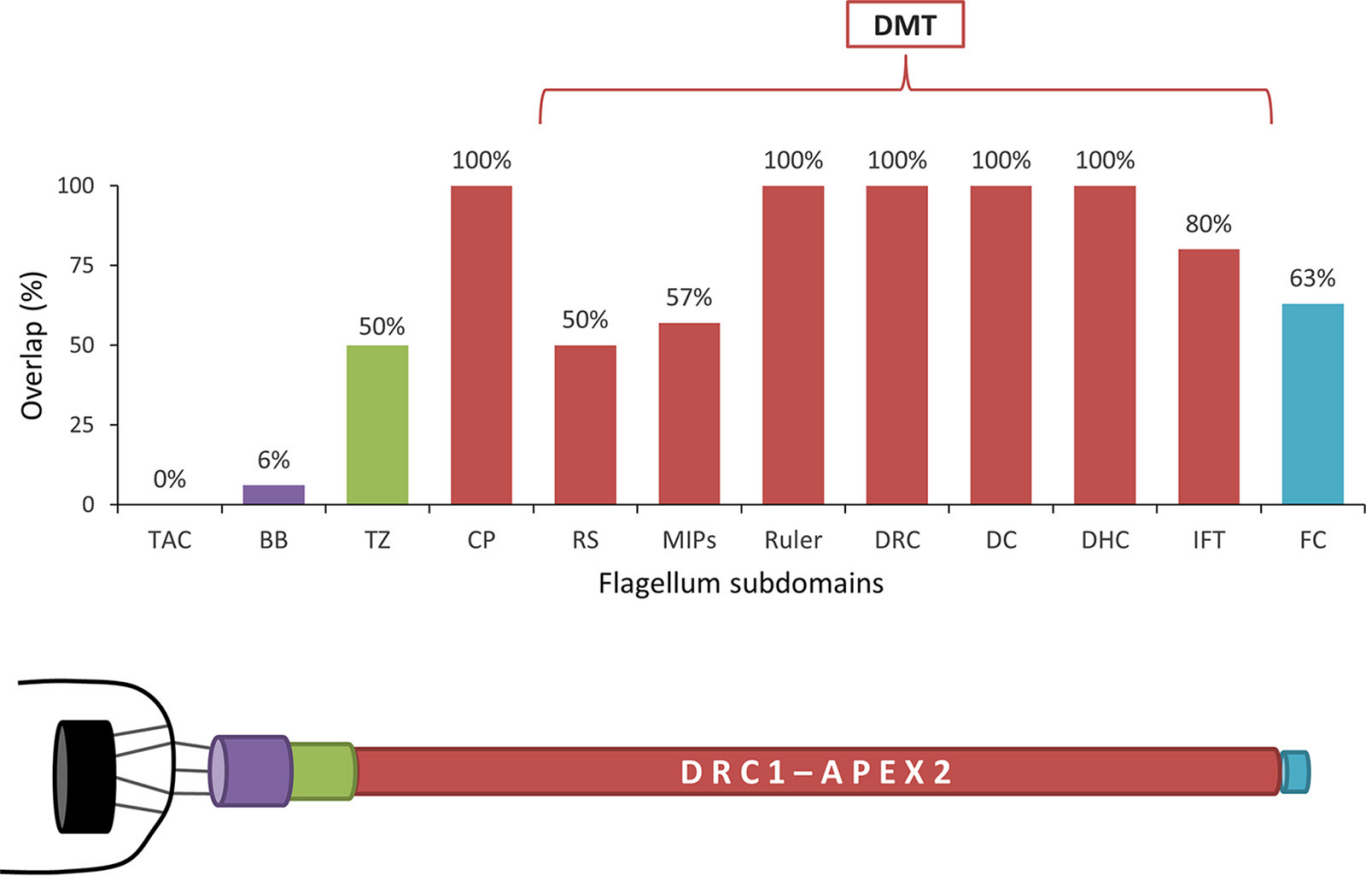
Spatial distribution of known flagellar proteins identified in the DRC1p proximity proteome. Bars in the histogram indicate the percentage of known flagellar proteins from each of the indicated complexes that were identified in the DRC1p proximity proteome. DMT, doublet microtubule; TAC, tripartite attachment complex; BB, basal body; TZ, transition zone; CP, central pair; RS, radial spokes; MIPs, microtubule inner proteins; DRC, dynein regulatory complex; DC, docking complex; DHC, dynein heavy chain; IFT, intraflagellar transport; FC, flagella connector. Schematic below the histogram illustrates the relative position of the complexes indicated in the histogram, with the mitochondrion and kinetoplast indicated in black on the left.

APEX labeling readily distinguishes proteins in close proximity but separated by a membrane (39), and this is evidenced in our data when considering protein components of the FAZ (21). Proteins on the flagellar side of the FAZ are substantially enriched in the DRC1-APEX2 sample, whereas proteins on the cell body side of the FAZ are not (see Fig. S5). Furthermore, the short half-life of the biotin-phenol radical (38) means that APEX labeling can resolve proteins separated by distance even in the absence of a membrane boundary. As discussed above and shown previously (49), APEX resolves flagellar versus cytoplasmic proteins despite these two compartments being contiguous. Within the DRC1p proximity proteome, we noted that proteins distributed similarly to DRC1, i.e., along the entire axoneme, were well represented, while proteins restricted to the distal or proximal end of the flagellum were less represented (Fig. 4 and see Table S3). While total abundance may contribute to this result, it nonetheless suggested, though did not prove, that beyond flagellum versus cytoplasm, APEX labeling might also be able to resolve proteins from different subdomains within the flagellum. We therefore set out to test this idea. We were particularly interested in the flagellum tip because of its importance in trypanosomes and other organisms for signal transduction (10, 11, 50, 51), flagellum length regulation (52–56), and interaction with host tissues (9, 57).

10.1128/mSphere.01090-20.8 TABLE S3 Proteins used for Fig. 4. Download Table S3, PDF file, 0.1 MB. Copyright © 2021 Vélez-Ramírez et al. 2021 Vélez-Ramírez et al. https://creativecommons.org/licenses/by/4.0/ This content is distributed under the terms of the Creative Commons Attribution 4.0 International license.

**FIG 5:**
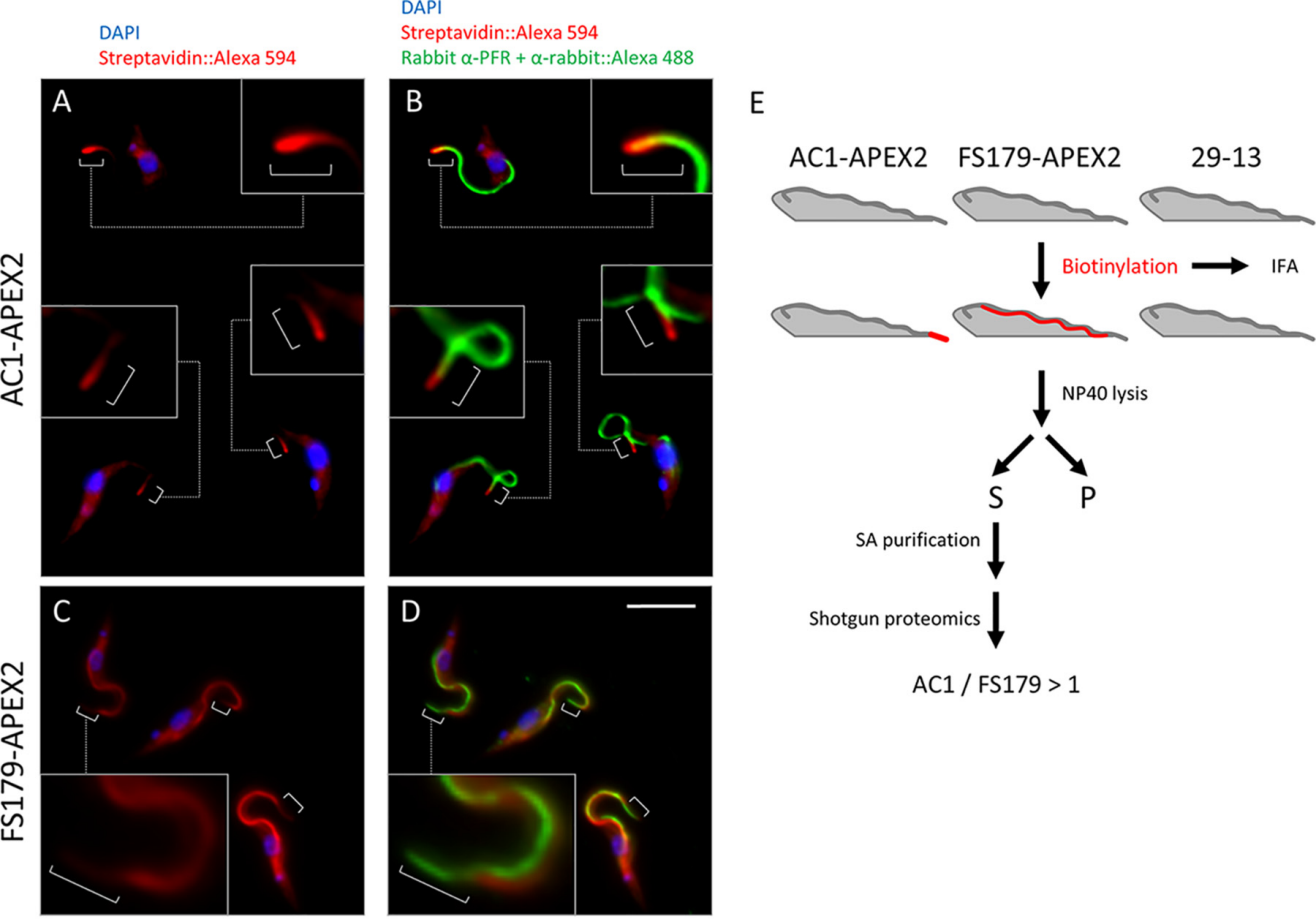
APEX2 labeling resolves flagellum subdomains. (A and B) AC1-APEX2 cells were fixed and examined by fluorescence microscopy after staining with anti-PFR antibody (Alexa 488, green), streptavidin (Alexa 594, red), and DAPI (blue). Boxes show zoomed in version of the cells. Brackets point out streptavidin (Alexa 594, red) at the flagellum tip. (C and D) FS179-APEX2 cells were examined by immunofluorescence with anti-PFR antibody (Alexa 488, green), streptavidin (Alexa 594, red) and DAPI (blue). White brackets indicate the distal region of the flagellum that is not labeled by streptavidin. Boxes show zoomed in versions of the cells. Brackets indicate that streptavidin (Alexa 594, red) is excluded from the flagellum tip. Scale bar, 5 μm. (E) Scheme used to identify biotinylated proteins from the indicated cell lines (29-13, AC1-APEX2, and FS179-APEX2).

To assess whether APEX2 could be used to distinguish between flagellum subdomains, we generated APEX2-tagged versions of a flagellar membrane protein that is tip specific, AC1 (32), and a FAZ protein that is tip excluded, FS179 (35). Expression of either AC1-APEX2 or FS179-APEX2 did not affect T. brucei doubling time (Fig. S2), and both tagged proteins fractionated in the detergent-soluble fraction as expected (see Fig. S4). To assess biotinylation, AC1-APEX2- and FS179-APEX2-expressing cells were incubated with biotin-phenol, activated with H_2_O_2_, and then quenched, probed with streptavidin-Alexa 594, and examined by fluorescence microscopy (Fig. 5A to D). The signal in AC1-APEX2 expressors was enriched at the flagellum tip, while the signal in FS179-APEX2 expressors was distributed along the flagellum but lacking or diminished at the flagellum tip (Fig. 5A to D). These biotinylation patterns match with the previously published localizations for AC1 (32) and FS179 (35). Samples were solubilized with detergent and centrifuged to remove insoluble material. Biotinylated proteins were isolated from the soluble fraction by streptavidin affinity purification and then identified by shotgun proteomics. Negative-control samples were processed in parallel (Fig. 5E) from parental cells (29-13) without an APEX2 tag and cells expressing AC1Δ45-APEX2, an AC1 truncation that lacks the C-terminal 45 amino acids (aa) and is localized to the cytoplasm instead of the flagellum (32).

10.1128/mSphere.01090-20.4 FIG S4 AC1-APEX2, AC1Δ45-APEX2, and FS179-APEX2 NP-40 fractionation. (A) Western blot of whole-cell lysate (W), NP-40-extracted supernatant (S), and pellet (P) samples from 29-13 and AC1-APEX2- and AC1Δ45-APEX2-expressing cells. Samples were probed with anti-HA antibody. (B) Samples in panel A were stained with SYPRO Ruby to assess loading. (C) Slot blot of whole-cell lysate (W), NP-40-extracted supernatant (S), and pellet (P) samples from 29-13 and FS179-APEX2-expressing cells. Samples were probed with anti-HA antibody. (D) Samples in panel A were examined by SDS-PAGE and stained with SYPRO Ruby to assess loading. Download FIG S4, TIF file, 0.4 MB. Copyright © 2021 Vélez-Ramírez et al. 2021 Vélez-Ramírez et al. https://creativecommons.org/licenses/by/4.0/ This content is distributed under the terms of the Creative Commons Attribution 4.0 International license.

10.1128/mSphere.01090-20.5 FIG S5 FAZ proteins identified in the DRC1p proximity proteome. (A) Schematic of the FAZ. (B) FAZ zone to which each protein has been localized, as well as the ratio of spectra identified in the DRC1p versus 29-13p sample. FAZ schematic and zone locations are based on reference 21. Download FIG S5, TIF file, 0.1 MB. Copyright © 2021 Vélez-Ramírez et al. 2021 Vélez-Ramírez et al. https://creativecommons.org/licenses/by/4.0/ This content is distributed under the terms of the Creative Commons Attribution 4.0 International license.

**FIG 6:**
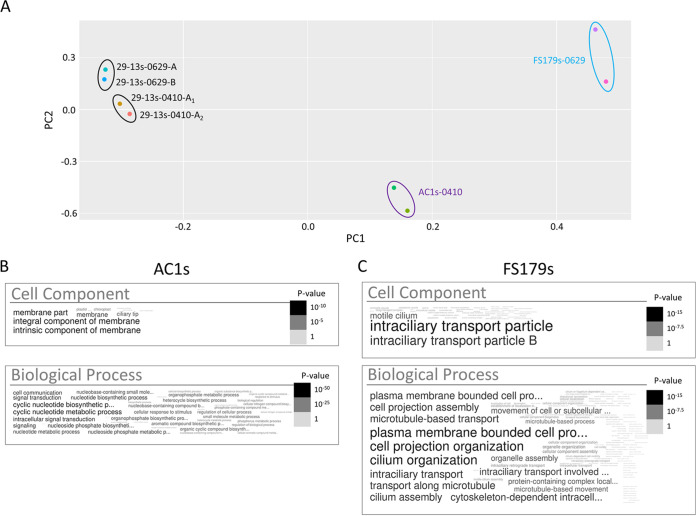
APEX2 proximity proteomics differentiates protein compositions of flagellum subdomains. (A) Principal-component analysis of proteins identified in supernatant fractions from 29-13 (29-13s), AC1-APEX2 (AC1s), and FS179-APEX2 (FS179s) cells. For parental cell line controls (29-13), three independent experiments are shown (0410, 0629-A, and 0629-B), and for one experiment, the sample was split into two aliquots (0410-A1 and 0410-A2) that were subjected to shotgun proteomics in parallel. For FS179s, two independent experiments are shown (0629-A and 0629-B). For AC1s, one sample was split into two aliquots that were subjected to shotgun proteomics in parallel (0410-A1 and 0410-A2). (B) Word cloud representing GO analysis for cellular components and biological processes of the proteins identified in the AC1s proximity proteome. (C) Word cloud representing GO analysis for cellular components and biological processes of the proteins identified in the FS179s proximity proteome.

Principal-component analysis demonstrated that the biotinylated protein profile of AC1-APEX2 detergent-soluble (AC1s) samples was readily distinguished from that of FS179-APEX2 (FS179s) and 29-13 (29-13s) control samples (Fig. 6A). Therefore, APEX2 proximity proteomics was able to distinguish protein compositions of the tip versus FAZ subdomains within the flagellum, even though they have no physical barrier between them.

To define an “AC1s proximity proteome,” we compared the biotinylated protein profile of the AC1 detergent-soluble sample (AC1s) to that of 29-13 (29-13s) and AC1Δ45s samples processed in parallel. We used known flagellum tip proteins (Table 1) to set thresholds for AC1s versus 29-13s and AC1s versus AC1Δ45s enrichment; only proteins exceeding these enrichment thresholds were included in the AC1s proximity proteome. Additionally, we included only those proteins that were enriched in AC1s versus FS179s and DRC1s. This yielded a final AC1s proximity proteome of 48 proteins, including 27 adenylate cyclases and 21 additional proteins (Table 2). Extensive sequence identity among adenylate cyclases poses challenges for distinguishing between some isoforms, and so the number of 27 might be an overestimate. Nonetheless, there is evidence of nonredundant function (11), N-terminal sequence differences (32), and differential expression patterns (36, 58, 59). We performed a parallel analysis to define an “FS179s proximity proteome.” in this case comparing FS179s to 29-13s and AC1Δ45s samples as negative controls, and using (14) intraflagellar transport (IFT) proteins (60) to set the enrichment thresholds for inclusion (Table 3). The reasoning behind using IFT proteins is that they are known flagellar proteins that are found along the flagellum and not enriched at the tip (35). GO analysis showed enrichment of flagellar proteins in both AC1s and FS179s proximity proteomes; however, the AC1s proximity proteome was also enriched with signaling proteins, while the FS179s proximity proteome was not (Fig. 6B and C).

**TABLE 1:** AC1s proximity proteome filtering settings

Sample	Ratio^*a*^									
	Threshold^*b*^	Known tip proteins								
		FLAM8^*c*^ (Tb927.2.5760)	AC2^*d*^ (Tb927.10.16190)	AC3^*e*^ (Tb927.7.7470)	AC4^*d*^ (Tb927.10.13040)	AC5^*d*^ (Tb927.11.13740)	AC6^*f*^ (Tb927.9.15660)	Tip kinesin/KIN-E^*g*^ (Tb927.5.2410)	Calpain 1.3^*h*^ (Tb927.1.2120)	Calpain 9.1^TrypTag^ (Tb927.9.7540)
AC1s/29-13s	>3	3.7	141.08	10.47	a/0^*i*^	a/0	21.6	173	7.66	35.05
AC1s/Δ45s	>2	2.7	3	13.7	a/0	a/0	5.1	43.8	9	4.16
AC1s/FS179s	>1	5	3.53	2.31	5.51	1.74	3.67	1.16	3.6	3.02
AC1s/DRC1s	>1	2.9	2.8	2	3.6	1.3	2.6	1.36	3.1	2.4

a Ratio is a comparison of the average normalized spectral count of all experiments and replicates of the indicated samples.

b Threshold for inclusion in the AC1s proximity proteome based on known tip proteins.

c Reference 37.

d Reference 32.

e Reference 91.

f Reference 11.

g Reference 61.

h Reference 62.

i a/0 (divided over zero), not found in the control (29-13s or Δ45s).

**TABLE 2:** AC1s proximity proteome^*a*^

TriTryp GeneID	TriTryp annotation	Localization^*b*^	Reference and/or source for location data	AC1s/FS179s avg ratio^*c*^
Tb927.2.5860	Hypothetical	Tip	TrypTag (48)	15.88
Tb927.2.5870	Hypothetical	Tip	TrypTag (48, and this work)	10.71
Tb927.4.1500	RNA editing associated helicase 2	Not flagellar	TrypTag (48)	7.45
Tb927.4.4400	Hypothetical	Flagellum, distal points plus endocytic	TrypTag (48)	5.18
Tb927.2.5760	Flagellar member 8	Tip	37	5.03
Tb927.11.14410	Ankyrin repeats	Not flagellar	TrypTag (48)	3.89
**Tb927.1.2120**	**Calpain-like protein CALP1.3**	**Tip**	62	**3.60**
**Tb927.11.17040**	**AC1**^*d*^	**Tip**	32	**3.37**
Tb927.7.4060	Cysteine peptidase, clan CA, family C2	Flagellum, tip enriched plus cytoplasm	TrypTag (48, 62)	3.02
Tb927.9.7540	Cysteine peptidase, clan CA, family C2, putative	Flagellum, tip enriched	TrypTag (48, and this work)	2.98
Tb927.7.4070	Cysteine peptidase, clan CA, family C2	Tip plus cytoplasm	62	2.86
**Tb927.7.5340**	**cAMP response protein 3**	**Flagellum, tip enriched plus cytoplasm**	**TrypTag (**48**, and this work)**	**2.41**
Tb927.10.15700	Hypothetical	Tip		2.11
**Tb927.4.4220**	**Small GTP-binding rab protein**	**Flagellar pocket**	**TrypTag (**48**)**	**2.11**
Tb927.10.9380	SEP domain/UBX domain containing protein	New flagellum-distal points	TrypTag (48)	1.86
Tb927.5.2090	Kinesin (KIN2A)	Flagellum, tip (weak) plus cytoplasm	TrypTag (48)	1.74
**Tb927.6.4710**	**Calmodulin**	**Flagellum**	**TrypTag (**48**)**	**1.68**
Tb927.3.4640	VIT family	Not flagellar	TrypTag (48)	1.50
Tb927.7.7260	Kinesin (KIF9B)	Flagellum puncta, basal and probasal body	92	1.32
Tb927.9.9690	Hypothetical	Flagellum, tip enriched	TrypTag (48)	1.22
Tb927.7.3090	Galactose oxidase, central domain containing protein, putative	Not flagellar	TrypTag (48)	1.17
Tb927.5.2410	(Tip) kinesin (KIN-E)	Flagellum, tip enriched	TrypTag (48, 61)	1.16

a Proteins in boldface font have a known role in cell signaling.

b Tip, the prominent or sole location is flagellum tip; flagellum, tip enriched, in the flagellum and enriched at tip; tip enriched, enriched at the flagellum tip, but also present outside the flagellum.

c Normalized spectral count average of all experiments and replicates of AC1s over normalized spectral count average of all experiments and replicates of FS179s. Only proteins with AC1/FS179 ratio of >1 are included in the AC1s proteome.

d AC1 represents a total of 27 ACs identified in the AC1s proximity proteome (see Table S4 in the supplemental material).

**TABLE 3:** FS179s proximity proteome filtering settings

Sample	Ratio^*a*^										
	Threshold^*b*^	Known flagellar proteins									
		IFT121^TrypTag^ (Tb927.5.3030)	IFT122^TrypTag^ (Tb927.10.5380)	IFT140^TrypTag^ (Tb927.10.14470)	IFT172^TrypTag,^ ^*c*^ (Tb927.10.1170)	IFT52^TrypTag^ (Tb927.10.14980)	IFT57/55^TrypTag^ (Tb927.10.11310)	IFT74^TrypTag^ (Tb927.7.3370)	IFT80^TrypTag^ (Tb927.10.14990)	IFT81^TrypTag^ (Tb927.10.2640)	IFT88^TrypTag^ (Tb927.11.1740)
FS179s/29-13s	>10	10.44	12.51	97.46	26.04	11.69	a/0^*d*^	a/0	a/0	a/0	a/0
FS179s/Δ45s	>10	a/0	a/0	a/0	11.09	a/0	a/0	a/0	a/0	a/0	a/0

a Ratio is a comparison of the average normalized spectral count of all experiments and replicates of the indicated samples.

b Indicates the threshold for inclusion in the FS179s proximity proteome based on known flagellar proteins.

c Reference 60.

d a/0 (divided over zero), not found in the control (29-13s or Δ45s).

Prevalence of adenylate cyclases within the AC1s proximity proteome supports the idea that the data set is enriched for tip proteins, because all T. brucei adenylate cyclases studied to date are flagellar and, in procyclics, many are enriched at the flagellum tip (32). AC2 is localized all along the flagellum (32), yet it is found in the AC1s proximity proteome, perhaps due to the fact that AC2 and AC1 dimerize and share ∼90% amino acid sequence identity (32).

**FIG 7:**
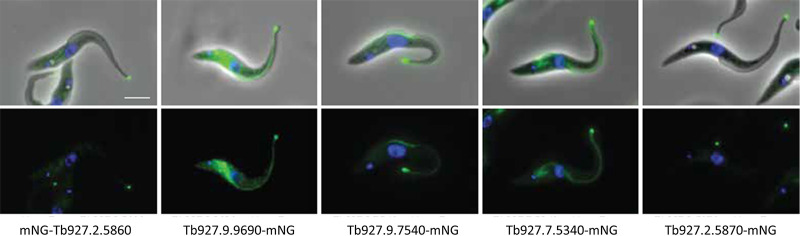
AC1s proximity proteome identifies tip proteins. Fluorescence microscopy of trypanosomes expressing the indicated protein tagged with mNeonGreen (green). Samples are stained with Hoechst 33342 (blue). Top panel shows fluorescence plus phase contrast merged images. Bottom panel shows fluorescence image. Scale bar, 5 μm.

Among the 21 non-AC proteins in the AC1s proximity proteome, 20 have independent data on localization (48). Four of these have previously been published as being flagellum tip specific (FLAM8 and CALP1.3), flagellum specific and tip enriched (KIN-E), or located throughout the cell but also found in the flagellum tip (CALP7.2) (37, 61, 62). For the remaining 16 proteins, we assessed localization by referencing the TrypTag database (48) and/or epitope tagging directly. We find that half of these 16 proteins are either tip specific or enriched at the flagellum tip while also being located elsewhere in the cell (Fig. 7 and Table 2). Notably, most of the proteins that did not exhibit tip localization were among the least enriched in the AC1s samples versus those in FS179s samples (Table 2). Among 11 proteins enriched >2-fold in AC1s versus FS179s and having localization data, 9 are enriched in the flagellum tip. Therefore, the AC1s proximity proteome is enriched for flagellum tip proteins.

## DISCUSSION

The protein composition of flagellum subdomains in T. brucei is a knowledge gap in understanding the biology of these pathogens. To overcome this, we implemented APEX2 proximity proteomics. Our results demonstrate that APEX-based proximity labeling is effective in T. brucei and is capable of resolving flagellum subdomains, even if they are not separated by physical barriers. Use of the APEX system has allowed us to define a soluble flagellum tip proteome that includes signaling proteins and supports the idea that the tip participates in cAMP signaling (10, 11). While this tip proteome is likely incomplete, our work represents an important step in defining the protein composition of flagellum subdomains and other trypanosome cellular compartments that cannot themselves be purified.

One major advantage of proximity labeling-based proteomics versus other proteomic approaches to define organelle protein composition is that it allows for isolation of proteins of interest from crude cell lysates in a simple one-step purification, without the need to purify the organelle. Prior proteomic analyses of the T. brucei flagellum have required purification of the flagellum away from the cell body (33, 35, 37). This is problematic, because the flagellum in T. brucei is laterally connected to the cell body along most of its length. Therefore, purification approaches have employed genetic manipulation to remove lateral connections followed by sonication or shearing to detach the flagellum at its base (35, 37) or have employed detergent extraction followed by selective depolymerization of subpellicular microtubules while leaving axoneme microtubules intact (33). The latter approach does not allow for identification of flagellum matrix or membrane proteins that are detergent soluble. In both cases, flagellum detachment is followed by centrifugation to separate flagellum fractions from cell bodies and solubilized material and then electron microscopy to evaluate sample quality. The proximity labeling-based proteomics eliminates the need for subcellular fractionation and evaluation of the purified flagellum sample, although it can be coupled with fractionation to distinguish detergent-soluble from -insoluble proteins, as in our case. Such approaches also enable easy detection of detergent-soluble and -insoluble proteins from the same cells. A disadvantage of proximity labeling-based proteomics is that, owing to the requirement for proteins to be close to the APEX-tagged bait, one might miss proteins that are part of the organelle in question but distant from the bait protein. For this reason, efforts to define a comprehensive whole-organelle proteome should employ multiple independent and complementary approaches.

BioID (39, 63–65) is an alternative proximity-labeling method that employs a promiscuous biotin ligase, BirA*, and has been used widely, including several applications in T. brucei (66–69) and other parasites (70). BirA* and APEX-based proximity labeling methods are complementary, achieving similar aims with overlapping but also distinct mechanistic features (71). BioID offers the advantage of using easily deliverable biotin, while APEX offers the capacity for doing short-time-scale time course analyses owing to the short (30 s to 1 min) activation period required to initiate labeling (38). Traditional BioID requires several hours of incubation with biotin to achieve labeling (65). A modified version, termed TurboID, allows for a shorter (10 min) incubation period, enabling time course studies (72), although TurboID has not yet been validated in trypanosomes. Beyond proximity proteomics, APEX catalyzes oxidation of 3,3′-diaminobenzidine (DAB), which polymerizes and can be visualized by light microscopy or electron microscopy to determine precise subcellular location of the APEX-tagged protein (73).

Our ability to distinguish protein profiles of AC1s and FS179s samples (Fig. 6A) illustrates the capacity of APEX to distinguish between subcellular regions that are not separated by a physical barrier. Although this capacity was shown previously in mammalian cells for the cilium compartment versus the cytoplasm (49) and for distinct locations in the cytoplasm (74), our studies now demonstrate this is also possible within the spatially restricted volume of the flagellum. The eukaryotic flagellum is composed of specific subdomains, each with specialized functions and protein compositions (32). The distal tip of the flagellum in many organisms, for example, is important for the transduction of extracellular signals, flagellum length regulation, and cell-cell adhesion (10, 11, 50–56). The transition zone at the flagellum’s proximal end has specialized functions, controlling access into and out of the flagellar compartment (18, 75). Even dyneins are distributed differentially from proximal to distal ends of the axoneme (76). APEX labeling has previously been used to identify proteins throughout the cilium in mammalian cells (49). To our knowledge, however, our studies are the first to extend this system to distinguish protein compositions of specific flagellum subdomains, thus providing a powerful addition to tools available for dissecting flagellum function and mechanisms of ciliary compartmentation.

Prior work has provided important advances by determining the protein compositions of two specific subdomains within the T. brucei flagellum, the transition zone (18) and tip (34) of the detergent-insoluble axoneme, the latter including an axonemal capping structure (ACS) and the T. brucei-specific flagellar connector (FC) (34). Those studies developed a novel method termed structure immunoprecipitation (SIP), in which detergent-insoluble axonemes are prepared and then fragmented into small pieces and immunoprecipitated using an antibody to a marker protein localized to the domain of interest. Independent localization, biochemical, and functional analyses were then used to define the corresponding transition zone, ACS, and FC proteomes. Given that the FC and ACS are at the tip of the flagellum, we compared their proteomes to the AC1s proximity proteome. We did not find any overlap, as might be expected because the FC and ACS analyses were restricted to detergent-insoluble components, while the AC1s proximity proteome uniquely identifies detergent-soluble proteins. Our work therefore complements and extends earlier proteome analyses of T. brucei flagellum subdomains and expands our knowledge of proteins that mediate flagellum tip-specific functions.

The AC1s proximity proteome is enriched with known and previously unknown flagellum tip proteins (Fig. 7 and Table 2). Function for many of these proteins remains to be determined, but for some, there is existing information to suggest a role in signal transduction. One, previously identified as cAMP response protein 3 (CARP3), is a candidate cAMP effector (77), and more than half of the proteins are adenylate cyclases. A role for the flagellum tip in cAMP signaling was previously demonstrated (10, 11), and flagellar cAMP signaling is required for the T. brucei transmission cycle in the tsetse fly (12). Another protein group well represented is calpain-like proteins. Calpains function in Ca^2+^ signal transduction, although the calpain-like proteins in the AC1s proximity proteome possess only a subset of the domains typically seen in more classical calpains. Interestingly, the flagellar tip kinesin KIN-E (61) contains a domain III-like domain typically found in calpains and thought to function in lipid binding (78).

Beyond signaling functions, the AC1s proximity proteome offers insight into other aspects of flagellum tip biology. It includes four proteins annotated as having homology to a serine-rich adhesin protein from bacteria (79). Whether these function as adhesins in the flagellum is unknown, but adhesion functions could contribute to attachment of the axoneme to the flagellar membrane or in attachment functions of the FC (80). KIF9B was previously detected at the flagellum tip in IFT knockdowns (81) and, its presence in the AC1s proximity proteome (Table 2) suggests it might be transiently present at the flagellum tip under normal conditions. The mechanism by which IFT kinesins return to the flagellum base after remodeling at the tip is an important and debated topic, which may differ between different organisms (53). Presence of the IFT kinesin KIN2A (82), but not other IFT components, in the AC1s proximity proteome and its distribution along the flagellum with slight enrichment at the flagellum tip (Table 2) might indicate that KIN2A diffuses back to the base, similar to what has been observed in Chlamydomonas reinhardtii (83) but different from the retrograde transport observed in Caenorhabditis elegans (84).

The DRC1p proximity proteome showed substantial overlap with earlier proteome analyses of purified flagellum skeletons (18, 33, 34, 37) while also including 346 proteins not found in those prior studies. Independent data on 241 of these 346 proteins support flagellum association for 72%, indicating the protein composition of the T. brucei flagellum is more complex than suggested from earlier studies. Many proteins uncovered in prior flagellum proteome analyses were not found in the DRC1p proximity proteome. There are several potential explanations for this. First, some earlier studies included detergent-soluble matrix and membrane proteins (35, 37), which are not expected in the DRC1p proximity proteome. Second, the threshold for inclusion in the DRC1p data set is high, as proteins must be present in all four DRC1p replicates and enriched over that of negative controls, while some of the earlier studies examined a limited number of replicates (33) or lacked extensive negative controls (35). Third, each approach will contain false positives and false negatives. For example, flagellar proteins that are far away from DRC1 might not be biotinylated in our analysis. We recognize that several bona fide axonemal proteins were identified in 29-13 control samples processed in parallel to DRC1p samples. We do not presently know the reason for this, but the problem was less evident in the analysis of detergent-soluble AC1s samples and so may relate to residual insolubility of the axoneme or high abundance of axoneme proteins, which may be present at thousands of copies per axoneme.

Among proteins identified here, but not in prior flagellum proteomes, the DRC1p proximity proteome includes several kinesins with unspecified functions, suggesting flagellum roles. Several nucleoporins were also identified, which may inform the debate regarding a flagellum role for these proteins (85). The relatively high representation of IFT proteins is interesting, considering that, with the exception of the IFT dynein complex (86), few IFT cargoes have been identified in T. brucei. In C. reinhardtii, DRC4 is transported as an IFT cargo (87). Thus, if DRC1 in T. brucei is transported as an IFT cargo, this could explain the prevalence of IFT proteins.

To our knowledge, this is the first work to demonstrate APEX2 proximity proteomics in a eukaryotic human pathogen. This is an important point, because systems for removing reactive oxygen species in any given organism may limit suitability of APEX-based labeling (39). For example, erythrocytic *Plasmodium* spp. rely on a series of redundant glutathione- and thioredoxin-dependent reactions to remove H_2_O_2_ and maintain redox equilibrium (88). Therefore, our studies expand the capacity in T. brucei for proximity labeling, which is an increasingly important tool in postgenomic efforts to define protein location and function in pathogenic organisms (71).

## MATERIALS AND METHODS

### Trypanosoma brucei culture.

Procyclic Trypanosoma brucei *brucei* (strain 29-13) (45) was used as a control and to generate all the APEX2-tagged cell lines. Cells were cultivated in SM medium supplemented with 10% heat-inactivated fetal bovine serum and incubated at 28°C with 5% CO_2_. Cell viability was assessed using trypan blue exclusion (0.4% solution; Lonza), according to the manufacturer’s protocol.

### *In situ* tagging.

APEX2-tagged cell lines were generated by *in situ* tagging (89). In each case, cells were transfected with a cassette containing the APEX2-NES tag (38) followed by a puromycin resistance marker and flanked on the 5′ end by the 3′ end of the target gene open reading frame (ORF) and on the 3′ end by the target gene 3′ untranslated region (UTR). For AC1 (Tb927.11.17040) and FS179 (Tb927.10.2880), the AC1-HA (35) and FS179-HA (32) *in situ* tagging vectors in pMOTag2H (89) were modified by inserting the APEX2-NES tag in-frame between the target gene ORF and 3×HA tag to generate the final APEX2 tagging cassettes. For DRC1 (Tb927.10.7880), the last 592 bp of the ORF were PCR amplified from genomic DNA and cloned upstream of the 3×HA tag in the pMOTag2H (89) vector backbone. Similarly, the first 404 bp of the DRC1 3′ UTR were PCR amplified from genomic DNA and cloned downstream of the puromycin resistance marker. The APEX2-NES tag was then inserted between the target gene ORF and 3×HA tag as described above. All sequences were verified by DNA sequencing. Tagging cassettes were excised from the pMOTag2H backbone (89) and gel purified. Trypanosome cells were transfected by electroporation and selected with 1 μg/ml puromycin as described previously (43).

### Biotinylation.

Biotinylation was performed using a modified version of the method in reference 39. Briefly, cells were harvested by centrifugation for 10 min at 1,200 × *g* and then resuspended at 2 × 10^7^ cells/ml in growth medium supplemented with 5 mM biotin-phenol (biotin tyramide; Acros Organics). After a 1-h incubation, cells were treated with 1 mM H_2_O_2_ for 1 min. To quench unreacted hydrogen peroxide, an equal volume of 2× quenching buffer (10 mM Trolox and 20 mM l-ascorbic acid sodium salt in phosphate-buffered saline [PBS], pH 7.2) was added, cells were harvested by centrifugation, and two additional washes were performed with 1× quenching buffer.

### Immunofluorescence.

Cells were washed once in PBS and fixed by addition of paraformaldehyde to 0.1% for 5 min on ice. Fixed cells were washed once in PBS and air dried onto coverslips. The coverslips were incubated for 10 min in −20°C methanol followed by 10 min in −20°C acetone and then air dried. Cells were rehydrated for 15 min in PBS and blocked overnight in blocking solution (PBS plus 5% bovine serum albumin [BSA] plus 5% normal donkey serum). Coverslips were incubated with streptavidin coupled to Alexa 594 (Life Technologies) and anti-PFR antibody (32) diluted in blocking solution for 1.5 h. After three washes in PBS plus 0.05% Tween 20 for 10 min each, coverslips were incubated with donkey anti-rabbit IgG coupled to Alexa 488 (Invitrogen) in blocking solution for 1.5 h. After three washes in PBS plus 0.05% Tween 20 for 10 min each, cells were fixed with 4% paraformaldehyde for 5 min. Coverslips were washed three times in PBS plus 0.05% Tween 20 and one time in PBS for 10 min each. Cells were mounted with Vectashield containing 4′,6-diamidino-2-phenylindole (DAPI; Vector). Images were acquired using a Zeiss Axioskop II compound microscope and processed using AxioVision (Zeiss, Inc., Jena, Germany) and Adobe Photoshop (Adobe Systems, Inc., Mountain View, CA).

### Fractionation of whole cells and purification of biotinylated proteins.

For purification of biotinylated proteins, 6 × 10^8^ cells were washed once in PBS and lysed in PEME lysis buffer (100 mM PIPES, 1.5 mM Na, 2 mM EGTA, 1 mM MgSO_4_·7H_2_O, and 0.1 mM EDTA-Na_2_·2H_2_O) plus 0.5% Nonidet P-40 (NP-40) plus EDTA-free protease inhibitors (Sigma) for 10 min on ice. Lysates were centrifuged for 8 min at 2,500 × *g* at room temperature (RT) to separate the NP-40-soluble supernatant and the insoluble pellet. The pellet was boiled for 5 min in lysis buffer plus 1% SDS and centrifuged for 3 min at 21,000 × *g* at RT to remove insoluble debris, and the SDS supernatant was collected.

Capture of biotinylated proteins from the NP-40-soluble and SDS supernatants on streptavidin beads was performed essentially as described previously (35). Cell fractions were incubated with 120 μl streptavidin beads (GE Healthcare) overnight at 4°C with gentle agitation. Biotinylated proteins bound to streptavidin beads were separated from the unbound molecules by centrifugation. Beads were washed once with lysis buffer at 4°C, whereas the rest of the washes were performed at RT as follows: once in buffer A (8 M urea, 200 mM NaCl, 2% SDS, and 100 mM Tris), once in buffer B (8 M urea, 1.2 M NaCl, 0.2% SDS, 100 mM Tris, 10% ethanol, and 10% isopropanol), once in buffer C (8 M urea, 200 mM NaCl, 0.2% SDS, 100 mM Tris, 10% ethanol, and 10% isopropanol), and 5 times in buffer D (8 M urea and 100 mM Tris); pH of all wash buffers was 8.

### Shotgun proteomics.

Shotgun proteomics was performed based on reference 35. Streptavidin-bound proteins were digested on beads by the sequential addition of Lys-C and trypsin protease. Peptide samples were fractionated online using reversed-phase chromatography followed by tandem mass spectrometry (MS/MS) analysis on a Thermo Fisher Q-Exactive mass spectrometer. Data analysis was performed using the IP2 suite of algorithms (Integrated Proteomics Applications). Briefly, RawXtract (version 1.8) was used to extract peaklist information from Xcalibur-generated RAW files. Database searching of the MS/MS spectra was performed using the ProLuCID algorithm (version 1.0) and a user assembled database consisting of all protein entries from the TriTrypDB for T. brucei strain 927 (version 7.0). Other database search parameters included (i) precursor ion mass tolerance of 10 ppm, (ii) fragment ion mass tolerance of 10 ppm, (iii) only peptides with fully tryptic ends were considered candidate peptides in the search with no consideration of missed cleavages, and (iv) static modification of 57.02146 on cysteine residues. Peptide identifications were organized and filtered using the DTASelect algorithm, which uses a linear discriminate analysis to identify peptide scoring thresholds that yield a peptide-level false-discovery rate of less than <1.8% as estimated using a decoy database approach. Proteins were considered present in the analysis if they were identified by two or more peptides using the <1.8% peptide-level false-discovery rate.

For principal-component analysis (PCA) and comparison of proteins identified in each sample, proteomics data were parsed using the IP2 Integrated Proteomics Pipeline ver. 5.1.2. The output from the Protein Identification STAT Compare tool (IDSTAT_COMPARE) in IP2 was processed using a custom R-based web app (https://uclaproteomics.shinyapps.io/iscviewer/) to generate the PCA graphs. For proteins identified in different samples, the ID_COMPARE output was exported to Excel to generate mass spectrometry data analysis tables (see Tables S4 and S5 in the supplemental material).

10.1128/mSphere.01090-20.9 TABLE S4 Raw proteomic data of pellet fractions and filtered DRC1p proximity proteome. Download Table S4, XLSX file, 4.1 MB. Copyright © 2021 Vélez-Ramírez et al. 2021 Vélez-Ramírez et al. https://creativecommons.org/licenses/by/4.0/ This content is distributed under the terms of the Creative Commons Attribution 4.0 International license.

10.1128/mSphere.01090-20.10 TABLE S5 Raw proteomic data of supernatant fractions and filtered AC1s and FS179s proximity proteomes. Download Table S5, XLSX file, 12.0 MB. Copyright © 2021 Vélez-Ramírez et al. 2021 Vélez-Ramírez et al. https://creativecommons.org/licenses/by/4.0/ This content is distributed under the terms of the Creative Commons Attribution 4.0 International license.

For the DRC1p proximity proteome, data were from three independent experiments each for DRC1-APEX2 and 29-13 (control) pellet samples. In one case each, the sample was split into two aliquots, and shotgun proteomics was performed on both in parallel, giving a total of four replicates each for DRC1-APEX2 and 29-13 samples. Ratios of average normalized spectra were used to determine inclusion in the DRC1p proximity proteome as described in the text. Of 739 proteins identified, 42 redundant gene identifiers (GeneIDs) were removed for a total proximity proteome of 697 (Tables S1 and S4). For the AC1s and FS179s proximity proteomes, data were from four independent experiments for 29-13 samples (0313, 0410, 0629-A, and 0629-B) and two independent experiments each for AC1 (0313 and 0410) and FS179 (0629-A and 0629-B). For the 0313 and 0410 experiments, each sample was split into two aliquots, and shotgun proteomics was performed in parallel on each, for a total of six replicates for 29-13, four replicates for AC1, and two replicates for FS179. Ratios of average normalized spectra were used to determine inclusion in the AC1s and FS179s proximity proteomes as described in the text.

### Bioinformatics.

To identify human homologues, we developed an algorithm to automatically return reciprocal best blast hits from the large lists of proteins produced by shotgun proteomics. The algorithm was implemented in python using the biopython library (90) and works as follows. Individual sequences were parsed sequentially and used as a query for BLASTp to find a list of similar sequences with an E value threshold of 0.1 from a database of human protein sequences retrieved from NCBI. The top three most similar sequences were then used as query sequences for a subsequent BLASTp call against a database of T. brucei protein sequences. If the original sequence was found in the top three from this call, then the query was then returned as a homologue. The python code for performing this can be found at the following URL: https://github.com/marcusgj13/Reciprocal-BB. Association of the identified homologues with human disease was taken from the phenotype section of the NCBI entry. Comparison to published T. brucei flagellar proteomes (33, 35, 37) was performed using the search tools in the TriTryp Genome Database (https://tritrypdb.org/tritrypdb/) (79). Word clouds (Fig. 3, 6, and S3) were obtained using the GO Enrichment tool of TriTrypDB, using T. brucei *brucei* TREU927 and a 0.05 *P* value cutoff.

To assess protein localization as determined by TrypTag (48), proteins in the DRC1p proximity proteome data set were cross-referenced with TrypTag to look at matches versus mismatches. The GO terms searched for were: flagellum, transition zone, flagellar cytoplasm, probasal body, basal body, flagellar membrane, flagella connector, paraflagellar rod, flagellum attachment zone, intraflagellar transport particle, hook complex, flagellar tip, and axoneme. Among the DRC1p proximity proteome (697 entries), the number of genes for which there is TrypTag localization data for at least one terminus was 677. The number of TrypTag-tagged genes that matched a flagellum GO term was 509. The number of TrypTag-tagged genes that did not match a flagellum GO term was 168. Therefore, of 677 proteins with TrypTag localization data, 75% have a TrypTag localization that matches the query GO terms. Among DRC1p proximity proteome proteins not found in earlier proteome analyses (346 proteins), the number of query genes for which there is TrypTag localization data for at least one terminus was 333. The number of query genes where TrypTag localization matches the query GO term was 241. The number of query genes where TrypTag localization mismatches the query GO term was 96. Therefore, of 333 proteins with TrypTag localization data, 72% have a TrypTag localization that matches the query GO terms.

### Data availability.

The python code used to identify human homologues was deposited in GitHub, and it can be found at the following URL: https://github.com/marcusgj13/Reciprocal-BB. The code, explanation of it, and instructions to install and run the program can also be found there.

## References

[1] Stuart K, Brun R, Croft S, Fairlamb A, Gurtler RE, McKerrow J, Reed S, Tarleton R. 2008. Kinetoplastids: related protozoan pathogens, different diseases. J Clin Invest 118:1301–1310. doi:10.1172/JCI33945. 18382742 PMC2276762

[2] FAO. 2019. Controling tsetse and trypanosomosis to protect African livestock keepers, public health and farmers' livelihoods. FAO, Rome, Italy.

[3] Langousis G, Hill KL. 2014. Motility and more: the flagellum of *Trypanosoma brucei*. Nat Rev Microbiol 12:505–518. doi:10.1038/nrmicro3274. 24931043 PMC4278896

[4] Shimogawa MM, Ray SS, Kisalu N, Zhang Y, Geng Q, Ozcan A, Hill KL. 2018. Parasite motility is critical for virulence of African trypanosomes. Sci Rep 8:9122. doi:10.1038/s41598-018-27228-0. 29904094 PMC6002391

[5] Rotureau B, Ooi CP, Huet D, Perrot S, Bastin P. 2014. Forward motility is essential for trypanosome infection in the tsetse fly. Cell Microbiol 16:425–433. doi:10.1111/cmi.12230. 24134537

[6] Ralston KS, Lerner AG, Diener DR, Hill KL. 2006. Flagellar motility contributes to cytokinesis in Trypanosoma brucei and is modulated by an evolutionarily conserved dynein regulatory system. Eukaryot Cell 5:696–711. doi:10.1128/EC.5.4.696-711.2006. 16607017 PMC1459671

[7] Branche C, Kohl L, Toutirais G, Buisson J, Cosson J, Bastin P. 2006. Conserved and specific functions of axoneme components in trypanosome motility. J Cell Sci 119:3443–3455. doi:10.1242/jcs.03078. 16882690

[8] Moreira-Leite FF, Sherwin T, Kohl L, Gull K. 2001. A trypanosome structure involved in transmitting cytoplasmic information during cell division. Science 294:610–612. doi:10.1126/science.1063775. 11641501

[9] Vickerman K. 1985. Developmental cycles and biology of pathogenic trypanosomes. Br Med Bull 41:105–114. doi:10.1093/oxfordjournals.bmb.a072036. 3928017

[10] Oberholzer M, Saada EA, Hill KL. 2015. Cyclic AMP regulates social behavior in African trypanosomes. mBio 6:e01954-14. doi:10.1128/mBio.01954-14. 25922395 PMC4436052

[11] Lopez MA, Saada EA, Hill KL. 2015. Insect stage-specific adenylate cyclases regulate social motility in African trypanosomes. Eukaryot Cell 14:104–112. doi:10.1128/EC.00217-14. 25416239 PMC4279026

[12] Shaw S, DeMarco SF, Rehmann R, Wenzler T, Florini F, Roditi I, Hill KL. 2019. Flagellar cAMP signaling controls trypanosome progression through host tissues. Nat Commun 10:803. doi:10.1038/s41467-019-08696-y. 30778051 PMC6379439

[13] Langousis G, Shimogawa MM, Saada EA, Vashisht AA, Spreafico R, Nager AR, Barshop WD, Nachury MV, Wohlschlegel JA, Hill KL. 2016. Loss of the BBSome perturbs endocytic trafficking and disrupts virulence of *Trypanosoma brucei*. Proc Natl Acad Sci U S A 113:632–637. doi:10.1073/pnas.1518079113. 26721397 PMC4725476

[14] Salmon D, Vanwalleghem G, Morias Y, Denoeud J, Krumbholz C, Lhomme F, Bachmaier S, Kador M, Gossmann J, Dias FB, De Muylder G, Uzureau P, Magez S, Moser M, De Baetselier P, Van Den Abbeele J, Beschin A, Boshart M, Pays E. 2012. Adenylate cyclases of *Trypanosoma brucei* Science 337:463–466. doi:10.1126/science.1222753. 22700656

[15] Gould MK, de Koning HP. 2011. Cyclic-nucleotide signalling in protozoa. FEMS Microbiol Rev 35:515–541. doi:10.1111/j.1574-6976.2010.00262.x. 21223322

[16] Gerdes JM, Davis EE, Katsanis N. 2009. The vertebrate primary cilium in development, homeostasis, and disease. Cell 137:32–45. doi:10.1016/j.cell.2009.03.023. 19345185 PMC3016012

[17] Vincensini L, Blisnick T, Bastin P. 2011. The importance of model organisms to study cilia and flagella biology. Biol Aujourdhui 205:5–28. doi:10.1051/jbio/2011005. 21501571

[18] Dean S, Moreira-Leite F, Varga V, Gull K. 2016. Cilium transition zone proteome reveals compartmentalization and differential dynamics of ciliopathy complexes. Proc Natl Acad Sci U S A 113:E5135–E5143. doi:10.1073/pnas.1604258113. 27519801 PMC5024643

[19] Field MC, Carrington M. 2009. The trypanosome flagellar pocket. Nat Rev Microbiol 7:775–786. doi:10.1038/nrmicro2221. 19806154

[20] Cachon J, Cachon M, Cosson M-P, Cosson J. 1988. The paraflagellar rod: a structure in search of a function. Biol Cell 63:169–181. doi:10.1016/0248-4900(88)90056-1.

[21] Sunter JD, Gull K. 2016. The flagellum attachment zone: 'the cellular ruler' of trypanosome morphology. Trends Parasitol 32:309–324. doi:10.1016/j.pt.2015.12.010. 26776656 PMC4827413

[22] Sun SY, Wang C, Yuan YA, He CY. 2013. An intracellular membrane junction consisting of flagellum adhesion glycoproteins links flagellum biogenesis to cell morphogenesis in *Trypanosoma brucei*. J Cell Sci 126:520–531. doi:10.1242/jcs.113621. 23178943

[23] Sharma AI, Olson CL, Engman DM. 2017. The lipid raft proteome of African trypanosomes contains many flagellar proteins. Pathogens 6:39. doi:10.3390/pathogens6030039. PMC5617996 28837104

[24] Sherwin T, Gull K. 1989. The cell division cycle of *Trypanosoma brucei brucei*: timing of event markers and cytoskeletal modulations. Philos Trans R Soc Lond B Biol Sci 323:573–588. doi:10.1098/rstb.1989.0037. 2568647

[25] Hughes LC, Ralston KS, Hill KL, Zhou ZH. 2012. Three-dimensional structure of the trypanosome flagellum suggests that the paraflagellar rod functions as a biomechanical spring. PLoS One 7:e25700. doi:10.1371/journal.pone.0025700. 22235240 PMC3250385

[26] Koyfman AY, Schmid MF, Gheiratmand L, Fu CJ, Khant HA, Huang D, He CY, Chiu W. 2011. Structure of *Trypanosoma brucei* flagellum accounts for its bihelical motion. Proc Natl Acad Sci U S A 108:11105–11108. doi:10.1073/pnas.1103634108. 21690369 PMC3131312

[27] Oberholzer M, Bregy P, Marti G, Minca M, Peier M, Seebeck T. 2007. Trypanosomes and mammalian sperm: one of a kind? Trends Parasitol 23:71–77. doi:10.1016/j.pt.2006.12.002. 17174157

[28] Portman N, Lacomble S, Thomas B, McKean PG, Gull K. 2009. Combining RNA interference mutants and comparative proteomics to identify protein components and dependences in a eukaryotic flagellum. J Biol Chem 284:5610–5619. doi:10.1074/jbc.M808859200. 19074134 PMC2645819

[29] Sunter JD, Benz C, Andre J, Whipple S, McKean PG, Gull K, Ginger ML, Lukes J. 2015. Modulation of flagellum attachment zone protein FLAM3 and regulation of the cell shape in *Trypanosoma brucei* life cycle transitions. J Cell Sci 128:3117–3130. doi:10.1242/jcs.171645. 26148511 PMC4541047

[30] Rotureau B, Van Den Abbeele J. 2013. Through the dark continent: African trypanosome development in the tsetse fly. Front Cell Infect Microbiol 3:53. doi:10.3389/fcimb.2013.00053. 24066283 PMC3776139

[31] Robinson DR, Sherwin T, Ploubidou A, Byard EH, Gull K. 1995. Microtubule polarity and dynamics in the control of organelle positioning, segregation, and cytokinesis in the trypanosome cell cycle. J Cell Biol 128:1163–1172. doi:10.1083/jcb.128.6.1163. 7896879 PMC2120423

[32] Saada EA, Kabututu ZP, Lopez M, Shimogawa MM, Langousis G, Oberholzer M, Riestra A, Jonsson ZO, Wohlschlegel JA, Hill KL. 2014. Insect stage-specific receptor adenylate cyclases are localized to distinct subdomains of the *Trypanosoma brucei* flagellar membrane. Eukaryot Cell 13:1064–1076. doi:10.1128/EC.00019-14. 24879126 PMC4135804

[33] Broadhead R, Dawe HR, Farr H, Griffiths S, Hart SR, Portman N, Shaw MK, Ginger ML, Gaskell SJ, McKean PG, Gull K. 2006. Flagellar motility is required for the viability of the bloodstream trypanosome. Nature 440:224–227. doi:10.1038/nature04541. 16525475

[34] Varga V, Moreira-Leite F, Portman N, Gull K. 2017. Protein diversity in discrete structures at the distal tip of the trypanosome flagellum. Proc Natl Acad Sci U S A 114:E6546–E6555. doi:10.1073/pnas.1703553114. 28724725 PMC5559017

[35] Oberholzer M, Langousis G, Nguyen HT, Saada EA, Shimogawa MM, Jonsson ZO, Nguyen SM, Wohlschlegel JA, Hill KL. 2011. Independent analysis of the flagellum surface and matrix proteomes provides insight into flagellum signaling in mammalian-infectious *Trypanosoma brucei*. Mol Cell Proteomics 10:M111.010538. doi:10.1074/mcp.M111.010538. PMC3205874 21685506

[36] Shimogawa MM, Saada EA, Vashisht AA, Barshop WD, Wohlschlegel JA, Hill KL. 2015. Cell surface proteomics provides insight into stage-specific remodeling of the host-parasite interface in *Trypanosoma brucei*. Mol Cell Proteomics 14:1977–1988. doi:10.1074/mcp.M114.045146. 25963835 PMC4587323

[37] Subota I, Julkowska D, Vincensini L, Reeg N, Buisson J, Blisnick T, Huet D, Perrot S, Santi-Rocca J, Duchateau M, Hourdel V, Rousselle JC, Cayet N, Namane A, Chamot-Rooke J, Bastin P. 2014. Proteomic analysis of intact flagella of procyclic *Trypanosoma brucei* cells identifies novel flagellar proteins with unique sub-localization and dynamics. Mol Cell Proteomics 13:1769–1786. doi:10.1074/mcp.M113.033357. 24741115 PMC4083114

[38] Lam SS, Martell JD, Kamer KJ, Deerinck TJ, Ellisman MH, Mootha VK, Ting AY. 2015. Directed evolution of APEX2 for electron microscopy and proximity labeling. Nat Methods 12:51–54. doi:10.1038/nmeth.3179. 25419960 PMC4296904

[39] Hung V, Udeshi ND, Lam SS, Loh KH, Cox KJ, Pedram K, Carr SA, Ting AY. 2016. Spatially resolved proteomic mapping in living cells with the engineered peroxidase APEX2. Nat Protoc 11:456–475. doi:10.1038/nprot.2016.018. 26866790 PMC4863649

[40] Vélez-Ramírez DE, Shimogawa MM, Ray S, Lopez A, Rayatpisheh S, Langousis G, Gallagher-Jones M, Dean S, Wohlschlegel JA, Hill KL. 2020. APEX2 proximity proteomics resolves flagellum subdomains and identifies flagellum tip-specific proteins in *Trypanosoma brucei*. bioRxiv doi:10.1101/2020.03.09.984815. PMC8141408 33568455

[41] Wirschell M, Olbrich H, Werner C, Tritschler D, Bower R, Sale WS, Loges NT, Pennekamp P, Lindberg S, Stenram U, Carlen B, Horak E, Kohler G, Nurnberg P, Nurnberg G, Porter ME, Omran H. 2013. The nexin-dynein regulatory complex subunit DRC1 is essential for motile cilia function in algae and humans. Nat Genet 45:262–268. doi:10.1038/ng.2533. 23354437 PMC3818796

[42] Oda T, Yanagisawa H, Kikkawa M. 2015. Detailed structural and biochemical characterization of the nexin-dynein regulatory complex. Mol Biol Cell 26:294–304. doi:10.1091/mbc.E14-09-1367. 25411337 PMC4294676

[43] Oberholzer M, Lopez MA, Ralston KS, Hill KL. 2009. Approaches for functional analysis of flagellar proteins in African trypanosomes. Methods Cell Biol 93:21–57. doi:10.1016/S0091-679X(08)93002-8. 20409810 PMC3821762

[44] Kabututu ZP, Thayer M, Melehani JH, Hill KL. 2010. CMF70 is a subunit of the dynein regulatory complex. J Cell Sci 123:3587–3595. doi:10.1242/jcs.073817. 20876659 PMC2951471

[45] Wirtz E, Leal S, Ochatt C, Cross GA. 1999. A tightly regulated inducible expression system for conditional gene knock-outs and dominant-negative genetics in *Trypanosoma brucei*. Mol Biochem Parasitol 99:89–101. doi:10.1016/s0166-6851(99)00002-x. 10215027

[46] Lacomble S, Portman N, Gull K. 2009. A protein-protein interaction map of the *Trypanosoma brucei* paraflagellar rod. PLoS One 4:e7685. doi:10.1371/journal.pone.0007685. 19888464 PMC2766642

[47] Thomas PD. 2017. The gene ontology and the meaning of biological function. Methods Mol Biol 1446:15–24. doi:10.1007/978-1-4939-3743-1_2. 27812932 PMC6438694

[48] Dean S, Sunter JD, Wheeler RJ. 2017. TrypTag.org: a trypanosome genome-wide protein localisation resource. Trends Parasitol 33:80–82. doi:10.1016/j.pt.2016.10.009. 27863903 PMC5270239

[49] Mick DU, Rodrigues RB, Leib RD, Adams CM, Chien AS, Gygi SP, Nachury MV. 2015. Proteomics of primary cilia by proximity labeling. Dev Cell 35:497–512. doi:10.1016/j.devcel.2015.10.015. 26585297 PMC4662609

[50] Nager AR, Goldstein JS, Herranz-Perez V, Portran D, Ye F, Garcia-Verdugo JM, Nachury MV. 2017. An actin network dispatches ciliary GPCRs into extracellular vesicles to modulate signaling. Cell 168:252.e14–263.e14. doi:10.1016/j.cell.2016.11.036. 28017328 PMC5235987

[51] He M, Agbu S, Anderson KV. 2017. Microtubule motors drive hedgehog signaling in primary cilia. Trends Cell Biol 27:110–125. doi:10.1016/j.tcb.2016.09.010. 27765513 PMC5258846

[52] Blaineau C, Tessier M, Dubessay P, Tasse L, Crobu L, Pages M, Bastien P. 2007. A novel microtubule-depolymerizing kinesin involved in length control of a eukaryotic flagellum. Curr Biol 17:778–782. doi:10.1016/j.cub.2007.03.048. 17433682

[53] Bertiaux E, Morga B, Blisnick T, Rotureau B, Bastin P. 2018. A grow-and-lock model for the control of flagellum length in trypanosomes. Curr Biol 28:3802.e3–3814.e3. doi:10.1016/j.cub.2018.10.031. 30449671

[54] Keeling J, Tsiokas L, Maskey D. 2016. Cellular mechanisms of ciliary length control. Cells 5:6. doi:10.3390/cells5010006. PMC4810091 26840332

[55] Fort C, Bastin P. 2014. Elongation of the axoneme and dynamics of intraflagellar transport. Med Sci (Paris) 30:955–961. doi:10.1051/medsci/20143011008. 25388576

[56] He M, Subramanian R, Bangs F, Omelchenko T, Liem KF, Jr, Kapoor TM, Anderson KV. 2014. The kinesin-4 protein Kif7 regulates mammalian Hedgehog signalling by organizing the cilium tip compartment. Nat Cell Biol 16:663–672. doi:10.1038/ncb2988. 24952464 PMC4085576

[57] Vickerman K. 1969. On the surface coat and flagellar adhesion in trypanosomes. J Cell Sci 5:163–193. 5353653 10.1242/jcs.5.1.163

[58] Savage AF, Kolev NG, Franklin JB, Vigneron A, Aksoy S, Tschudi C. 2016. Transcriptome profiling of *Trypanosoma brucei* development in the tsetse fly vector *Glossina morsitans*. PLoS One 11:e0168877. doi:10.1371/journal.pone.0168877. 28002435 PMC5176191

[59] Naguleswaran A, Doiron N, Roditi I. 2018. RNA-Seq analysis validates the use of culture-derived *Trypanosoma brucei* and provides new markers for mammalian and insect life-cycle stages. BMC Genomics 19:227. doi:10.1186/s12864-018-4600-6. 29606092 PMC5879877

[60] Absalon S, Blisnick T, Kohl L, Toutirais G, Dore G, Julkowska D, Tavenet A, Bastin P. 2008. Intraflagellar transport and functional analysis of genes required for flagellum formation in trypanosomes. Mol Biol Cell 19:929–944. doi:10.1091/mbc.e07-08-0749. 18094047 PMC2262991

[61] An T, Li Z. 2018. An orphan kinesin controls trypanosome morphology transitions by targeting FLAM3 to the flagellum. PLoS Pathog 14:e1007101. doi:10.1371/journal.ppat.1007101. 29813136 PMC5993322

[62] Liu W, Apagyi K, McLeavy L, Ersfeld K. 2010. Expression and cellular localisation of calpain-like proteins in *Trypanosoma brucei*. Mol Biochem Parasitol 169:20–26. doi:10.1016/j.molbiopara.2009.09.004. 19766148

[63] Varnaitė R, MacNeill SA. 2016. Meet the neighbors: mapping local protein interactomes by proximity-dependent labeling with BioID. Proteomics 16:2503–2518. doi:10.1002/pmic.201600123. 27329485 PMC5053326

[64] Hwang J, Espenshade PJ. 2016. Proximity-dependent biotin labelling in yeast using the engineered ascorbate peroxidase APEX2. Biochem J 473:2463–2469. doi:10.1042/BCJ20160106. 27274088 PMC5290329

[65] Roux KJ, Kim DI, Raida M, Burke B. 2012. A promiscuous biotin ligase fusion protein identifies proximal and interacting proteins in mammalian cells. J Cell Biol 196:801–810. doi:10.1083/jcb.201112098. 22412018 PMC3308701

[66] Morriswood B, Havlicek K, Demmel L, Yavuz S, Sealey-Cardona M, Vidilaseris K, Anrather D, Kostan J, Djinovic-Carugo K, Roux KJ, Warren G. 2013. Novel bilobe components in *Trypanosoma brucei* identified using proximity-dependent biotinylation. Eukaryot Cell 12:356–367. doi:10.1128/EC.00326-12. 23264645 PMC3571296

[67] An T, Zhou Q, Hu H, Cormaty H, Li Z. 2020. FAZ27 cooperates with FLAM3 and ClpGM6 to maintain cell morphology in *Trypanosoma brucei*. J Cell Sci 133:jcs245258. doi:10.1242/jcs.245258. 32393602 PMC7295586

[68] Hilton NA, Sladewski TE, Perry JA, Pataki Z, Sinclair-Davis AN, Muniz RS, Tran HL, Wurster JI, Seo J, de Graffenried CL. 2018. Identification of TOEFAZ1-interacting proteins reveals key regulators of *Trypanosoma brucei* cytokinesis. Mol Microbiol 109:306–326. doi:10.1111/mmi.13986. 29781112 PMC6359937

[69] Dang HQ, Zhou Q, Rowlett VW, Hu H, Lee KJ, Margolin W, Li Z. 2017. Proximity interactions among basal body components in *Trypanosoma brucei* identify novel regulators of basal body biogenesis and inheritance. mBio 8:e02120-16. doi:10.1128/mBio.02120-16. 28049148 PMC5210500

[70] Chen AL, Kim EW, Toh JY, Vashisht AA, Rashoff AQ, Van C, Huang AS, Moon AS, Bell HN, Bentolila LA, Wohlschlegel JA, Bradley PJ. 2015. Novel components of the toxoplasma inner membrane complex revealed by BioID. mBio 6:e02357-14. doi:10.1128/mBio.02357-14. 25691595 PMC4337574

[71] Rees JS, Li XW, Perrett S, Lilley KS, Jackson AP. 2015. Protein neighbors and proximity proteomics. Mol Cell Proteomics 14:2848–2856. doi:10.1074/mcp.R115.052902. 26355100 PMC4638030

[72] Branon TC, Bosch JA, Sanchez AD, Udeshi ND, Svinkina T, Carr SA, Feldman JL, Perrimon N, Ting AY. 2018. Efficient proximity labeling in living cells and organisms with TurboID. Nat Biotechnol 36:880–887. doi:10.1038/nbt.4201. 30125270 PMC6126969

[73] Martell JD, Deerinck TJ, Lam SS, Ellisman MH, Ting AY. 2017. Electron microscopy using the genetically encoded APEX2 tag in cultured mammalian cells. Nat Protoc 12:1792–1816. doi:10.1038/nprot.2017.065. 28796234 PMC5851282

[74] Mavylutov T, Chen X, Guo L, Yang J. 2018. APEX2- tagging of Sigma 1-receptor indicates subcellular protein topology with cytosolic N-terminus and ER luminal C-terminus. Protein Cell 9:733–737. doi:10.1007/s13238-017-0468-5. 28929457 PMC6053353

[75] Fisch C, Dupuis-Williams P. 2011. Ultrastructure of cilia and flagella - back to the future! Biol Cell 103:249–270. doi:10.1042/BC20100139. 21728999

[76] Edwards BFL, Wheeler RJ, Barker AR, Moreira-Leite FF, Gull K, Sunter JD. 2018. Direction of flagellum beat propagation is controlled by proximal/distal outer dynein arm asymmetry. Proc Natl Acad Sci U S A 115:E7341–E7350. doi:10.1073/pnas.1805827115. 30030284 PMC6077732

[77] Gould MK, Bachmaier S, Ali JA, Alsford S, Tagoe DN, Munday JC, Schnaufer AC, Horn D, Boshart M, de Koning HP. 2013. Cyclic AMP effectors in African trypanosomes revealed by genome-scale RNA interference library screening for resistance to the phosphodiesterase inhibitor CpdA. Antimicrob Agents Chemother 57:4882–4893. doi:10.1128/AAC.00508-13. 23877697 PMC3811416

[78] Tompa P, Emori Y, Sorimachi H, Suzuki K, Friedrich P. 2001. Domain III of calpain is a Ca^2+^-regulated phospholipid-binding domain. Biochem Biophys Res Commun 280:1333–1339. doi:10.1006/bbrc.2001.4279. 11162675

[79] Aslett M, Aurrecoechea C, Berriman M, Brestelli J, Brunk BP, Carrington M, Depledge DP, Fischer S, Gajria B, Gao X, Gardner MJ, Gingle A, Grant G, Harb OS, Heiges M, Hertz-Fowler C, Houston R, Innamorato F, Iodice J, Kissinger JC, Kraemer E, Li W, Logan FJ, Miller JA, Mitra S, Myler PJ, Nayak V, Pennington C, Phan I, Pinney DF, Ramasamy G, Rogers MB, Roos DS, Ross C, Sivam D, Smith DF, Srinivasamoorthy G, Stoeckert CJ, Jr, Subramanian S, Thibodeau R, Tivey A, Treatman C, Velarde G, Wang H. 2010. TriTrypDB: a functional genomic resource for the *Trypanosomatidae*. Nucleic Acids Res 38:D457–D462. doi:10.1093/nar/gkp851. 19843604 PMC2808979

[80] Briggs LJ, McKean PG, Baines A, Moreira-Leite F, Davidge J, Vaughan S, Gull K. 2004. The flagella connector of *Trypanosoma brucei*: an unusual mobile transmembrane junction. J Cell Sci 117:1641–1651. doi:10.1242/jcs.00995. 15075226

[81] Fort C, Bonnefoy S, Kohl L, Bastin P. 2016. Intraflagellar transport is required for the maintenance of the trypanosome flagellum composition but not its length. J Cell Sci 129:3026–3041. doi:10.1242/jcs.188227. 27343245

[82] Douglas RL, Haltiwanger BM, Albisetti A, Wu H, Jeng RL, Mancuso J, Cande WZ, Welch MD. 2020. Trypanosomes have divergent kinesin-2 proteins that function differentially in flagellum biosynthesis and cell viability. J Cell Sci 133:jcs129213. doi:10.1242/jcs.129213. 32503938

[83] Chien A, Shih SM, Bower R, Tritschler D, Porter ME, Yildiz A. 2017. Dynamics of the IFT machinery at the ciliary tip. Elife 6:e28606. doi:10.7554/eLife.28606. 28930071 PMC5662288

[84] Prevo B, Mangeol P, Oswald F, Scholey JM, Peterman EJ. 2015. Functional differentiation of cooperating kinesin-2 motors orchestrates cargo import and transport in *C. elegans* cilia. Nat Cell Biol 17:1536–1545. doi:10.1038/ncb3263. 26523365

[85] Del Viso F, Huang F, Myers J, Chalfant M, Zhang Y, Reza N, Bewersdorf J, Lusk CP, Khokha MK. 2016. Congenital heart disease genetics uncovers context-dependent organization and function of nucleoporins at cilia. Dev Cell 38:478–492. doi:10.1016/j.devcel.2016.08.002. 27593162 PMC5021619

[86] Blisnick T, Buisson J, Absalon S, Marie A, Cayet N, Bastin P. 2014. The intraflagellar transport dynein complex of trypanosomes is made of a heterodimer of dynein heavy chains and of light and intermediate chains of distinct functions. Mol Biol Cell 25:2620–2633. doi:10.1091/mbc.E14-05-0961. 24989795 PMC4148251

[87] Wren KN, Craft JM, Tritschler D, Schauer A, Patel DK, Smith EF, Porter ME, Kner P, Lechtreck KF. 2013. A differential cargo-loading model of ciliary length regulation by IFT. Curr Biol 23:2463–2471. doi:10.1016/j.cub.2013.10.044. 24316207 PMC3881561

[88] Jortzik E, Becker K. 2012. Thioredoxin and glutathione systems in *Plasmodium falciparum*. Int J Med Microbiol 302:187–194. doi:10.1016/j.ijmm.2012.07.007. 22939033

[89] Oberholzer M, Morand S, Kunz S, Seebeck T. 2006. A vector series for rapid PCR-mediated C-terminal *in situ* tagging of *Trypanosoma brucei* genes. Mol Biochem Parasitol 145:117–120. doi:10.1016/j.molbiopara.2005.09.002. 16269191

[90] Cock PJA, Antao T, Chang JT, Chapman BA, Cox CJ, Dalke A, Friedberg I, Hamelryck T, Kauff F, Wilczynski B, de Hoon MJL. 2009. Biopython: freely available Python tools for computational molecular biology and bioinformatics. Bioinformatics 25:1422–1423. doi:10.1093/bioinformatics/btp163. 19304878 PMC2682512

[91] Lopez MA. 2013. Investigation of mechanisms underlying African trypanosome social behavior. Thesis. University of California, Los Angeles, Los Angeles, CA.

[92] Demonchy R, Blisnick T, Deprez C, Toutirais G, Loussert C, Marande W, Grellier P, Bastin P, Kohl L. 2009. Kinesin 9 family members perform separate functions in the trypanosome flagellum. J Cell Biol 187:615–622. doi:10.1083/jcb.200903139. 19948486 PMC2806587

