# Supplementary figures and images for: APEX2 Proximity Proteomics Resolves Flagellum Subdomains and Identifies Flagellum Tip-Specific Proteins in Trypanosoma brucei
Source: mSphere. 2021 Feb 10;6(1):e01090-20. doi: 10.1128/mSphere.01090-20 (PMC8141408; doi:10.1128/mSphere.01090-20)

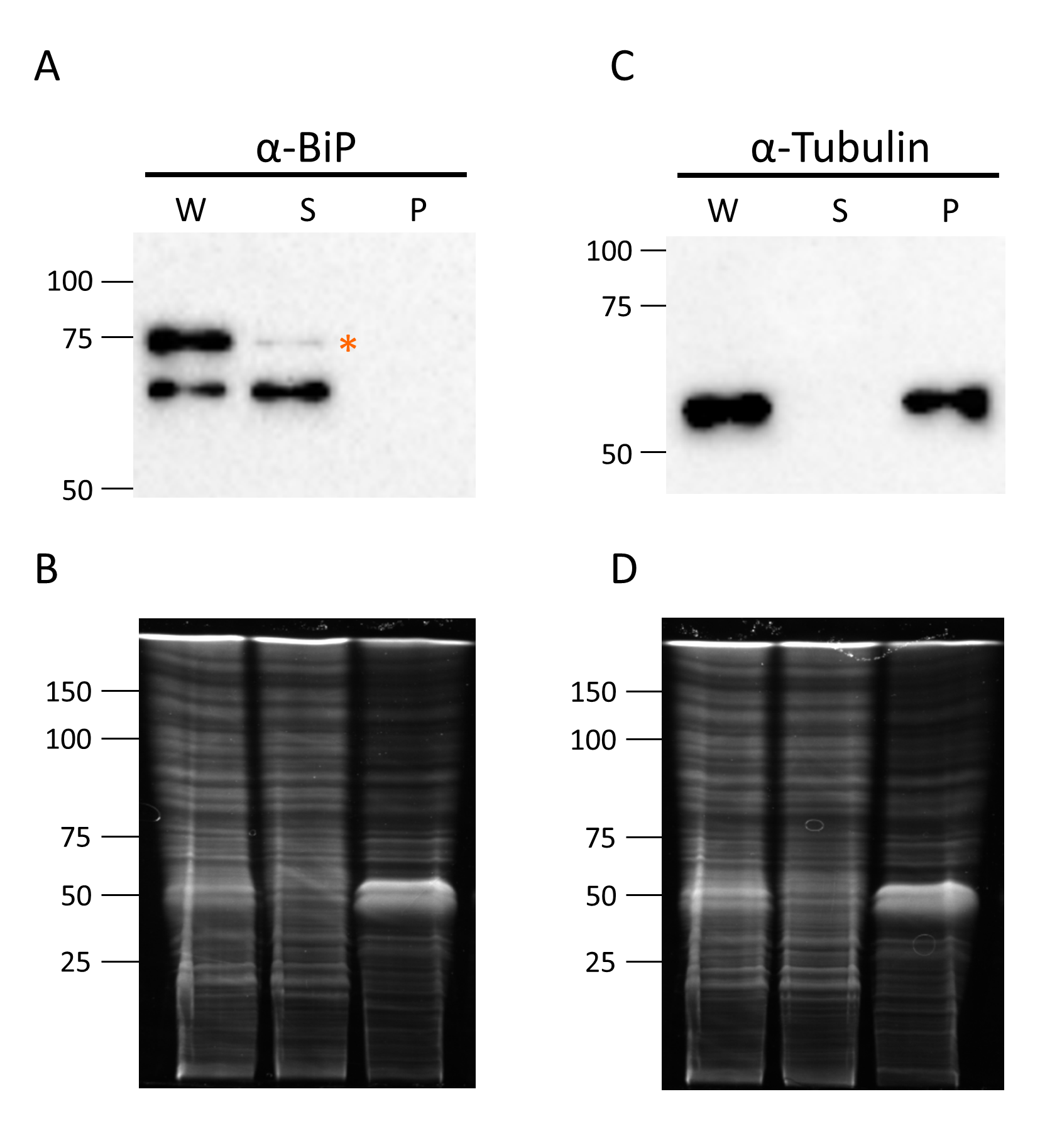

Supplement: FIG S1 [file mSphere.01090-20-sf001.tif]

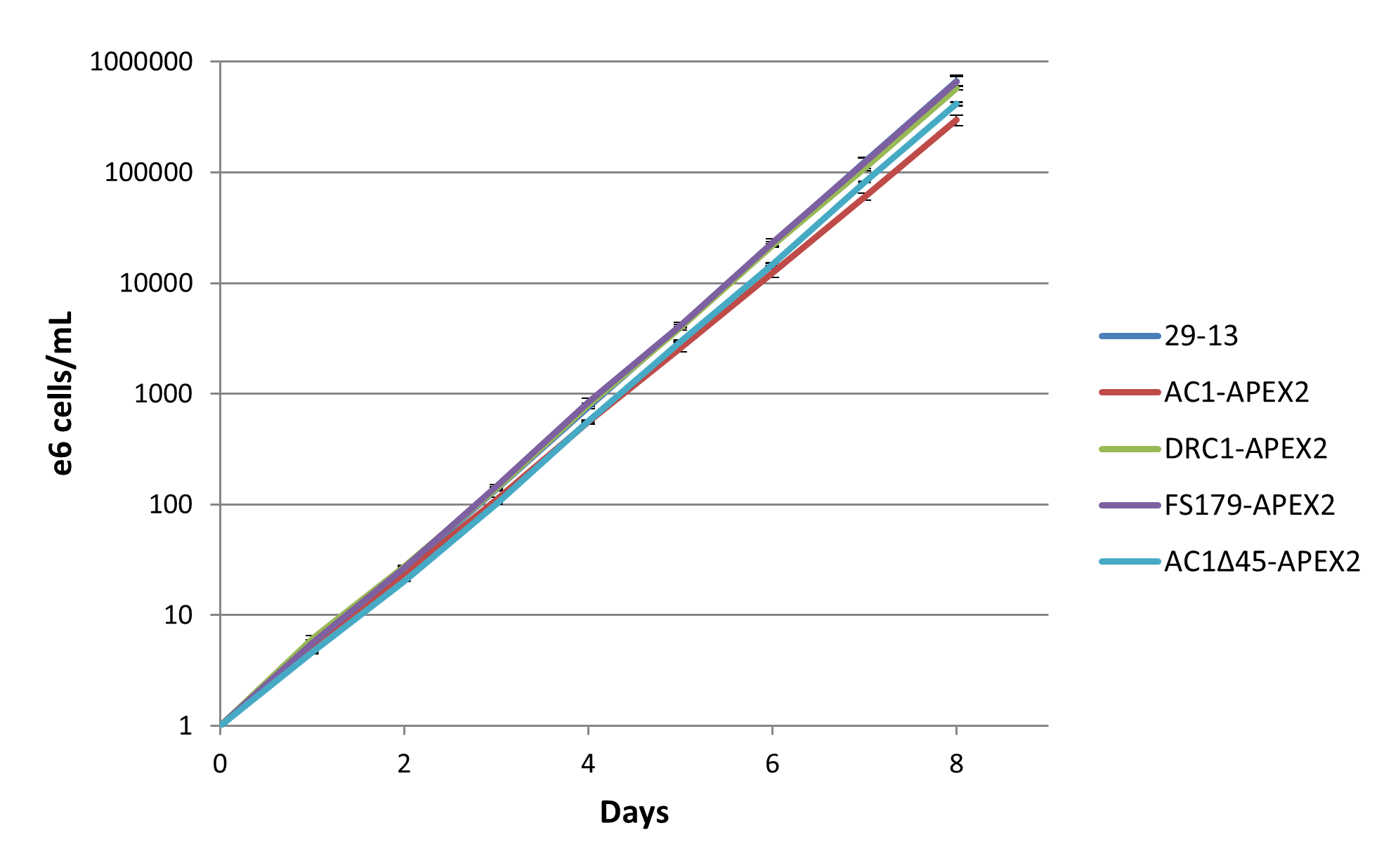

Supplement: FIG S2 [file mSphere.01090-20-sf002.tif]

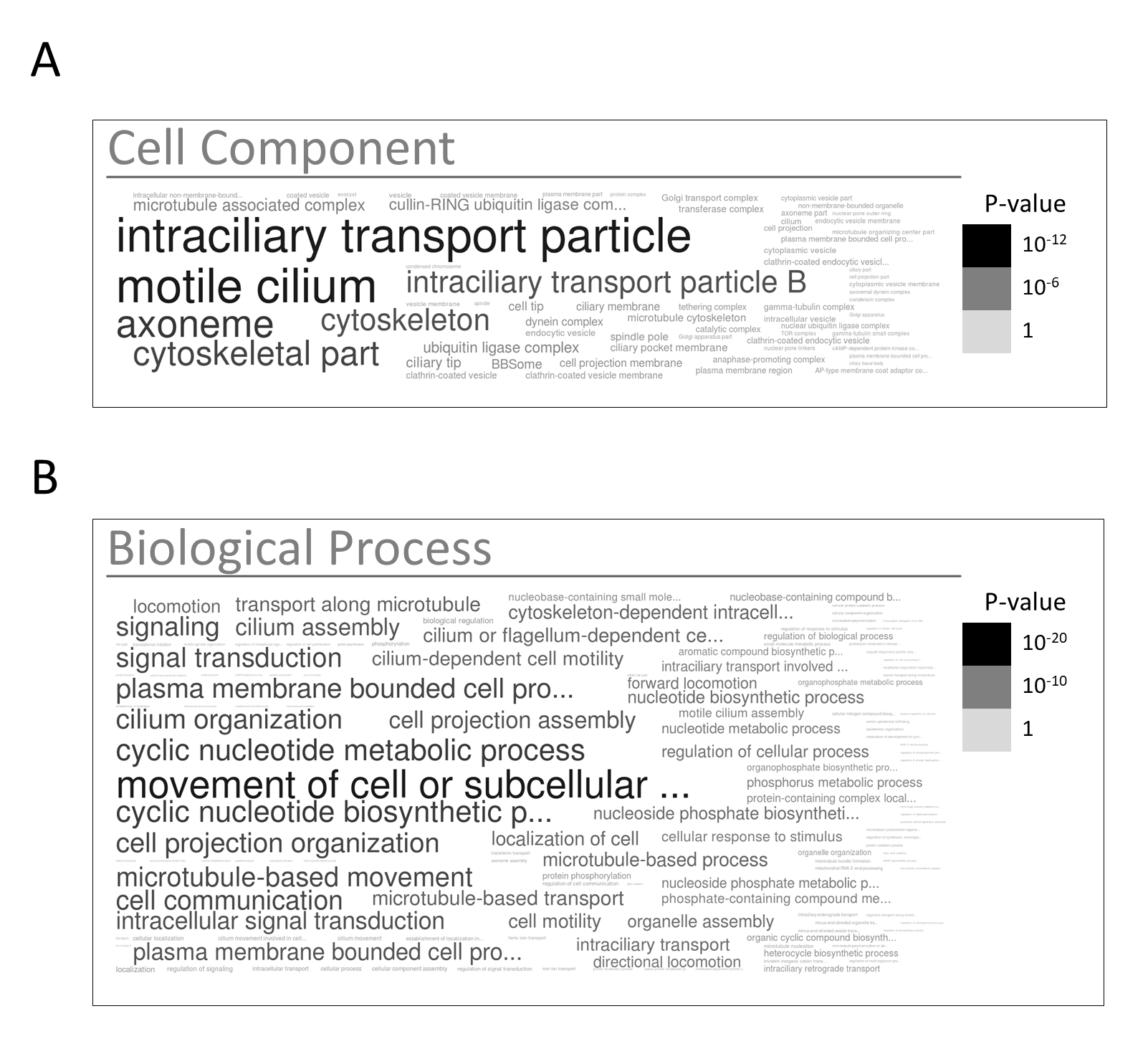

Supplement: FIG S3 [file mSphere.01090-20-sf003.tif]

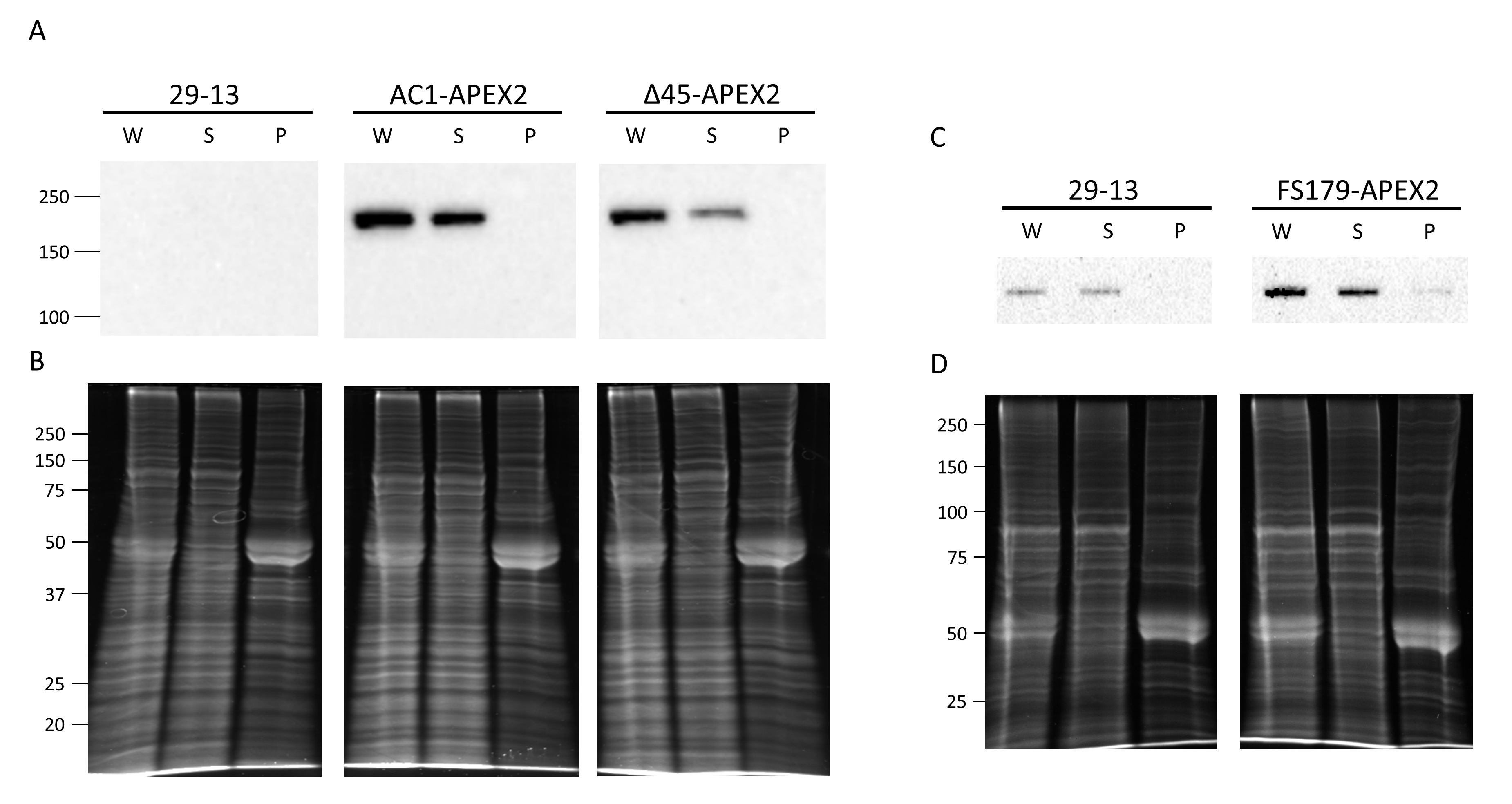

Supplement: FIG S4 [file mSphere.01090-20-sf004.tif]

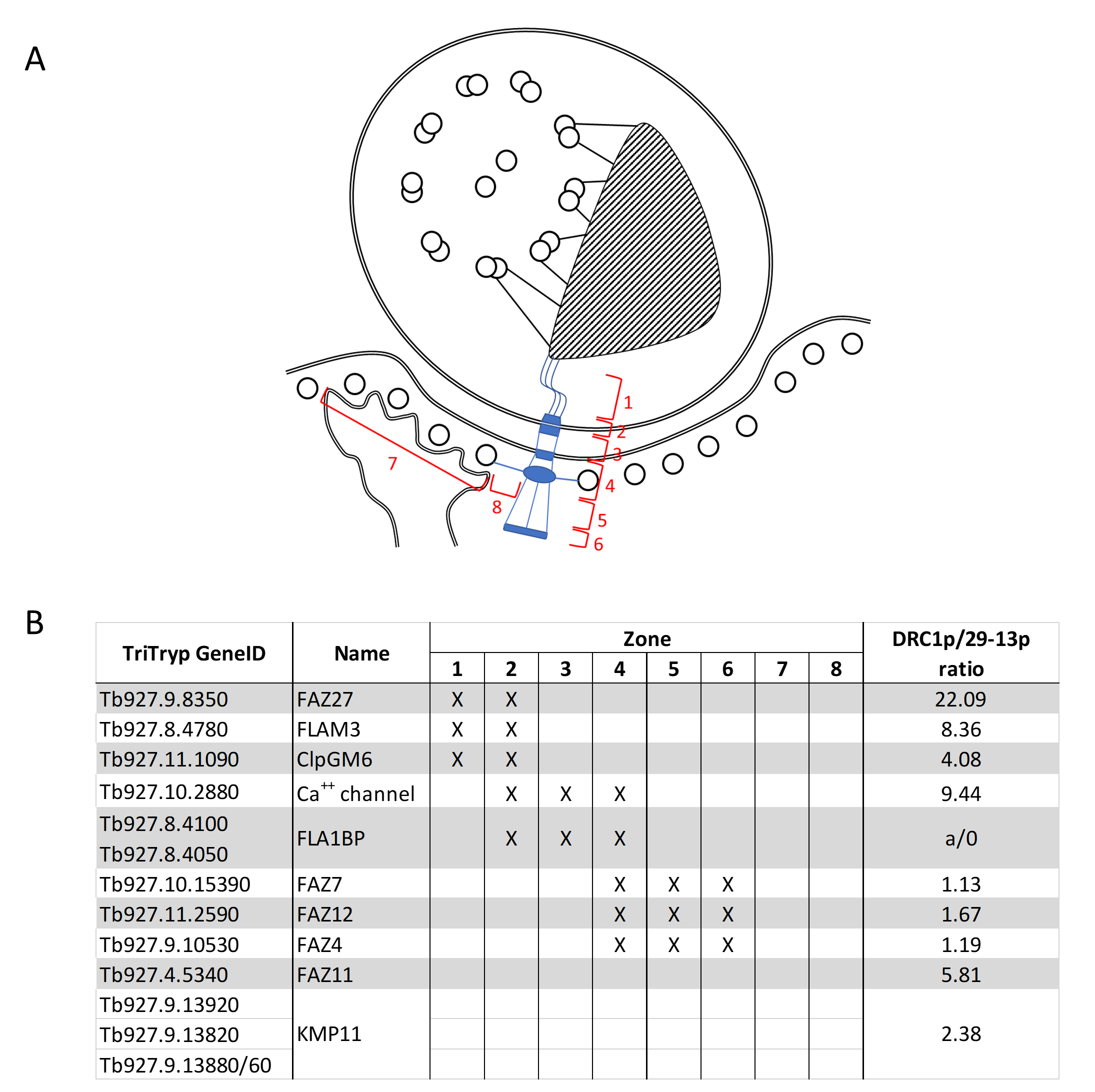

Supplement: FIG S5 [file mSphere.01090-20-sf005.tif]
