# Supplementary material for: APEX2 Proximity Proteomics Resolves Flagellum Subdomains and Identifies Flagellum Tip-Specific Proteins in Trypanosoma brucei
Source: mSphere. 2021 Feb 10;6(1):e01090-20. doi: 10.1128/mSphere.01090-20 (PMC8141408; doi:10.1128/mSphere.01090-20)
Supplement: TABLE S1 [file mSphere.01090-20-st001.pdf]

Supplemental Table S1A. Proteins in the DRC1p proximity proteome that were not identified in prior flagellum proteome studies.

Accession	Description	Accession	Description
Tb927.1.1010	E3 ubiquitin-protein ligase KCMF1, putative	Tb927.3.2760	hypothetical protein, conserved
Tb927.1.1100	Basal body protein	Tb927.3.2830	brix domain containing protein, putative
Tb927.1.1840	conserved protein, unknown function	Tb927.3.2950	ribonuclease inhibitor- like protein
Tb927.1.2640	hypothetical protein, conserved	Tb927.3.3120	hypothetical protein, conserved
Tb927.1.3250	Domain of unknown function (DUF4456), putative	Tb927.3.3350	hypothetical protein, conserved
Tb927.1.4300	chaperone protein DnaJ, putative	Tb927.3.3650	hypothetical protein, conserved
Tb927.1.4420	ABC transporter, putative	Tb927.3.4020	phosphatidylinositol 4-kinase alpha, putative
Tb927.1.690	hypothetical protein, conserved	Tb927.3.4510	hypothetical protein, conserved
Tb927.1.780	leucine-rich repeat-containing protein	Tb927.3.4610	Right handed beta helix region, putative
Tb927.10.10090	Component of motile flagella 14	Tb927.3.4790	Aminopeptidase M1, putative
Tb927.10.11070	hypothetical protein, conserved	Tb927.3.5040	hypothetical protein, conserved
Tb927.10.1120	hypothetical protein, conserved	Tb927.3.5110	hypothetical protein, conserved
Tb927.10.1150	hypothetical protein, conserved	Tb927.3.5120	Ankyrin repeats (3 copies), putative
Tb927.10.11550	hypothetical protein, conserved	Tb927.3.5160	Basal body protein
Tb927.10.12490	kinesin, putative	Tb927.3.5260	hypothetical protein, conserved
Tb927.10.12870	Basal body protein	Tb927.3.690	CMGC/RCK protein kinase, putative
Tb927.10.13000	cAMP phosphodiesterase A, putative	Tb927.3.730	hypothetical protein, conserved
Tb927.10.13030	cysteine peptidase, Clan CA, family C2, putative	Tb927.3.920	hypothetical protein, conserved
Tb927.10.13150	hypothetical protein, conserved	Tb927.4.1150	hypothetical protein, conserved
Tb927.10.13610	zinc-finger of a C2HC-type/Double zinc ribbon, putative	Tb927.4.1430	hypothetical protein, conserved
Tb927.10.13870	tubulin tyrosine ligase protein, putative	Tb927.4.1750	hypothetical protein, conserved
Tb927.10.14220	hypothetical protein, conserved	Tb927.4.2240	Aminotransferase class IV, putative
Tb927.10.14230	hypothetical protein, conserved	Tb927.4.2640	hypothetical protein, conserved
Tb927.10.14300	MEKK-related kinase 1, putative	Tb927.4.2730	kinesin, putative
Tb927.10.14420	Serine/threonine-protein kinase NEK16, putative	Tb927.4.2880	Nucleoporin NUP225
Tb927.10.14480	hypothetical protein, conserved	Tb927.4.2990	hypothetical protein, conserved
Tb927.10.14730	chaperone protein DnaJ, putative	Tb927.4.3470	hypothetical protein, conserved
Tb927.10.1510	CCR4-NOT transcription complex subunit 1	Tb927.4.3770	calcium/calmodulin-dependent protein kinase, putative
Tb927.10.15230	hypothetical protein, conserved	Tb927.4.3810	DNA-directed RNA polymerase II subunit 2, putative
Tb927.10.15450	hypothetical protein, conserved	Tb927.4.3890	ATP-dependent RNA helicase, putative
Tb927.10.15550	hypothetical protein, conserved	Tb927.4.4400	hypothetical protein, conserved
Tb927.10.15660	hypothetical protein, conserved	Tb927.4.850	hypothetical protein, conserved
Tb927.10.15680	hypothetical protein, conserved	Tb927.5.1010	hypothetical protein, conserved
Tb927.10.15730	SPRY domain/Ankyrin repeats (3 copies), putative	Tb927.5.1140	choline/ethanolamine kinase
Tb927.10.1630	atp-binding cassette sub-family e member 1	Tb927.5.1540	hypothetical protein, conserved
Tb927.10.1980	hypothetical protein, conserved	Tb927.5.1920	hypothetical protein, conserved
Tb927.10.2650	hypothetical protein, conserved	Tb927.5.2410	kinesin, putative
Tb927.10.2900	importin beta-1 subunit, putative	Tb927.5.2620	hypothetical protein, conserved
Tb927.10.2920	SUMO-interacting motif-containing protein	Tb927.5.2680	hypothetical protein, conserved
Tb927.10.3010	hook complex protein, conserved	Tb927.5.2890	AAA domain (dynein-related subfamily)/von Willebrand factor type A domain containing protein, putative
Tb927.10.3780	hypothetical protein, conserved	Tb927.5.3230	hypothetical protein, conserved
Tb927.10.3790	hypothetical protein, conserved	Tb927.5.3290	Protein of unknown function, putative
Tb927.10.3870	hypothetical protein, conserved	Tb927.5.3330	hypothetical protein, conserved
Tb927.10.4290	hypothetical protein, conserved	Tb927.5.3650	hypothetical protein, conserved
Tb927.10.4320	hypothetical protein, conserved	Tb927.5.4110	hypothetical protein, conserved

Tb927.10.4600	predicted SET domain protein	Tb927.5.4380	Kinetoplastid-specific Protein Phosphatase 1
Tb927.10.4750	hypothetical protein, conserved	Tb927.5.4400	hypothetical protein, conserved
Tb927.10.4890	zinc-finger of a C2HC-type, putative	Tb927.5.570	hypothetical protein, conserved
Tb927.10.4920	hypothetical protein, conserved	Tb927.5.950	monothiol glutaredoxin, putative
Tb927.10.4940	Nuclear Dbf2-related kinase	Tb927.6.1480	hypothetical protein, conserved
Tb927.10.5000	hypothetical protein, conserved	Tb927.6.2120	hypothetical protein, conserved
Tb927.10.5170	XPC-binding domain containing protein, putative	Tb927.6.2690	ubiquitin carboxyl-terminal hydrolase, putative
Tb927.10.5240	cAMP binding protein, putative	Tb927.6.2760	hypothetical protein, conserved
Tb927.10.5380	intraflagellar transport protein 122	Tb927.6.2850	ESAG8-associated protein, putative
Tb927.10.5460	60S ribosomal protein L24, putative	Tb927.6.3020	hypothetical protein, conserved
Tb927.10.5480	60S ribosomal protein L24, putative	Tb927.6.3100	Intraflagellar transport complex B protein 46 C terminal, putative
Tb927.10.5580	hypothetical protein, conserved	Tb927.6.3310	cysteine peptidase, Clan CA, family C2, putative
Tb927.10.5890	Galactose oxidase, central domain containing protein, putative	Tb927.6.4640	hypothetical protein, conserved
Tb927.10.5930	NEK family Serine/threonine-protein kinase, putative	Tb927.6.4750	hypothetical protein, conserved
Tb927.10.6000	hypothetical protein, conserved	Tb927.7.1230	hypothetical protein, conserved
Tb927.10.6110	hypothetical protein, conserved	Tb927.7.1240	Sphingosine kinase
Tb927.10.6210	TPR repeat, putative	Tb927.7.1560	TPR repeat, putative
Tb927.10.6230	MORN repeat containing protein	Tb927.7.1760	hypothetical protein, conserved
Tb927.10.6330	kinetoplastid kinetochore protein 1	Tb927.7.1870	hypothetical protein, conserved
Tb927.10.6340	hypothetical protein, conserved	Tb927.7.2280	hypothetical protein, conserved
Tb927.10.6580	hypothetical protein	Tb927.7.2350	hypothetical protein, conserved
Tb927.10.6980	caltractin, putative	Tb927.7.2730	leucine-rich repeat protein (LRRP), putative
Tb927.10.7830	hypothetical protein, conserved	Tb927.7.2810	hypothetical protein, conserved
Tb927.10.820	hypothetical protein, conserved	Tb927.7.290	leucine-rich repeat protein 1 (LRRP1), putative
Tb927.10.8390	Concanavalin A-like lectin/glucanases superfamily/SPRY domain/HECT-domain (ubiquitin-transferase), putative	Tb927.7.3000	kinesin, putative
Tb927.10.8910	Nucleoporin NUP181	Tb927.7.3130	Transition Zone Protein 103 kDa
Tb927.10.930	hypothetical protein, conserved	Tb927.7.3400	MORN repeat, putative
Tb927.10.9380	SEP domain/UBX domain containing protein, putative	Tb927.7.3540	hypothetical protein, conserved
Tb927.10.970	Tetratricopeptide repeat, putative	Tb927.7.3580	Serine/threonine-protein kinase NEK11, putative
Tb927.11.10120	hypothetical protein, conserved	Tb927.7.3610	hypothetical protein, conserved
Tb927.11.10210	hypothetical protein, conserved	Tb927.7.3670	TrkA-N domain containing protein, putative
Tb927.11.1030	kinetoplastid kinetochore protein 7	Tb927.7.3880	Basal body protein
Tb927.11.10350	hypothetical protein, conserved	Tb927.7.4130	hypothetical protein, conserved
Tb927.11.10360	hypothetical protein, conserved	Tb927.7.4360	hypothetical protein, conserved
Tb927.11.10520	kinetoplastid kinetochore protein 2	Tb927.7.4370	hypothetical protein, conserved
Tb927.11.10560	eukaryotic translation initiation factor 4 gamma 4	Tb927.7.4410	hypothetical protein, conserved
Tb927.11.10610	hypothetical protein, conserved	Tb927.7.4600	Calponin homology (CH) domain containing protein, putative
Tb927.11.10660	Basal body protein	Tb927.7.4900	5'-3' exoribonuclease A
Tb927.11.11090	Nucleoporin NUP140	Tb927.7.500	hypothetical protein, conserved
Tb927.11.1110	calpain, putative	Tb927.7.510	hypothetical protein, conserved
Tb927.11.11260	hypothetical protein, conserved	Tb927.7.5410	hypothetical protein, conserved
Tb927.11.1130	calpain-like cysteine peptidase, putative	Tb927.7.5430	hypothetical protein, conserved
Tb927.11.11600	hypothetical protein, conserved	Tb927.7.6220	calcium/calmodulin-dependent protein kinase, putative
Tb927.11.11880	conserved protein	Tb927.7.6310	polo-like protein kinase
Tb927.11.1220	hypothetical protein, conserved	Tb927.7.6680	STE/STE11 serine/threonine-protein kinase, putative
Tb927.11.12430	cell division cycle phosphatase 14, putative	Tb927.7.6760	hypothetical protein, conserved

Tb927.11.12510	hypothetical protein, conserved	Tb927.7.7000	hook complex protein, conserved
Tb927.11.12970	hypothetical protein, conserved	Tb927.7.7070	MYND finger, putative
Tb927.11.13360	AAA ATPase, putative	Tb927.7.7100	hypothetical protein
Tb927.11.13450	hypothetical protein, conserved	Tb927.7.7190	hypothetical protein, conserved
Tb927.11.13490	hypothetical protein, conserved	Tb927.7.7260	kinesin, putative
Tb927.11.1380	Right handed beta helix region/Periplasmic copper-binding protein (NosD), putative	Tb927.7.7320	hypothetical protein, conserved
Tb927.11.13840	hypothetical protein, conserved	Tb927.7.7400	Basal body protein
Tb927.11.13920	kinesin-II 85 Kd subunit, putative	Tb927.7.860	hypothetical protein, conserved
Tb927.11.14220	Regulator of ESAG9-1	Tb927.8.1080	centrin, putative
Tb927.11.14410	Ankyrin repeats (3 copies), putative	Tb927.8.1280	hypothetical protein, conserved
Tb927.11.14680	phosphatidylinositol 3-related kinase, putative	Tb927.8.1360	Dynein heavy chain, N-terminal region 2, putative
Tb927.11.14920	hypothetical protein, conserved	Tb927.8.1890	cytochrome c1, heme protein, mitochondrial, putative
Tb927.11.15250	hypothetical protein, conserved	Tb927.8.2220	hypothetical protein, conserved
Tb927.11.15310	hypothetical protein, conserved	Tb927.8.2440	hypothetical protein, conserved
Tb927.11.15450	Basal body protein	Tb927.8.2590	Basal body protein
Tb927.11.15610	hypothetical protein, conserved	Tb927.8.2660	Galactose oxidase, central domain containing protein, putative
Tb927.11.15740	hypothetical protein, conserved	Tb927.8.2870	conserved protein
Tb927.11.15750	AMP deaminase, putative	Tb927.8.3000	hypothetical protein, conserved
Tb927.11.15940	hypothetical protein, conserved	Tb927.8.3010	hook complex protein, conserved
Tb927.11.16210	cAMP response protein 1, putative	Tb927.8.3410	Inositol hexakisphosphate, putative
Tb927.11.16790	mitogen-activated protein kinase	Tb927.8.3590	hypothetical protein, conserved
Tb927.11.2290	hypothetical protein, conserved	Tb927.8.3700	hypothetical protein, conserved
Tb927.11.2590	Flagellum attachment zone protein 12	Tb927.8.3990	hypothetical protein, conserved
Tb927.11.3110	hypothetical protein, conserved	Tb927.8.4100	FLA1-binding protein
Tb927.11.3140	dual specificity tyrosine-phosphorylation-regulated kinase 2, putative	Tb927.8.4160	hypothetical protein, conserved
Tb927.11.3390	Concanavalin A-like lectin/glucanases superfamily/SPRY domain/HECT-domain (ubiquitin-transferase), putative	Tb927.8.4320	hypothetical protein, conserved
Tb927.11.3680	calmodulin, putative	Tb927.8.4480	hypothetical protein, conserved
Tb927.11.3910	hypothetical protein, conserved	Tb927.8.4510	Nucleoside diphosphate kinase 4
Tb927.11.3990	hypothetical protein, conserved	Tb927.8.4590	hypothetical protein, conserved
Tb927.11.4010	hypothetical protein, conserved	Tb927.8.4910	hypothetical protein, conserved
Tb927.11.4210	U-box domain containing protein, putative	Tb927.8.5020	hypothetical protein, conserved
Tb927.11.4280	hypothetical protein, conserved	Tb927.8.5110	hypothetical protein, conserved
Tb927.11.4350	SUMO-interacting motif-containing protein	Tb927.8.5170	hypothetical protein, conserved
Tb927.11.4540	Nucleoporin NUP48	Tb927.8.5290	WW domain containing protein, putative
Tb927.11.4660	Kinesin-13 2	Tb927.8.5940	hypothetical protein, conserved
Tb927.11.4720	hypothetical protein, conserved	Tb927.8.5970	Cullin 5, putative
Tb927.11.4740	paraflagellar rod component, putative	Tb927.8.6250	Nucleoporin NUP76
Tb927.11.4950	hypothetical protein, conserved	Tb927.8.6490	Basal body protein
Tb927.11.5040	leucine-rich repeat protein (LRRP), putative	Tb927.8.6650	RNA-binding protein, putative
Tb927.11.5190	leucine-rich repeat protein (LRRP), putative	Tb927.8.6790	hypothetical protein, conserved
Tb927.11.5250	hypothetical protein, conserved	Tb927.8.6830	kinesin, putative
Tb927.11.560	hypothetical protein, conserved	Tb927.8.6870	hypothetical protein, conserved
Tb927.11.5660	hypothetical protein, conserved	Tb927.8.6940	hypothetical protein, conserved
Tb927.11.5770	hypothetical protein, conserved	Tb927.8.7060	hypothetical protein, conserved
Tb927.11.630	RNA polymerase I second largest subunit	Tb927.8.7080	hypothetical protein, conserved
Tb927.11.6350	AAA ATPase, putative	Tb927.8.790	hypothetical protein, conserved
Tb927.11.6430	hypothetical protein, conserved	Tb927.8.7970	hypothetical protein
Tb927.11.6780	regulator of chromatin condensation, putative	Tb927.8.8070	conserved protein
Tb927.11.6820	hypothetical protein, conserved	Tb927.8.960	hypothetical protein, conserved
Tb927.11.7180	MORN repeat, putative	Tb927.9.10530	Flagellum attachment zone protein 4

Tb927.11.7670	Cornifin (SPRR) family, putative
Tb927.11.8210	RAD50 DNA repair-like protein
Tb927.11.8290	DIS3-like exonuclease, putative
Tb927.11.8300	hypothetical protein, conserved
Tb927.11.8410	hypothetical protein, conserved
Tb927.11.910	hypothetical protein, conserved
Tb927.11.9220	proteasome regulatory non-ATP-ase subunit 2
Tb927.11.9240	hypothetical protein, conserved
Tb927.11.9370	hypothetical protein, conserved
Tb927.11.9650	hypothetical protein, conserved
Tb927.11.9680	hypothetical protein, conserved
Tb927.11.9780	Nucleoporin NUP119
Tb927.11.9880	Protein of unknown function (DUF2009), putative
Tb927.2.2090	SUMO-interacting motif-containing protein
Tb927.2.2880	TPR repeat/Tetratricopeptide repeat, putative
Tb927.2.3000	hypothetical protein, conserved
Tb927.2.3730	hypothetical protein, conserved
Tb927.2.4160	hypothetical protein, conserved
Tb927.2.4200	CMGC/CLK family protein kinase, putative
Tb927.2.4480	hypothetical protein, conserved
Tb927.2.4780	hypothetical protein, conserved
Tb927.2.4930	esterase, putative
Tb927.2.5010	Meiotic cell cortex C-terminal pleckstrin homology, putative
Tb927.2.5220	hypothetical protein, conserved
Tb927.2.5530	Present in the outer mitochondrial membrane proteome 22-1
Tb927.2.5610	Present in the outer mitochondrial membrane proteome 22B
Tb927.2.5860	hypothetical protein, conserved
Tb927.2.5870	hypothetical protein, conserved
Tb927.2.5950	hypothetical protein, conserved
Tb927.3.1190	hypothetical protein, conserved
Tb927.3.2040	kinesin, putative
Tb927.3.2110	Mitochondrial import inner membrane translocase subunit TIM50
Tb927.3.2440	AGC essential kinase 1
Tb927.3.2640	hypothetical protein, conserved

Tb927.9.10950	chaperone protein DnaJ, putative
Tb927.9.12490	hypothetical protein, conserved
Tb927.9.12800	hypothetical protein, conserved
Tb927.9.13300	Ring finger domain containing protein, putative
Tb927.9.13610	helicase, putative
Tb927.9.14220	metacaspase 5, putative
Tb927.9.15400	ankyrin-repeat protein, putative
Tb927.9.1770	Zn-finger in Ran binding protein and others/SPRY domain/HECT-domain (ubiquitin-transferase), putative
Tb927.9.2130	hypothetical protein, conserved
Tb927.9.2900	U3 small nucleolar RNA-associated protein 10, putative
Tb927.9.3030	hypothetical protein, conserved
Tb927.9.3120	STE/STE11 serine/threonine-protein kinase, putative
Tb927.9.3130	hypothetical protein, conserved
Tb927.9.3400	endo-beta-N-acetylglucosaminidase, putative
Tb927.9.3680	hypothetical protein, conserved
Tb927.9.3870	hypothetical protein, conserved
Tb927.9.4420	Beat-regulation protein
Tb927.9.5520	ubiquitin carboxyl-terminal hydrolase, putative
Tb927.9.5620	hypothetical protein, conserved
Tb927.9.6350	inositol-1(or 4)-monophosphatase, putative
Tb927.9.6460	hypothetical protein, conserved
Tb927.9.6880	cAMP-specific phosphodiesterase, putative
Tb927.9.7690	SUMO-interacting motif-containing protein
Tb927.9.8330	hypothetical protein
Tb927.9.8350	hypothetical protein, conserved
Tb927.9.8500	hypothetical protein
Tb927.9.9300	hypothetical protein, conserved
Tb927.9.9580	tubulin tyrosine ligase protein, putative
Tb927.9.9640	hypothetical protein, conserved
Tb927.9.9870	Kinesin-13 6, putative

Supplemental Table S1B. Proteins in the DRC1p proximity proteome that were identified in prior flagellum proteome studies.

Accession	Description	Accession	Description
Tb927.1.2100	Calpain-like protein 1.1	Tb927.3.1420	hypothetical protein, conserved
Tb927.1.2120	Calpain-like protein CALP1.3	Tb927.3.1670	hypothetical protein, conserved
Tb927.1.2670	paralyzed flagella 16	Tb927.3.1740	hypothetical protein, conserved
Tb927.1.2770	hypothetical protein, conserved	Tb927.3.1770	leucine-rich repeat protein (LRRP), putative
Tb927.1.4180	leucine-rich repeat protein (LRRP), putative	Tb927.3.1850	CMGC/MAPK protein kinase, putative
Tb927.1.4340	hypothetical protein, conserved	Tb927.3.1900	conserved protein, unknown function
Tb927.1.4470	hypothetical protein, conserved	Tb927.3.2070	hypothetical protein, conserved
Tb927.1.750	Metal-binding domain-containing protein	Tb927.3.2600	ATP-dependent DEAD/H RNA helicase, putative
Tb927.1.990	Enkuring domain-containig protein	Tb927.3.3000	Putative intraflagellar transport protein C3
Tb927.10.10630	hypothetical protein, conserved	Tb927.3.3200	Domain of unknown function (DUF4586), putative
Tb927.10.11080	paraflagellar rod protein	Tb927.3.3560	U-box domain containing protein, putative
Tb927.10.11250	Coiled-coil domain-containing protein 40 homolog	Tb927.3.3690	flagellar radial spoke protein-like, putative
Tb927.10.1130	dynein arm light chain, axonemal, putative	Tb927.3.4270	hypothetical protein, conserved
Tb927.10.11310	intraflagellar transport protein 57/55	Tb927.3.4290	Paraflagellar rod protein 1-1
Tb927.10.11340	hypothetical protein, conserved	Tb927.3.4300	Paraflagellar rod protein 1-2
Tb927.10.11580	predicted WD40 repeat protein	Tb927.3.4310	Paraflagellar rod protein 1-3
Tb927.10.1170	intraflagellar transport protein 172	Tb927.3.4330	Paraflagellar rod protein 1-5
Tb927.10.11780	hypothetical protein, conserved	Tb927.3.4320	Paraflagellar rod protein 1-4
Tb927.10.11850	WD domain, G-beta repeat, putative	Tb927.3.4500	Fumarate hydratase class I, cytosolic
Tb927.10.12130	Ubiquitin family, putative	Tb927.3.4750	Aminopeptidase M1, putative
Tb927.10.13550	hypothetical protein, conserved	Tb927.3.4960	kinesin, putative
Tb927.10.13780	Glycogen synthase kinase 3 short	Tb927.3.4970	hypothetical protein, conserved
Tb927.10.13960	paralyzed flagella protein 20	Tb927.3.4980	hypothetical protein, conserved, frameshift
Tb927.10.1400	hypoxanthine-guanine phosphoribosyltransferase	Tb927.3.5020	Flagellar Member 6
Tb927.10.14000	aconitase	Tb927.3.5310	paraflagellar rod protein
Tb927.10.14340	hypothetical protein, conserved	Tb927.3.5490	Putative intraflagellar transport protein B2
Tb927.10.14460	leucine-rich repeat protein (LRRP), putative	Tb927.3.5510	hypothetical protein, conserved
Tb927.10.14470	intraflagellar transport protein 140	Tb927.3.880	Ankyrin repeats (3 copies), putative
Tb927.10.14490	hypothetical protein, conserved	Tb927.3.930	dynein heavy chain, putative
Tb927.10.1450	plectin, putative	Tb927.4.1680	ZFP family member, putative
Tb927.10.14560	TPR repeat, putative	Tb927.4.1740	Component of motile flagella 41
Tb927.10.14570	kinesin motor domain containing protein, putative	Tb927.4.1890	hypothetical protein, conserved
Tb927.10.14610	leucine-rich repeat protein (LRRP), putative	Tb927.4.2140	Component of motile flagella 8
Tb927.10.14980	intraflagellar transport protein 52	Tb927.4.2300	hypothetical protein, conserved
Tb927.10.14990	intraflagellar transport protein 80	Tb927.4.2400	hypothetical protein, conserved
Tb927.10.15000	hypothetical protein, conserved	Tb927.4.2600	SUMO-interacting motif-containing protein
Tb927.10.15280	Domain of Unknown Function (DUF1042), putative	Tb927.4.2740	p25-alpha, putative
Tb927.10.15390	Flagellum attachment zone protein 7	Tb927.4.2920	hypothetical protein, conserved
Tb927.10.15770	hypothetical protein, conserved	Tb927.4.3620	serine threonine-protein phosphatase PP1, putative
Tb927.10.1890	cysteine peptidase, Clan CA, family C2, putative	Tb927.4.4370	hypothetical protein, conserved
Tb927.10.1970	CHAT domain containing protein, putative	Tb927.4.5000	TerD domain containing protein, putative
Tb927.10.2190	Protein of unknown function (DUF667), putative	Tb927.4.5340	Flagellum attachment zone protein 11
Tb927.10.2380	Tetratricopeptide repeat, putative	Tb927.4.5380	Cilia- and flagella-associated protein 43
Tb927.10.2640	intraflagellar transport protein 81	Tb927.4.560	Cytoplasmic dynein 2 heavy chain (DYNC2H1), putative
Tb927.10.2880	Voltage-dependent calcium channel subunit, putative	Tb927.4.620	hypothetical protein, conserved

Tb927.10.2950	hypothetical protein, conserved	Tb927.4.660	hypothetical protein, conserved
Tb927.10.3020	hypothetical protein, conserved	Tb927.4.670	conserved protein, unknown function
Tb927.10.3130	myotubularin-associated protein, putative	Tb927.4.870	dynein heavy chain, putative
Tb927.10.3310	Component of motile flagella 76b	Tb927.5.1100	peroxisome targeting signal 1 receptor
Tb927.10.360	hypothetical protein, conserved	Tb927.5.1230	hypothetical protein, conserved
Tb927.10.3820	hypothetical protein, conserved	Tb927.5.1680	hypothetical protein, conserved
Tb927.10.4170	hypothetical protein, conserved	Tb927.5.1690	hypothetical protein, conserved
Tb927.10.5230	ADP-ribosylation factor-like protein 13, putative	Tb927.5.1900	Outer dynein arm docking complex protein 1, putative
Tb927.10.5260	C2 domain containing protein, putative	Tb927.5.2270	Component of motile flagella 44
Tb927.10.5350	dynein heavy chain, putative	Tb927.5.2820	Serine/threonine-protein kinase NEK6, putative
Tb927.10.5430	Flagellar C1a complex subunit C1a-32, putative	Tb927.5.2850	radial spoke protein RSP2, putative
Tb927.10.5790	hypothetical protein, conserved	Tb927.5.2950	Component of motile flagella 3
Tb927.10.5940	NEK family Serine/threonine-protein kinase, putative	Tb927.5.295b	retrotransposon hotspot (RHS) protein 1
Tb927.10.5980	leucine-rich repeat protein (LRRP), putative	Tb927.5.3030	Intraflagellar transport protein 121
Tb927.10.6350	trypanin	Tb927.5.4390	hypothetical protein, conserved
Tb927.10.6960	hypothetical protein, conserved	Tb927.5.440	trans-sialidase, putative
Tb927.10.7120	hypothetical protein, conserved	Tb927.5.4470	hypothetical protein, conserved
Tb927.10.7230	Flagellar Member 1	Tb927.5.4480	paraflagellar rod component par4, putative
Tb927.10.7290	Flagellar Member 2	Tb927.5.500	hypothetical protein, conserved
Tb927.10.7350	hypothetical protein, conserved	Tb927.5.800	casein kinase I, isoform 2
Tb927.10.7510	chromatin binding protein, putative	Tb927.6.1660	hypothetical protein, conserved
Tb927.10.7690	Component of motile flagella 4	Tb927.6.1720	hypothetical protein, conserved
Tb927.10.7880	Sperm tail/Sperm tail C-terminal domain containing protein, putative	Tb927.6.2220	hypothetical protein, conserved
Tb927.10.8000	hypothetical protein, conserved	Tb927.6.3150	Hydin
Tb927.10.830	adenylate kinase, putative	Tb927.6.3500	endosomal trafficking protein RME-8, putative
Tb927.10.8780	AAA domain containing protein, putative	Tb927.6.3670	paraflagellar rod component, putative
Tb927.10.890	kinesin, putative	Tb927.6.3820	hypothetical protein, conserved
Tb927.10.8930	paraflagellar rod component, putative	Tb927.6.3920	hypothetical protein, conserved
Tb927.10.9080	pteridine transporter, putative	Tb927.6.410	hypothetical protein, conserved
Tb927.10.9570	paraflagellar rod component, putative	Tb927.6.4440	RNA-binding protein 42 (RNA-binding motif protein 42)
Tb927.10.9640	hypothetical protein, conserved	Tb927.6.4520	Tumour suppressor, Mitostatin, putative
Tb927.10.9650	Nucleoporin NUP152	Tb927.6.4610	Ankyrin repeats (3 copies), putative
Tb927.11.10200	hypothetical protein, conserved	Tb927.6.4710	calmodulin, putative
Tb927.11.10380	hypothetical protein, conserved	Tb927.6.5030	Component of motile flagella protein 46
Tb927.11.10430	hypothetical protein, conserved	Tb927.6.620	hypothetical protein, conserved
Tb927.11.10540	hypothetical protein, conserved	Tb927.7.1310	hypothetical protein, conserved
Tb927.11.10780	Voltage-dependent anion channel, putative	Tb927.7.1430	leucine-rich repeat protein (LRRP), putative
Tb927.11.1090	calpain-like protein, putative	Tb927.7.1830	hypothetical protein, conserved
Tb927.11.10900	Component of motile flagella 9	Tb927.7.2120	hypothetical protein, conserved
Tb927.11.11010	hypothetical protein, conserved	Tb927.7.2320	cyclic nucleotide-binding domain containing protein, putative
Tb927.11.11220	dynein heavy chain, putative	Tb927.7.2790	Component of motile flagella 10
Tb927.11.11280	hypothetical protein, conserved	Tb927.7.3370	Intraflagellar transport protein 74, putative
Tb927.11.1150	radial spoke protein 3	Tb927.7.3560	Component of motile flagella 7
Tb927.11.12540	leucine-rich repeat protein (LRRP), putative	Tb927.7.3740	SUMO-interacting motif-containing protein
Tb927.11.12560	hypothetical protein	Tb927.7.4020	protein phosphatase 2C, putative
Tb927.11.12840	Domain of unknown function (DUF4486), putative	Tb927.7.4100	Domain of unknown function (DUF4201), putative
Tb927.11.12860	cAMP response protein, putative	Tb927.7.4110	kinesin, putative
Tb927.11.12860	cAMP response protein, putative	Tb927.7.4390	threonine synthase, putative
Tb927.11.13020	calmodulin	Tb927.7.4510	Domain of unknown function (DUF4201), putative

Tb927.11.13030	calmodulin	Tb927.7.4740	hypothetical protein, conserved
Tb927.11.13040	calmodulin	Tb927.7.4840	hypothetical protein, conserved
Tb927.11.13050	calmodulin	Tb927.7.5340	cAMP response protein 3
Tb927.11.1340	Flagella connector protein 1	Tb927.7.5660	Outer dynein arm docking complex protein 2, putative
Tb927.11.13460	hypothetical protein, conserved	Tb927.7.5940	Protein Associated with Differentiation
Tb927.11.13670	hypothetical protein, conserved	Tb927.7.6280	Domain of unknown function (DUF3508), putative
Tb927.11.13900	Centrin-5	Tb927.7.6290	kinesin, putative
Tb927.11.1420	hypothetical protein, conserved	Tb927.7.6340	hypothetical protein, conserved
Tb927.11.14210	conserved protein	Tb927.7.6640	hypothetical protein, conserved
Tb927.11.14240	WD domain, G-beta repeat, putative	Tb927.7.6910	Phosphatidylinositol 4-phosphate 5-kinase, putative
Tb927.11.1430	Component of motile flagella 2	Tb927.7.6950	hypothetical protein, conserved
Tb927.11.14440	Pentatricopeptide repeat protein (PPR), putative	Tb927.7.6970	paraflagellar rod protein, putative
Tb927.11.14450	hypothetical protein, conserved	Tb927.7.7200	hypothetical protein, conserved
Tb927.11.14880	Paraflagellar Rod Proteome Component 9, putative	Tb927.7.7240	leucine-rich repeat protein (LRRP), putative
Tb927.11.14970	hypothetical protein, conserved	Tb927.7.7250	Ankyrin repeats (3 copies), putative
Tb927.11.15100	Tb5.20	Tb927.7.750	hypothetical protein, conserved
Tb927.11.15120	hypothetical protein, conserved	Tb927.7.920	Inner arm dynein 5-1
Tb927.11.15190	Domain of unknown function (DUF4200), putative	Tb927.8.1050	SUMO-interacting motif-containing protein
Tb927.11.15220	hypothetical protein, conserved	Tb927.8.1540	hypothetical protein, conserved
Tb927.11.15370	Importin subunit beta-4, putative	Tb927.8.1550	paraflagellar rod component, putative
Tb927.11.15730	cyclic nucleotide-binding domain containing protein, putative	Tb927.8.1560	hypothetical protein, conserved
Tb927.11.16090	Outer dynein arm docking complex protein 2, putative	Tb927.8.1950	hypothetical protein, conserved
Tb927.11.16830	Rab-like 5, small G protein	Tb927.8.2130	cyclic nucleotide-binding domain containing protein, putative
Tb927.11.16880	leucine-rich repeat protein (LRRP), putative	Tb927.8.2160	p-glycoprotein
Tb927.11.16890	Tetratricopeptide-like helical domain containing protein	Tb927.8.2390	hypothetical protein, conserved
Tb927.11.16900	hypothetical protein	Tb927.8.2620	SUMO-interacting motif-containing protein
Tb927.11.1740	intraflagellar transport protein 88	Tb927.8.2640	ubiquitin-activating enzyme E1, putative
Tb927.11.2140	cyclic nucleotide-binding domain containing protein, putative	Tb927.8.3250	dynein heavy chain, putative
Tb927.11.2270	inner dynein arm I1 intermediate chain, axonemal	Tb927.8.4050	FLA1-binding protein
Tb927.11.2430	Cytoplasmic dynein 2 heavy chain (DYNC2H2), putative	Tb927.8.4400	Outer dynein arm docking complex protein 1, putative
Tb927.11.2540	hypothetical protein, conserved	Tb927.8.4460	Domain of Unknown Function (DUF1042), putative
Tb927.11.260	Putative intraflagellar transport protein F6	Tb927.8.4580	Basal body protein
Tb927.11.2770	hypothetical protein, conserved	Tb927.8.4780	Flagellar Member 3
Tb927.11.2790	Solute carrier (proton/amino acid symporter), TRAMD3 or PAT1, putative	Tb927.8.4870	Component of motile flagella 6
Tb927.11.2830	hypothetical protein, conserved	Tb927.8.4970	Paraflagellar rod protein 2-1
Tb927.11.3250	dynein heavy chain, putative	Tb927.8.4980	Paraflagellar rod protein 2-2
Tb927.11.3360	Component of motile flagella 22	Tb927.8.4990	Paraflagellar rod protein 2-3
Tb927.11.3500	Dpy-30 motif containing protein, putative	Tb927.8.5000	Paraflagellar rod protein 2-4
Tb927.11.3710	hypothetical protein, conserved	Tb927.8.5010	Paraflagellar rod protein 2-5
Tb927.11.3920	hypothetical protein, conserved	Tb927.8.5300	hypothetical protein, conserved
Tb927.11.4000	hypothetical protein, conserved	Tb927.8.5460	Flagellar calcium-binding 44 kDa protein
Tb927.11.4420	hypothetical protein, conserved	Tb927.8.610	hypothetical protein, conserved
Tb927.11.4480	Flagellar radial spoke protein 4/6	Tb927.8.6260	hypothetical protein, conserved
Tb927.11.450	hypothetical protein, conserved	Tb927.8.6270	hypothetical protein, conserved

Tb927.11.4550	Domain of unknown function (DUF4200), putative	Tb927.8.640	Dpy-30 motif containing protein, putative
Tb927.11.4610	protein kinase A regulatory subunit	Tb927.8.6660	paraflagellar rod component, putative
Tb927.11.550	Suppressor of clathrin deficiency 6, putative	Tb927.8.7540	hypothetical protein
Tb927.11.5750	SUMO-interacting motif-containing protein	Tb927.8.7800	hypothetical protein, conserved
Tb927.11.5890	Domain of unknown function (DUF4505), putative	Tb927.8.7950	Flagellar Member 4
Tb927.11.6050	Flagellar-associated PapD-like, putative	Tb927.8.8200	Flagellar-associated protein 59, putative
Tb927.11.6070	hypothetical protein, conserved	Tb927.8.8300	amino acid transporter, putative
Tb927.11.610	hypothetical protein, conserved	Tb927.8.940	Flagella connector protein 1
Tb927.11.6160	eukaryotic release factor 3, putative	Tb927.9.10370	TAX-1
Tb927.11.6370	leucine-rich repeat protein (LRRP), putative	Tb927.9.10890	Regulatory subunit of type II PKA R-subunit, putative
Tb927.11.6440	hypothetical protein, conserved	Tb927.9.11230	calmodulin-like protein, putative
Tb927.11.6460	hypothetical protein, conserved	Tb927.9.11580	glycosomal membrane protein
Tb927.11.6710	predicted tetratricopeptide repeat (TPR) protein	Tb927.9.11600	glycosomal membrane protein
Tb927.11.6870	14-3-3 protein 2	Tb927.9.12830	hypothetical protein, conserved
Tb927.11.6920	hypothetical protein, conserved	Tb927.9.12990	hypothetical protein, conserved
Tb927.11.7240	nexin-dynein regulatory complex 2	Tb927.9.13070	Heat shock factor binding 1 domain-containing protein
Tb927.11.7740	dynein light chain, putative	Tb927.9.13440	Flagellar Member 5
Tb927.11.7940	calmodulin, putative	Tb927.9.1350	hypothetical protein, conserved
Tb927.11.8030	hypothetical protein, conserved	Tb927.9.13820	kinetoplastid membrane protein 11-3
Tb927.11.8160	dynein heavy chain, putative	Tb927.9.13860	kinetoplastid membrane protein 11-2
Tb927.11.9400	hypothetical protein, conserved	Tb927.9.13920	kinetoplastid membrane protein 11-1
Tb927.11.9440	Component of motile flagella 63	Tb927.9.15050	nexin-dynein regulatory complex 4
Tb927.2.1850	Tetratricopeptide repeat, putative	Tb927.9.1600	hypothetical protein, conserved
Tb927.2.240	retrotransposon hot spot protein 5 (RHS5), putative	Tb927.9.1750	Fibronectin type III domain containing protein, putative
Tb927.2.3020	Component of motile flagella 76	Tb927.9.1880	WD domain, G-beta repeat, putative
Tb927.2.4050	hypothetical protein, conserved	Tb927.9.1980	hypothetical protein, conserved
Tb927.2.4060	Dynein intermediate chain 138	Tb927.9.2940	hypothetical protein, conserved
Tb927.2.4330	paraflagellar rod protein 5, putative	Tb927.9.5040	cAMP-specific phosphodiesterase
Tb927.2.4520	hypothetical protein, conserved	Tb927.9.5100	cAMP-specific phosphodiesterase
Tb927.2.4810	Component of motile flagella 5	Tb927.9.5410	hypothetical protein, conserved
Tb927.2.5270	dynein heavy chain, putative	Tb927.9.6130	calmodulin, putative
Tb927.2.5760	Flagellar Member 8	Tb927.9.6290	arginine kinase
Tb927.3.1040	hypothetical protein, conserved	Tb927.9.7180	adenosine monophosphate deaminase, putative
Tb927.3.1060	cAMP Response Protein 4	Tb927.9.7470	purine nucleoside transporter
Tb927.3.1110	Putative intraflagellar transport protein A1	Tb927.9.7550	adenylosuccinate lyase, putative
Tb927.3.1200	hypothetical protein, conserved	Tb927.9.7720	predicted tetratricopeptide repeat protein
		Tb927.9.8740	Double RNA binding domain protein 3
		Tb927.9.8760	hypothetical protein, conserved
		Tb927.9.9740	AMP deaminase, putative
