# Supplementary material for: APEX2 Proximity Proteomics Resolves Flagellum Subdomains and Identifies Flagellum Tip-Specific Proteins in Trypanosoma brucei
Source: mSphere. 2021 Feb 10;6(1):e01090-20. doi: 10.1128/mSphere.01090-20 (PMC8141408; doi:10.1128/mSphere.01090-20)
Supplement: TABLE S2 [file mSphere.01090-20-st002.pdf]

Supplemental Table S2. Human homologues with disease association.

T. brucei gene	Human homologue	e-value*	Human gene product description	Human disease phenotype
Tb927.10.5380	XP_005247666.1	0	IFT122 – intraflagellar transport protein 122, homolog isoform X13	Craniοectodermal dysplasia 1 (OMIM #218330)
Tb927.4.3810	BAF82512.1	0	POLR2B – RNA polymerase II subunit B	Age-related macular degeneration [1], adult height [2, 3]
Tb927.7.7260	XP_011512661.1	4.64E-135	Kif6 – kinesin-like protein, isoform X4	ADHD [4], antibody response to smallpox vaccine [5], dental caries [6], childhood obesity [7]
Tb927.11.3140	AAH31244.1	5.35E-119	Dyrk4 – Dual-specificity tyrosine-(Y)-phosphorylation regulated kinase 4	Allergic rhinitis [8]
Tb927.8.5020	CAD38878.1	1.16E-116	VWA8 – von Willebrand factor A domain containing 8	Emphysema [9], tuberculosis risk [10]
Tb927.11.14680	NP_001341508.1	1.06E-113	ATR – serine/threonine-protein kinase, isoform 2	Familial cutaneous telangiectasia and cancer syndrome (OMIM #614564), Seckel syndrome 1 (OMIM #210600)
Tb927.11.6350	XP_006712094.1	4.90E-102	ATAD2B – ATPase family AAA domain-containing protein 2B, isoform X10	Pathophysiology of childhood obesity [7]
Tb927.3.4020	XP_016884319.1	2.00E-94	PI4KA – phosphatidylinositol 4-kinase alpha, isoform X4	Polymicrogyria, perisylvian, with cerebellar hypoplasia and arthrogryposis (OMIM #616531)
Tb927.10.2900	BAG37316.1	5.10E-78	KPNB1 – karyopherin subunit beta 1	Circulating levels of very long-chain saturated fatty acids [11], multiple sclerosis susceptibility [12]
Tb927.10.13000	EAW93986.1	8.13E-66	PDE1C – phosphodiesterase 1C, calmodulin-dependent 70kDa, isoform CRA_b	Autosomal dominant deafness (OMIM #618140), endometriosis [13], antiphospholipid antibodies [14]
Tb927.10.8390	AAH40929.1	3.35E-64	HERC1 – HECT and RLD domain containing E3 ubiquitin protein ligase family member 1	Macrocephaly, dysmorphic facies, and psychomotor retardation (OMIM #617011), neuronal pattern development [15]
Tb927.10.1510	AAH00779.2	2.47E-60	CNOT1 – CCR4-NOT transcription complex subunit 1	Holoprosencephaly 12 with/without pancreatic angenesis (OMIM #618500), associated with schizophrenia [16], QT interval duration [17-20], PR interval, and QRS duration [21]
Tb927.10.13000	AAD50326.1	5.70E-59	RAD50 – RAD50 double strand break repair protein	Nijmegen breakage syndrome-like disorder (OMIM #613078), IgE levels [22], asthma [23, 24] and psoriasis [25]
Tb927.9.9580	AAF23275.2	7.80E-56	TTLL5 – tubulin tyrosine ligase like 5	Cone-rod dystrophy 19 (OMIM #615860), susceptibility to ischemic stroke and coronary heart disease [26], adult height [27]

Tb927.8.5970	BAG37520	1.67E-50	CUL4B – cullin-4B isoform X3	Syndromic X-linked mental retardation, Cabezas type (OMIM #300354)
Tb927.8.1890	AAA35730.1	2.44E-49	CYC1 – cytochrome c1, partial	Mitochondrial complex III deficiency, nuclear type 6 (OMIM #615453)
Tb927.11.12430	AAT38107.1	4.10E-46	CDC14A – cell division cycle 14 homolog A	Deafness, autosomal recessive 32, with/without immotile sperm (OMIM #608653)
Tb927.8.4510	BAG37189.1	1.05E-36	CCDC65 – coiled-coil domain containing 65	Primary ciliary dyskinesia (OMIM #615504)
Tb927.5.950	CAA09375.1	1.87E-32	GLRX3 – glutaredoxin 3	Susceptibility to HIV-1 [28]
Tb927.10.9380	BAC04112.1	1.17E-23	UBXN11 – UBX domain protein 11	Pathophysiology of childhood obesity [7]
Tb927.9.9580	EAW97654.1	2.98E-20	UTP20 – UTP20 small subunit (SSU) processome component	Influences neurodegeneration in Alzheimer's disease [29], associated with subclinical atherosclerosis [30], onset of alcohol dependence [31]
Tb927.8.5290	BAG64996.1	2.61E-17	ITCH – itchy E3 ubiquitin protein ligase	Autoimmune disease, syndromic multisystem (OMIM #613385)
Tb927.5.3650	NP_055710.2	2.70E-17	CEP162 – centrosomal protein of 162 kDa isoform a	Associated with diabetic retinopathy [32]
Tb927.5.4110	AAH50721.1	7.08E-14	CEP104 – centrosomal protein 104	Joubert syndrome 25 (OMIM #616781)
Tb927.5.1920	EAW64669.1	6.41E-13	CCDC13 – coiled-coil domain containing 13, isoform CRA_a	Clozapine-induced agranulocytosis [33], chronic periodontitis [34], pathophysiology of childhood obesity [7]
Tb927.10.970	EAW91271.1	4.26E-12	ASPM – assembly facto for spindle microtubule	Primary autosomal recessive microcephaly (OMIM #608716), lupus nephritis susceptibility [35], ischemic stroke [36]
Tb927.7.290	XP_005250264.1	1.18E-09	FBXL13 – F-box/LRR-repeat protein 13 isoform X5	Blood pressure response to interventions [37]
Tb927.7.1560	AAI03916.1	1.23E-09	TTC6 – tetratricopeptide repeat domain 6	Allergy-specific susceptibility [38]
Tb927.8.4510	AAH36816.1	2.49E-06	NME8 – NME/NM23 family member 8	Primary ciliary dyskinesia (OMIM #610852), risk of fracture [39], susceptibility for Alzheimer's disease [40]
Tb927.8.6870	BAG06714.1	9.04E-05	MYO5B – myosin VB	Congenital microvillous atrophy (OMIM #251850)
Tb927.10.5380	EAW91274.1	0.000116493	ASPM – assembly factor for spindle microtubules	Primary autosomal recessive microcephaly (OMIM #608716), ischemic stroke [36], lupus nephritis susceptibility in women [35]
Tb927.6.4750	BAG56787.1	0.000868412	BUB3 – BUB3 mitotic checkpoint protein	Age of menarche and natural menopause [41]
Tb927.11.4210	AAC18038.1	0.00394461	STUB1 – STIP1 homology and U-box containing protein	Autosomal recessive 16 spinocerebellar ataxia (OMIM #615768), spinocerebellar ataxia 48 (OMIM #618093)
Tb927.3.1190	EAW64508.1	0.00498254	DLEC1 – DLEC1 cilia and flagella associated protein	Lung cancer, malignant tumor of esophagus*

Tb927.5.3650	EAW71112.1	0.00600032	MLPH melanophilin, isoform CRA_a	Griscelli syndrome type 3 (OMIM #609227), prostate cancer susceptibility loci [42]
Tb927.11.6430	XP_011524505.1	0.0250287	CCDC68 – coiled-coil domain-containing protein 68, isoform X3	Schizophrenia [43], severe diabetic retinopathy [44], psychiatric disorders [45]
Tb927.3.4510	BAH13910.1	0.0509298	TRAF3 – TNF receptor associated factor 3	Schizophrenia [46], susceptibility to herpes simplex encephalitis (OMIM #614849)
Tb927.8.5020	BAA25448.1	0.0592341	IQSEC2 – IQ motif and Sec7 domain ArfGEF 2	X-linked 1 mental retardation (OMIM #309530), haploinsufficeincy [47]

1. Arakawa S, Takahashi A, Ashikawa K, Hosono N, Aoi T, Yasuda M, Oshima Y, Yoshida S, Enaida H, Tsuchihashi T, Mori K, Honda S, Negi A, Arakawa A, Kadonosono K, Kiyohara Y, Kamatani N, Nakamura Y, Ishibashi T, Kubo M. 2011. *Genome-wide association study identifies two susceptibility loci for exudative age-related macular degeneration in the Japanese population.* Nat Genet. **43**(10): p. 1001-4.
2. Lango Allen H, Estrada K, Lettre G, Berndt SI, Weedon MN, Rivadeneira F, Willer CJ, Jackson AU, Vedantam S, Raychaudhuri S, Ferreira T, Wood AR, Weyant RJ, Segrè AV, Speliotes EK, Wheeler E, Soranzo N, Park JH, Yang J, Gudbjartsson D, Heard-Costa NL, Randall JC, Qi L, Vernon Smith A, Mägi R, Pastinen T, Liang L, Heid IM, Luan J, Thorleifsson G, Winkler TW, Goddard ME, Sin Lo K, Palmer C, Workalemahu T, Aulchenko YS, Johansson A, Zillikens MC, Feitosa MF, Esko T, Johnson T, Ketkar S, Kraft P, Mangino M, Prokopenko I, Absher D, Albrecht E, Ernst F, Glazer NL, Hayward C, Hottenga JJ, Jacobs KB, Knowles JW, Kutalik Z, Monda KL, Polasek O, Preuss M, Rayner NW, Robertson NR, Steinthorsdottir V, Tyrer JP, Voight BF, Wiklund F, Xu J, Zhao JH, Nyholt DR, Pellikka N, Perola M, Perry JR, Surakka I, Tammesoo ML, Altmaier EL, Amin N, Aspelund T, Bhangale T, Boucher G, Chasman DI, Chen C, Coin L, Cooper MN, Dixon AL, Gibson Q, Grundberg E, Hao K, Juhani Junttila M, Kaplan LM, Kettunen J, König IR, Kwan T, Lawrence RW, Levinson DF, Lorentzon M, McKnight B, Morris AP, Müller M, Suh Ngwa J, Purcell S, Rafelt S, Salem RM, Salvi E, Sanna S, Shi J, Sovio U, Thompson JR, Turchin MC, Vandenput L, Verlaan DJ, Vitart V, White CC, Ziegler A, Almgren P, Balmforth AJ, Campbell H, Citterio L, De Grandi A, Dominiczak A, Duan J, Elliott P, Elosua R, Eriksson JG, Freimer NB, Geus EJ, Glorioso N, Haiqing S, Hartikainen AL, Havulinna AS, Hicks AA, Hui J, Igl W, Illig T, Jula A, Kajantie E, Kilpeläinen TO, Koiranen M, Kolcic I, Koskinen S, Kovacs P, Laitinen J, Liu J, Lokki ML, Marusic A, Maschio A, Meitinger T, Mulas A, Paré G, Parker AN, Peden JF, Petersmann A, Pichler I, Pietiläinen KH, Pouta A, Ridderstråle M, Rotter JI, Sambrook JG, Sanders AR, Schmidt CO, Sinisalo J, Smit JH, Stringham HM, Bragi Walters G, Widen E, Wild SH, Willemsen G, Zagato L, Zgaga L, Zitting P, Alavere H, Farrall M, McArdle WL, Nelis M, Peters MJ, Ripatti S, van Meurs JB, Aben KK, Ardlie KG, Beckmann JS, Beilby JP, Bergman RN, Bergmann S, Collins FS, Cusi D, den Heijer M, Eiriksdottir G, Gejman PV, Hall AS, Hamsten A, Huikuri HV, Iribarren C, Kähönen M, Kaprio J, Kathiresan S, Kiemeney L, Kocher T, Launer LJ, Lehtimäki T, Melander O, Mosley TH, Jr., Musk AW, Nieminen MS, O'Donnell CJ, Ohlsson C, Oostra B, Palmer LJ, Raitakari O, Ridker PM, Rioux JD, Rissanen A, Rivolta C, Schunkert H, Shuldiner AR, Siscovick DS, Stumvoll M, Tönjes A, Tuomilehto J, van Ommen GJ, Viikari J, Heath AC, Martin NG, Montgomery GW, Province MA, Kayser M, Arnold AM, Atwood LD, Boerwinkle E, Chanock SJ, Deloukas P, Gieger C, Grönberg H, Hall P, Hattersley AT, Hengstenberg C, Hoffman W, Lathrop GM, Salomaa V, Schreiber S, Uda M, Waterworth D, Wright AF, Assimes TL, Barroso I, Hofman A, Mohlke KL, Boomsma DI, Caulfield MJ, Cupples LA, Erdmann J, Fox CS, Gudnason V, Gyllensten U, Harris TB, Hayes RB, Jarvelin MR, Mooser V, Munroe PB, Ouwehand WH, Penninx BW, Pramstaller PP, Quertermous T, Rudan I, Samani NJ, Spector TD, Völzke H, Watkins H, Wilson JF, Groop LC, Haritunians T, Hu FB, Kaplan RC, Metspalu A, North KE, Schlessinger D, Wareham NJ, Hunter DJ, O'Connell JR, Strachan DP, Wichmann HE, Borecki IB, van Duijn CM, Schadt EE, Thorsteinsdottir U, Peltonen L, Uitterlinden AG, Visscher PM, Chatterjee N, Loos RJ, Boehnke M, McCarthy MI, Ingelsson E, Lindgren CM, Abecasis GR, Stefansson K, Frayling TMHirschhorn JN. 2010. *Hundreds of variants clustered in genomic loci and biological pathways affect human height.* Nature. **467**(7317): p. 832-8.
3. He M, Xu M, Zhang B, Liang J, Chen P, Lee JY, Johnson TA, Li H, Yang X, Dai J, Liang L, Gui L, Qi Q, Huang J, Li Y, Adair LS, Aung T, Cai Q, Cheng CY, Cho MC, Cho YS, Chu M, Cui B, Gao YT, Go MJ, Gu D, Gu W, Guo H, Hao Y, Hong J, Hu Z, Hu Y, Huang J, Hwang JY, Ikram MK, Jin G, Kang DH, Khor CC, Kim BJ, Kim HT, Kubo M, Lee J, Lee J, Lee NR, Li R, Li J, Liu J, Longe J, Lu W, Lu X, Miao X, Okada Y, Ong RT, Qiu G, Seielstad M, Sim X, Song H, Takeuchi F, Tanaka T,

Taylor PR, Wang L, Wang W, Wang Y, Wu C, Wu Y, Xiang YB, Yamamoto K, Yang H, Liao M, Yokota M, Young T, Zhang X, Kato N, Wang QK, Zheng W, Hu FB, Lin D, Shen H, Teo YY, Mo Z, Wong TY, Lin X, Mohlke KL, Ning G, Tsunoda T, Han BG, Shu XO, Tai ES, Wu T, Qi L. 2015. *Meta-analysis of genome-wide association studies of adult height in East Asians identifies 17 novel loci.* Hum Mol Genet. **24**(6): p. 1791-800.

4. Sonuga-Barke EJ, Lasky-Su J, Neale BM, Oades R, Chen W, Franke B, Buitelaar J, Banaschewski T, Ebstein R, Gill M, Anney R, Miranda A, Mulas F, Roeyers H, Rothenberger A, Sergeant J, Steinhausen HC, Thompson M, Asherson P, Faraone SV. 2008. *Does parental expressed emotion moderate genetic effects in ADHD? An exploration using a genome wide association scan.* Am J Med Genet B Neuropsychiatr Genet. **147b**(8): p. 1359-68.
5. Ovsyannikova IG, Kennedy RB, O'Byrne M, Jacobson RM, Pankratz VS, Poland GA. 2012. *Genome-wide association study of antibody response to smallpox vaccine.* Vaccine. **30**(28): p. 4182-9.
6. Zeng Z, Feingold E, Wang X, Weeks DE, Lee M, Cuenco DT, Broffitt B, Weyant RJ, Crout R, McNeil DW, Levy SM, Marazita ML, Shaffer JR. 2014. *Genome-wide association study of primary dentition pit-and-fissure and smooth surface caries.* Caries Res. **48**(4): p. 330-8.
7. Comuzzie AG, Cole SA, Laston SL, Voruganti VS, Haack K, Gibbs RA, Butte NF. 2012. *Novel genetic loci identified for the pathophysiology of childhood obesity in the Hispanic population.* PLoS One. **7**(12): p. e51954.
8. Bunyavanich S, Schadt EE, Himes BE, Lasky-Su J, Qiu W, Lazarus R, Ziniti JP, Cohain A, Linderman M, Torgerson DG, Eng CS, Pino-Yanes M, Padhukasahasram B, Yang JJ, Mathias RA, Beaty TH, Li X, Graves P, Romieu I, Navarro Bdel R, Salam MT, Vora H, Nicolae DL, Ober C, Martinez FD, Bleecker ER, Meyers DA, Gauderman WJ, Gilliland F, Burchard EG, Barnes KC, Williams LK, London SJ, Zhang B, Raby BA, Weiss ST. 2014. *Integrated genome-wide association, coexpression network, and expression single nucleotide polymorphism analysis identifies novel pathway in allergic rhinitis.* BMC Med Genomics. **7**: p. 48.
9. Castaldi PJ, Cho MH, San José Estépar R, McDonald ML, Laird N, Beaty TH, Washko G, Crapo JD, Silverman EK. 2014. *Genome-wide association identifies regulatory Loci associated with distinct local histogram emphysema patterns.* Am J Respir Crit Care Med. **190**(4): p. 399-409.
10. Chimusa ER, Zaitlen N, Daya M, Möller M, van Helden PD, Mulder NJ, Price AL, Hoal EG. 2014. *Genome-wide association study of ancestry-specific TB risk in the South African Coloured population.* Hum Mol Genet. **23**(3): p. 796-809.
11. Lemaitre RN, King IB, Kabagambe EK, Wu JH, McKnight B, Manichaikul A, Guan W, Sun Q, Chasman DI, Foy M, Wang L, Zhu J, Siscovick DS, Tsai MY, Arnett DK, Psaty BM, Djousse L, Chen YD, Tang W, Weng LC, Wu H, Jensen MK, Chu AY, Jacobs DR, Jr., Rich SS, Mozaffarian D, Steffen L, Rimm EB, Hu FB, Ridker PM, Fornage M, Friedlander Y. 2015. *Genetic loci associated with circulating levels of very long-chain saturated fatty acids.* J Lipid Res. **56**(1): p. 176-84.
12. Patsopoulos NA, Esposito F, Reischl J, Lehr S, Bauer D, Heubach J, Sandbrink R, Pohl C, Edan G, Kappos L, Miller D, Montalbán J, Polman CH, Freedman MS, Hartung HP, Arnason BG, Comi G, Cook S, Filippi M, Goodin DS, Jeffery D, O'Connor P, Ebers GC, Langdon D, Reder AT, Traboulsee A, Zipp F, Schimrigk S, Hillert J, Bahlo M, Booth DR, Broadley S, Brown MA, Browning BL, Browning SR, Butzkueven H, Carroll WM, Chapman C, Foote SJ, Griffiths L, Kermode AG, Kilpatrick TJ, Lechner-Scott J, Marriott M, Mason D, Moscato P, Heard RN, Pender MP, Perreau VM, Perera D, Rubio JP, Scott RJ, Slee M, Stankovich J, Stewart GJ, Taylor BV, Tubridy N, Willoughby E, Wiley J, Matthews P, Boneschi FM, Compston A, Haines J, Hauser SL, McCauley J, Ivinson A, Oksenberg JR, Pericak-Vance M, Sawcer SJ, De Jager PL, Hafler DA, de Bakker PI. 2011. *Genome-wide meta-analysis identifies novel multiple sclerosis susceptibility loci.* Ann Neurol. **70**(6): p. 897-912.
13. Albertsen HM, Chettier R, Farrington P, Ward K. 2013. *Genome-wide association study link novel loci to endometriosis.* PLoS One. **8**(3): p. e58257.
14. Kamboh MI, Wang X, Kao AH, Barmada MM, Clarke A, Ramsey-Goldman R, Manzi S, Demirci FY. 2013. *Genome-wide association study of antiphospholipid antibodies.* Autoimmune Dis. **2013**: p. 761046.
15. Larsson M, Duffy DL, Zhu G, Liu JZ, Macgregor S, McRae AF, Wright MJ, Sturm RA, Mackey DA, Montgomery GW, Martin NG, Medland SE. 2011. *GWAS findings for human iris patterns: associations with variants in genes that influence normal neuronal pattern development.* Am J Hum Genet. **89**(2): p. 334-43.
16. 2014. *Biological insights from 108 schizophrenia-associated genetic loci.* Nature. **511**(7510): p. 421-7.

17. Kim JW, Hong KW, Go MJ, Kim SS, Tabara Y, Kita Y, Tanigawa T, Cho YS, Han BG, Oh B. 2012. *A common variant in SLC8A1 is associated with the duration of the electrocardiographic QT interval.* Am J Hum Genet. **91**(1): p. 180-4.
18. Newton-Cheh C, Eijgelsheim M, Rice KM, de Bakker PI, Yin X, Estrada K, Bis JC, Marciante K, Rivadeneira F, Noseworthy PA, Sotoodehnia N, Smith NL, Rotter JI, Kors JA, Witteman JC, Hofman A, Heckbert SR, O'Donnell CJ, Uitterlinden AG, Psaty BM, Lumley T, Larson MG, Stricker BH. 2009. *Common variants at ten loci influence QT interval duration in the QTGEN Study.* Nat Genet. **41**(4): p. 399-406.
19. Pfeufer A, Sanna S, Arking DE, Müller M, Gateva V, Fuchsberger C, Ehret GB, Orrú M, Pattaro C, Köttgen A, Perz S, Usala G, Barbalic M, Li M, Pütz B, Scuteri A, Prineas RJ, Sinner MF, Gieger C, Najjar SS, Kao WH, Mühleisen TW, Dei M, Happle C, Möhlenkamp S, Crisponi L, Erbel R, Jöckel KH, Naitza S, Steinbeck G, Marroni F, Hicks AA, Lakatta E, Müller-Myhsok B, Pramstaller PP, Wichmann HE, Schlessinger D, Boerwinkle E, Meitinger T, Uda M, Coresh J, Kääb S, Abecasis GR, Chakravarti A. 2009. *Common variants at ten loci modulate the QT interval duration in the QTSCD Study.* Nat Genet. **41**(4): p. 407-14.
20. Arking DE, Pulit SL, Crotti L, van der Harst P, Munroe PB, Koopmann TT, Sotoodehnia N, Rossin EJ, Morley M, Wang X, Johnson AD, Lundby A, Gudbjartsson DF, Noseworthy PA, Eijgelsheim M, Bradford Y, Tarasov KV, Dörr M, Müller-Nurasyid M, Lahtinen AM, Nolte IM, Smith AV, Bis JC, Isaacs A, Newhouse SJ, Evans DS, Post WS, Waggott D, Lyytikäinen LP, Hicks AA, Eisele L, Ellinghaus D, Hayward C, Navarro P, Ulivi S, Tanaka T, Tester DJ, Chatel S, Gustafsson S, Kumari M, Morris RW, Naluai Å T, Padmanabhan S, Kluttig A, Strohmer B, Panayiotou AG, Torres M, Knoflach M, Hubacek JA, Slowikowski K, Raychaudhuri S, Kumar RD, Harris TB, Launer LJ, Shuldiner AR, Alonso A, Bader JS, Ehret G, Huang H, Kao WH, Strait JB, Macfarlane PW, Brown M, Caulfield MJ, Samani NJ, Kronenberg F, Willeit J, Smith JG, Greiser KH, Meyer Zu Schwabedissen H, Werdan K, Carella M, Zelante L, Heckbert SR, Psaty BM, Rotter JI, Kolcic I, Polašek O, Wright AF, Griffin M, Daly MJ, Arnar DO, Hólm H, Thorsteinsdottir U, Denny JC, Roden DM, Zuvich RL, Emilsson V, Plump AS, Larson MG, O'Donnell CJ, Yin X, Bobbo M, D'Adamo AP, Iorio A, Sinagra G, Carracedo A, Cummings SR, Nalls MA, Jula A, Kontula KK, Marjamaa A, Oikarinen L, Perola M, Porthan K, Erbel R, Hoffmann P, Jöckel KH, Kälsch H, Nöthen MM, den Hoed M, Loos RJ, Thelle DS, Gieger C, Meitinger T, Perz S, Peters A, Prucha H, Sinner MF, Waldenberger M, de Boer RA, Franke L, van der Vleuten PA, Beckmann BM, Martens E, Bardai A, Hofman N, Wilde AA, Behr ER, Dalageorgou C, Giudicessi JR, Medeiros-Domingo A, Barc J, Kyndt F, Probst V, Ghidoni A, Insolia R, Hamilton RM, Scherer SW, Brandimarto J, Margulies K, Moravec CE, del Greco MF, Fuchsberger C, O'Connell JR, Lee WK, Watt GC, Campbell H, Wild SH, El Mokhtari NE, Frey N, Asselbergs FW, Mateo Leach I, Navis G, van den Berg MP, van Veldhuisen DJ, Kellis M, Krijthe BP, Franco OH, Hofman A, Kors JA, Uitterlinden AG, Witteman JC, Kedenko L, Lamina C, Oostra BA, Abecasis GR, Lakatta EG, Mulas A, Orrú M, Schlessinger D, Uda M, Markus MR, Völker U, Snieder H, Spector TD, Ärnlöv J, Lind L, Sundström J, Syvänen AC, Kivimaki M, Kähönen M, Mononen N, Raitakari OT, Viikari JS, Adamkova V, Kiechl S, Brion M, Nicolaides AN, Paulweber B, Haerting J, Dominiczak AF, Nyberg F, Whincup PH, Hingorani AD, Schott JJ, Bezzina CR, Ingelsson E, Ferrucci L, Gasparini P, Wilson JF, Rudan I, Franke A, Mühleisen TW, Pramstaller PP, Lehtimäki TJ, Paterson AD, Parsa A, Liu Y, van Duijn CM, Siscovick DS, Gudnason V, Jamshidi Y, Salomaa V, Felix SB, Sanna S, Ritchie MD, Stricker BH, Stefansson K, Boyer LA, Cappola TP, Olsen JV, Lage K, Schwartz PJ, Kääb S, Chakravarti A, Ackerman MJ, Pfeufer A, de Bakker PINewton-Cheh C. 2014. *Genetic association study of QT interval highlights role for calcium signaling pathways in myocardial repolarization.* Nat Genet. **46**(8): p. 826-36.
21. Holm H, Gudbjartsson DF, Arnar DO, Thorleifsson G, Thorgeirsson G, Stefansdottir H, Gudjonsson SA, Jonasdottir A, Mathiesen EB, Njølstad I, Nyrnes A, Wilsgaard T, Hald EM, Hveem K, Stoltenberg C, Løchen ML, Kong A, Thorsteinsdottir U, Stefansson K. 2010. *Several common variants modulate heart rate, PR interval and QRS duration.* Nat Genet. **42**(2): p. 117-22.
22. Weidinger S, Gieger C, Rodriguez E, Baurecht H, Mempel M, Klopp N, Gohlke H, Wagenpfeil S, Ollert M, Ring J, Behrendt H, Heinrich J, Novak N, Bieber T, Krämer U, Berdel D, von Berg A, Bauer CP, Herbarth O, Koletzko S, Prokisch H, Mehta D, Meitinger T, Depner M, von Mutius E, Liang L, Moffatt M, Cookson W, Kabesch M, Wichmann HE, Illig T. 2008. *Genome-wide scan on total serum IgE levels identifies FCER1A as novel susceptibility locus.* PLoS Genet. **4**(8): p. e1000166.
23. Bønnelykke K, Sleiman P, Nielsen K, Kreiner-Møller E, Mercader JM, Belgrave D, den Dekker HT, Husby A, Sevelsted A, Faura-Tellez G, Mortensen LJ, Paternoster L, Flaaten R, Mølgaard A, Smart DE, Thomsen PF, Rasmussen MA, Bonàs-Guarch S, Holst C, Nohr EA, Yadav R, March ME, Blicher T, Lackie PM, Jaddoe VW, Simpson A, Holloway JW, Duijts L, Custovic A, Davies DE, Torrents D, Gupta R, Hollegaard MV, Hougaard DM, Hakonarson H, Bisgaard H.

2014. *A genome-wide association study identifies CDHR3 as a susceptibility locus for early childhood asthma with severe exacerbations.* Nat Genet. **46**(1): p. 51-5.

24. Li X, Howard TD, Zheng SL, Haselkorn T, Peters SP, Meyers DA, Bleecker ER. 2010. *Genome-wide association study of asthma identifies RAD50-IL13 and HLA-DR/DQ regions.* J Allergy Clin Immunol. **125**(2): p. 328-335.e11.

25. Weidinger S, Willis-Owen SA, Kamatani Y, Baurecht H, Morar N, Liang L, Edser P, Street T, Rodriguez E, O'Regan GM, Beattie P, Fölster-Holst R, Franke A, Novak N, Fahy CM, Winge MC, Kabesch M, Illig T, Heath S, Söderhäll C, Melén E, Pershagen G, Kere J, Bradley M, Lieden A, Nordenskjold M, Harper JI, McLean WH, Brown SJ, Cookson WO, Lathrop GM, Irvine AD, Moffatt MF. 2013. *A genome-wide association study of atopic dermatitis identifies loci with overlapping effects on asthma and psoriasis.* Hum Mol Genet. **22**(23): p. 4841-56.

26. Dichgans M, Malik R, König IR, Rosand J, Clarke R, Gretarsdottir S, Thorleifsson G, Mitchell BD, Assimes TL, Levi C, O'Donnell CJ, Fornage M, Thorsteinsdottir U, Psaty BM, Hengstenberg C, Seshadri S, Erdmann J, Bis JC, Peters A, Boncoraglio GB, März W, Meschia JF, Kathiresan S, Ikram MA, McPherson R, Stefansson K, Sudlow C, Reilly MP, Thompson JR, Sharma P, Hopewell JC, Chambers JC, Watkins H, Rothwell PM, Roberts R, Markus HS, Samani NJ, Farrall M, Schunkert H. 2014. *Shared genetic susceptibility to ischemic stroke and coronary artery disease: a genome-wide analysis of common variants.* Stroke. **45**(1): p. 24-36.

27. N'Diaye A, Chen GK, Palmer CD, Ge B, Tayo B, Mathias RA, Ding J, Nalls MA, Adeyemo A, Adoue V, Ambrosone CB, Atwood L, Bandera EV, Becker LC, Berndt SI, Bernstein L, Blot WJ, Boerwinkle E, Britton A, Casey G, Chanock SJ, Demerath E, Deming SL, Diver WR, Fox C, Harris TB, Hernandez DG, Hu JJ, Ingles SA, John EM, Johnson C, Keating B, Kittles RA, Kolonel LN, Kritchevsky SB, Le Marchand L, Lohman K, Liu J, Millikan RC, Murphy A, Musani S, Neslund-Dudas C, North KE, Nyante S, Ogunniyi A, Ostrander EA, Papanicolaou G, Patel S, Pettaway CA, Press MF, Redline S, Rodriguez-Gil JL, Rotimi C, Rybicki BA, Salako B, Schreiner PJ, Signorello LB, Singleton AB, Stanford JL, Stram AH, Stram DO, Strom SS, Suktitipat B, Thun MJ, Witte JS, Yanek LR, Ziegler RG, Zheng W, Zhu X, Zmuda JM, Zonderman AB, Evans MK, Liu Y, Becker DM, Cooper RS, Pastinen T, Henderson BE, Hirschhorn JN, Lettre G, Haiman CA. 2011. *Identification, replication, and fine-mapping of Loci associated with adult height in individuals of african ancestry.* PLoS Genet. **7**(10): p. e1002298.

28. Petrovski S, Fellay J, Shianna KV, Carpenetti N, Kumwenda J, Kamanga G, Kamwendo DD, Letvin NL, McMichael AJ, Haynes BF, Cohen MS, Goldstein DB. 2011. *Common human genetic variants and HIV-1 susceptibility: a genome-wide survey in a homogeneous African population.* Aids. **25**(4): p. 513-8.

29. Stein JL, Hua X, Morra JH, Lee S, Hibar DP, Ho AJ, Leow AD, Toga AW, Sul JH, Kang HM, Eskin E, Saykin AJ, Shen L, Foroud T, Pankratz N, Huentelman MJ, Craig DW, Gerber JD, Allen AN, Corneveaux JJ, Stephan DA, Webster J, DeChairo BM, Potkin SG, Jack CR, Jr., Weiner MW, Thompson PM. 2010. *Genome-wide analysis reveals novel genes influencing temporal lobe structure with relevance to neurodegeneration in Alzheimer's disease.* Neuroimage. **51**(2): p. 542-54.

30. O'Donnell CJ, Cupples LA, D'Agostino RB, Fox CS, Hoffmann U, Hwang SJ, Ingellson E, Liu C, Murabito JM, Polak JF, Wolf PA, Demissie S. 2007. *Genome-wide association study for subclinical atherosclerosis in major arterial territories in the NHLBI's Framingham Heart Study.* BMC Med Genet. **8 Suppl 1**(Suppl 1): p. S4.

31. Kapoor M, Wang JC, Wetherill L, Le N, Bertelsen S, Hinrichs AL, Budde J, Agrawal A, Almasy L, Bucholz K, Dick DM, Harari O, Xiaoling X, Hesselbrock V, Kramer J, Nurnberger JI, Jr., Rice J, Schuckit M, Tischfield J, Porjesz B, Edenberg HJ, Bierut L, Foroud T, Goate A. 2014. *Genome-wide survival analysis of age at onset of alcohol dependence in extended high-risk COGA families.* Drug Alcohol Depend. **142**: p. 56-62.

32. Awata T, Yamashita H, Kurihara S, Morita-Ohkubo T, Miyashita Y, Katayama S, Mori K, Yoneya S, Kohda M, Okazaki Y, Maruyama T, Shimada A, Yasuda K, Nishida N, Tokunaga K, Koike A. 2014. *A genome-wide association study for diabetic retinopathy in a Japanese population: potential association with a long intergenic non-coding RNA.* PLoS One. **9**(11): p. e111715.

33. Goldstein JI, Jarskog LF, Hilliard C, Alfirevic A, Duncan L, Fourches D, Huang H, Lek M, Neale BM, Ripke S, Shianna K, Szatkiewicz JP, Tropsha A, van den Oord EJ, Cascorbi I, Dettling M, Gazit E, Goff DC, Holden AL, Kelly DL, Malhotra AK, Nielsen J, Pirmohamed M, Rujescu D, Werge T, Levy DL, Josiassen RC, Kennedy JL, Lieberman JA, Daly MJ, Sullivan PF. 2014. *Clozapine-induced agranulocytosis is associated with rare HLA-DQB1 and HLA-B alleles.* Nat Commun. **5**: p. 4757.

34. Teumer A, Holtfreter B, Völker U, Petersmann A, Nauck M, Biffar R, Völzke H, Kroemer HK, Meisel P, Homuth G, Kocher T. 2013. *Genome-wide association study of chronic periodontitis in a general German population.* J Clin Periodontol. **40**(11): p. 977-85.
35. Chung SA, Brown EE, Williams AH, Ramos PS, Berthier CC, Bhangale T, Alarcon-Riquelme ME, Behrens TW, Criswell LA, Graham DC, Demirci FY, Edberg JC, Gaffney PM, Harley JB, Jacob CO, Kamboh MI, Kelly JA, Manzi S, Moser-Sivils KL, Russell LP, Petri M, Tsao BP, Vyse TJ, Zidovetzki R, Kretzler M, Kimberly RP, Freedman BI, Graham RR, Langefeld CD. 2014. *Lupus nephritis susceptibility loci in women with systemic lupus erythematosus.* J Am Soc Nephrol. **25**(12): p. 2859-70.
36. Williams FM, Carter AM, Hysi PG, Surdulescu G, Hodgkiss D, Soranzo N, Traylor M, Bevan S, Dichgans M, Rothwell PM, Sudlow C, Farrall M, Silander K, Kaunisto M, Wagner P, Saarela O, Kuulasmaa K, Virtamo J, Salomaa V, Amouyel P, Arveiler D, Ferrieres J, Wiklund PG, Ikram MA, Hofman A, Boncoraglio GB, Parati EA, Helgadottir A, Gretarsdottir S, Thorsteinsdottir U, Thorleifsson G, Stefansson K, Seshadri S, DeStefano A, Gschwendtner A, Psaty B, Longstreth W, Mitchell BD, Cheng YC, Clarke R, Ferrario M, Bis JC, Levi C, Attia J, Holliday EG, Scott RJ, Fornage M, Sharma P, Furie KL, Rosand J, Nalls M, Meschia J, Mosely TH, Evans A, Palotie A, Markus HS, Grant PJ, Spector TD. 2013. *Ischemic stroke is associated with the ABO locus: the EuroCLOT study.* Ann Neurol. **73**(1): p. 16-31.
37. He J, Kelly TN, Zhao Q, Li H, Huang J, Wang L, Jaquish CE, Sung YJ, Shimmin LC, Lu F, Mu J, Hu D, Ji X, Shen C, Guo D, Ma J, Wang R, Shen J, Li S, Chen J, Mei H, Chen CS, Chen S, Chen J, Li J, Cao J, Lu X, Wu X, Rice TK, Gu CC, Schwander K, Hamm LL, Liu D, Rao DC, Hixson JE, Gu D. 2013. *Genome-wide association study identifies 8 novel loci associated with blood pressure responses to interventions in Han Chinese.* Circ Cardiovasc Genet. **6**(6): p. 598-607.
38. Hinds DA, McMahon G, Kiefer AK, Do CB, Eriksson N, Evans DM, St Pourcain B, Ring SM, Mountain JL, Francke U, Davey-Smith G, Timpson NJ, Tung JY. 2013. *A genome-wide association meta-analysis of self-reported allergy identifies shared and allergy-specific susceptibility loci.* Nat Genet. **45**(8): p. 907-11.
39. Estrada K, Styrkarsdottir U, Evangelou E, Hsu YH, Duncan EL, Ntzani EE, Oei L, Albagha OM, Amin N, Kemp JP, Koller DL, Li G, Liu CT, Minster RL, Moayyeri A, Vandenput L, Willner D, Xiao SM, Yerges-Armstrong LM, Zheng HF, Alonso N, Eriksson J, Kammerer CM, Kaptoge SK, Leo PJ, Thorleifsson G, Wilson SG, Wilson JF, Aalto V, Alen M, Aragaki AK, Aspelund T, Center JR, Dailiana Z, Duggan DJ, Garcia M, Garcia-Giralt N, Giroux S, Hallmans G, Hocking LJ, Husted LB, Jameson KA, Khusainova R, Kim GS, Kooperberg C, Koromila T, Kruk M, Laaksonen M, Lacroix AZ, Lee SH, Leung PC, Lewis JR, Masi L, Mencej-Bedrac S, Nguyen TV, Nogues X, Patel MS, Prezelj J, Rose LM, Scollen S, Siggeirsdottir K, Smith AV, Svensson O, Trompet S, Trummer O, van Schoor NM, Woo J, Zhu K, Balcells S, Brandi ML, Buckley BM, Cheng S, Christiansen C, Cooper C, Dedoussis G, Ford I, Frost M, Goltzman D, González-Macías J, Kähönen M, Karlsson M, Khusnutdinova E, Koh JM, Kollia P, Langdahl BL, Leslie WD, Lips P, Ljunggren Ö, Lorenc RS, Marc J, Mellström D, Obermayer-Pietsch B, Olmos JM, Pettersson-Kymmer U, Reid DM, Riancho JA, Ridker PM, Rousseau F, Slagboom PE, Tang NL, Urreizti R, Van Hul W, Viikari J, Zarrabeitia MT, Aulchenko YS, Castano-Betancourt M, Grundberg E, Herrera L, Ingvarsson T, Johannsdottir H, Kwan T, Li R, Luben R, Medina-Gómez C, Palsson ST, Reppe S, Rotter JI, Sigurdsson G, van Meurs JB, Verlaan D, Williams FM, Wood AR, Zhou Y, Gautvik KM, Pastinen T, Raychaudhuri S, Cauley JA, Chasman DI, Clark GR, Cummings SR, Danoy P, Dennison EM, Eastell R, Eisman JA, Gudnason V, Hofman A, Jackson RD, Jones G, Jukema JW, Khaw KT, Lehtimäki T, Liu Y, Lorentzon M, McCloskey E, Mitchell BD, Nandakumar K, Nicholson GC, Oostra BA, Peacock M, Pols HA, Prince RL, Raitakari O, Reid IR, Robbins J, Sambrook PN, Sham PC, Shuldiner AR, Tylavsky FA, van Duijn CM, Wareham NJ, Cupples LA, Econs MJ, Evans DM, Harris TB, Kung AW, Psaty BM, Reeve J, Spector TD, Streeten EA, Zillikens MC, Thorsteinsdottir U, Ohlsson C, Karasik D, Richards JB, Brown MA, Stefansson K, Uitterlinden AG, Ralston SH, Ioannidis JP, Kiel DPRivadeneira F. 2012. *Genome-wide meta-analysis identifies 56 bone mineral density loci and reveals 14 loci associated with risk of fracture.* Nat Genet. **44**(5): p. 491-501.
40. Lambert JC, Ibrahim-Verbaas CA, Harold D, Naj AC, Sims R, Bellenguez C, DeStafano AL, Bis JC, Beecham GW, Grenier-Boley B, Russo G, Thorton-Wells TA, Jones N, Smith AV, Chouraki V, Thomas C, Ikram MA, Zelenika D, Vardarajan BN, Kamatani Y, Lin CF, Gerrish A, Schmidt H, Kunkle B, Dunstan ML, Ruiz A, Bihoreau MT, Choi SH, Reitz C, Pasquier F, Cruchaga C, Craig D, Amin N, Berr C, Lopez OL, De Jager PL, Deramecourt V, Johnston JA, Evans D, Lovestone S, Letenneur L, Morón FJ, Rubinsztein DC, Eiriksdottir G, Sleegers K, Goate AM, Fiévet N, Huentelman MW, Gill M, Brown K, Kamboh MI, Keller L, Barberger-Gateau P, McGuiness B, Larson EB, Green R, Myers AJ, Dufouil C, Todd S, Wallon D, Love S, Rogaeva E, Gallacher J, St George-Hyslop P, Clarimon J, Lleo A, Bayer A, Tsuang DW, Yu L, Tsolaki M, Bossù P, Spalletta G, Proitsi P, Collinge J, Sorbi S, Sanchez-Garcia F, Fox NC, Hardy J, Deniz Naranjo MC, Bosco P,

Clarke R, Brayne C, Galimberti D, Mancuso M, Matthews F, Moebus S, Mecocci P, Del Zompo M, Maier W, Hampel H, Pilotto A, Bullido M, Panza F, Caffarra P, Nacmias B, Gilbert JR, Mayhaus M, Lannefelt L, Hakonarson H, Pichler S, Carrasquillo MM, Ingelsson M, Beekly D, Alvarez V, Zou F, Valladares O, Younkin SG, Coto E, Hamilton-Nelson KL, Gu W, Razquin C, Pastor P, Mateo I, Owen MJ, Faber KM, Jonsson PV, Combarros O, O'Donovan MC, Cantwell LB, Soininen H, Blacker D, Mead S, Mosley TH, Jr., Bennett DA, Harris TB, Fratiglioni L, Holmes C, de Bruijn RF, Passmore P, Montine TJ, Bettens K, Rotter JI, Brice A, Morgan K, Foroud TM, Kukull WA, Hannequin D, Powell JF, Nalls MA, Ritchie K, Lunetta KL, Kauwe JS, Boerwinkle E, Riemenschneider M, Boada M, Hiltuenen M, Martin ER, Schmidt R, Rujescu D, Wang LS, Dartigues JF, Mayeux R, Tzourio C, Hofman A, Nöthen MM, Graff C, Psaty BM, Jones L, Haines JL, Holmans PA, Lathrop M, Pericak-Vance MA, Launer LJ, Farrer LA, van Duijn CM, Van Broeckhoven C, Moskvina V, Seshadri S, Williams J, Schellenberg GDAmouyel P. 2013. *Meta-analysis of 74,046 individuals identifies 11 new susceptibility loci for Alzheimer's disease.* Nat Genet. **45**(12): p. 1452-8.

41. Pyun JA, Kim S, Cho NH, Koh I, Lee JY, Shin C, Kwack K. 2014. *Genome-wide association studies and epistasis analyses of candidate genes related to age at menarche and age at natural menopause in a Korean population.* Menopause. **21**(5): p. 522-9.

42. Schumacher FR, Berndt SI, Siddiq A, Jacobs KB, Wang Z, Lindstrom S, Stevens VL, Chen C, Mondul AM, Travis RC, Stram DO, Eeles RA, Easton DF, Giles G, Hopper JL, Neal DE, Hamdy FC, Donovan JL, Muir K, Al Olama AA, Kote-Jarai Z, Guy M, Severi G, Grönberg H, Isaacs WB, Karlsson R, Wiklund F, Xu J, Allen NE, Andriole GL, Barricarte A, Boeing H, Bueno-de-Mesquita HB, Crawford ED, Diver WR, Gonzalez CA, Gaziano JM, Giovannucci EL, Johansson M, Le Marchand L, Ma J, Sieri S, Stattin P, Stampfer MJ, Tjonneland A, Vineis P, Virtamo J, Vogel U, Weinstein SJ, Yeager M, Thun MJ, Kolonel LN, Henderson BE, Albanes D, Hayes RB, Feigelson HS, Riboli E, Hunter DJ, Chanock SJ, Haiman CA, Kraft P. 2011. *Genome-wide association study identifies new prostate cancer susceptibility loci.* Hum Mol Genet. **20**(19): p. 3867-75.

43. 2011. *Genome-wide association study identifies five new schizophrenia loci.* Nat Genet. **43**(10): p. 969-76.

44. Grassi MA, Tikhomirov A, Ramalingam S, Below JE, Cox NJ, Nicolae DL. 2011. *Genome-wide meta-analysis for severe diabetic retinopathy.* Hum Mol Genet. **20**(12): p. 2472-81.

45. 2013. *Identification of risk loci with shared effects on five major psychiatric disorders: a genome-wide analysis.* Lancet. **381**(9875): p. 1371-1379.

46. Potkin SG, Turner JA, Guffanti G, Lakatos A, Fallon JH, Nguyen DD, Mathalon D, Ford J, Lauriello J, Macciardi F. 2009. *A genome-wide association study of schizophrenia using brain activation as a quantitative phenotype.* Schizophr Bull. **35**(1): p. 96-108.

47. Shoubridge C, Tarpey PS, Abidi F, Ramsden SL, Rujirabanjerd S, Murphy JA, Boyle J, Shaw M, Gardner A, Proos A, Puusepp H, Raymond FL, Schwartz CE, Stevenson RE, Turner G, Field M, Walikonis RS, Harvey RJ, Hackett A, Futreal PA, Stratton MR, Gécz J. 2010. *Mutations in the guanine nucleotide exchange factor gene IQSEC2 cause nonsyndromic intellectual disability.* Nat Genet. **42**(6): p. 486-8.
