# Supplementary material for: APEX2 Proximity Proteomics Resolves Flagellum Subdomains and Identifies Flagellum Tip-Specific Proteins in Trypanosoma brucei
Source: mSphere. 2021 Feb 10;6(1):e01090-20. doi: 10.1128/mSphere.01090-20 (PMC8141408; doi:10.1128/mSphere.01090-20)
Supplement: TABLE S3 [file mSphere.01090-20-st003.pdf]

Supplemental Table S3. Proteins used for Figure 4.

Tripartite Attachment Complex	Basal Body	Transition Zone	Central Pair	Radial Spokes	Microtubule Inner Proteins	Ruler	Dynein Regulatory Complex	Docking Complex	Dynein Heavy Chain	Intraflagellar Transport	Flagellar Connector
Tb927.4.1610[1]	Tb927.1.3560[6]	Tb927.3.1420[10]	Tb927.1.2670[11]	Tb927.3.2890[12]	Tb927.3.1060[13]	Tb927.8.8200[15]	Tb927.5.500[16]	Tb927.8.4400[20]	Tb927.2.5270[21]	Tb927.3.1110[22]	Tb927.1.2710[26]
Tb927.5.830[2]	Tb927.5.2440[6]	Tb927.4.2400[10]	Tb927.10.13960[11]	Tb927.3.3690[12]	Tb927.3.2310[14]	Tb927.10.11250[15]	Tb927.5.2270[16]	Tb927.5.1900[20]	Tb927.3.930[21]	Tb927.3.3000[22]	Tb927.3.4960[26]
Tb927.7.2390[3]	Tb927.7.3410[7]	Tb927.5.4470[10]		Tb927.7.6910[12]	Tb927.5.2950[13]		Tb927.6.5030[16]	Tb927.7.5660[20]	Tb927.4.560[21]	Tb927.3.5490[23]	Tb927.8.940[26]
Tb927.7.5700[2]	Tb927.7.6250[6]	Tb927.7.750[10]		Tb927.8.810[12]	Tb927.8.4640[13]		Tb927.8.5020[16]	Tb927.11.16090[20]	Tb927.4.870[21]	Tb927.3.5550[22]	Tb927.8.7540[26]
Tb927.10.15750[4]	Tb927.7.6840[6]	Tb927.9.4640[10]		Tb927.11.1150[12]	Tb927.9.9940[14]		Tb927.9.15050[17]		Tb927.7.920[21]	Tb927.6.3100[24]	Tb927.10.890[26]
Tb927.11.3290[5]	Tb927.8.1080[7]	Tb927.9.8490[10]		Tb927.11.13020[12]	Tb927.10.7690[13]		Tb927.10.6350[16]		Tb927.8.3250[21]	Tb927.6.3290[23]	Tb927.11.1340[26]
	Tb927.8.6070[8,9]	Tb927.10.7000[10]		Tb927.11.13690[12]	Tb927.11.1430[13]		Tb927.10.7880[16]		Tb927.10.5350[21]	Tb927.7.3370[22]	Tb927.11.8150[26]
	Tb927.10.2860[6]	Tb927.10.11840[10]		Tb927.11.18680[12]			Tb927.11.3360[18]		Tb927.11.2430[21]	Tb927.10.450[22]	Tb927.11.4470[26]
	Tb927.10.5990[6]	Tb927.10.12130[10]					Tb927.11.7240[19]		Tb927.11.3250[21]	Tb927.10.1170[23]	
	Tb927.10.7600[6]	Tb927.11.380[10]					Tb927.11.7940[16]		Tb927.11.8160[21]	Tb927.10.2640[22]	
	Tb927.10.10690[6]	Tb927.11.5750[10]					Tb927.11.10430[16]		Tb927.11.11220[21]	Tb927.10.11310[23]	
	Tb927.10.14110[6]	Tb927.11.16640[10]								Tb927.10.14980[23]	
	Tb927.11.6560[6]									Tb927.10.14990[23]	
	Tb927.11.8860[6]									Tb927.11.1740[23]	
	Tb927.11.8920[6]									Tb927.11.16830[25]	
	Tb927.11.11650[6]										
	Tb927.11.13700[6]										

1. Schnarwiler F, Niemann M, Doiron N, Harsman A, Kaser S, Mani J, Chanfon A, Dewar CE, Oeljeklaus S, Jackson CB, Pusnik M, Schmidt O, Meisinger C, Hiller S, Warscheid B, Schnaufer AC, Ochsenreiter T, Schneider A. 2014. *Trypanosomal TAC40 constitutes a novel subclass of mitochondrial beta-barrel proteins specialized in mitochondrial genome inheritance.* Proc Natl Acad Sci U S A. **111**(21): p. 7624-9.
2. Kaser S, Oeljeklaus S, Tyc J, Vaughan S, Warscheid B, Schneider A. 2016. *Outer membrane protein functions as integrator of protein import and DNA inheritance in mitochondria.* Proc Natl Acad Sci U S A. **113**(31): p. E4467-75.
3. Trikin R, Doiron N, Hoffmann A, Haenni B, Jakob M, Schnaufer A, Schimanski B, Zuber B, Ochsenreiter T. 2016. *TAC102 Is a Novel Component of the Mitochondrial Genome Segregation Machinery in Trypanosomes.* PLoS Pathog. **12**(5): p. e1005586.
4. Gheiratmand L, Brasseur A, Zhou Q, He CY. 2013. *Biochemical characterization of the bi-lobe reveals a continuous structural network linking the bi-lobe to other single-copied organelles in Trypanosoma brucei.* J Biol Chem. **288**(5): p. 3489-99.
5. Zhao Z, Lindsay ME, Roy Chowdhury A, Robinson DR, Englund PT. 2008. *p166, a link between the trypanosome mitochondrial DNA and flagellum, mediates genome segregation.* Embo j. **27**(1): p. 143-54.
6. Dang HQ, Zhou Q, Rowlett VW, Hu H, Lee KJ, Margolin W, Li Z. 2017. *Proximity Interactions among Basal Body Components in Trypanosoma brucei Identify Novel Regulators of Basal Body Biogenesis and Inheritance.* MBio. **8**(1).
7. He CY, Pypaert M, Warren G. 2005. *Golgi duplication in Trypanosoma brucei requires Centrin2.* Science. **310**(5751): p. 1196-8.
8. Dilbeck V, Berberof M, Van Cauwenberge A, Alexandre H, Pays E. 1999. *Characterization of a coiled coil protein present in the basal body of Trypanosoma brucei.* J Cell Sci. **112 (Pt 24)**: p. 4687-94.
9. Absalon S, Kohl L, Branche C, Blisnick T, Toutirais G, Rusconi F, Cosson J, Bonhivers M, Robinson D, Bastin P. 2007. *Basal body positioning is controlled by flagellum formation in Trypanosoma brucei.* PLoS One. **2**(5): p. e437.
10. Dean S, Moreira-Leite F, Varga V, Gull K. 2016. *Cilium transition zone proteome reveals compartmentalization and differential dynamics of ciliopathy complexes.* Proc Natl Acad Sci U S A. **113**(35): p. E5135-43.
11. Ralston KS, Lerner AG, Diener DR, Hill KL. 2006. *Flagellar motility contributes to cytokinesis in Trypanosoma brucei and is modulated by an evolutionarily conserved dynein regulatory system.* Eukaryot Cell. **5**(4): p. 696-711.
12. Yang C, Compton MM, Yang P. 2005. *Dimeric novel HSP40 is incorporated into the radial spoke complex during the assembly process in flagella.* Mol Biol Cell. **16**(2): p. 637-48.
13. Baron DM, Ralston KS, Kabututu ZP, Hill KL. 2007. *Functional genomics in Trypanosoma brucei identifies evolutionarily conserved components of motile flagella.* J Cell Sci. **120**(Pt 3): p. 478-91.
14. Dawe HR, Farr H, Portman N, Shaw MK, Gull K. 2005. *The Parkin co-regulated gene product, PACRG, is an evolutionarily conserved axonemal protein that functions in outer-doublet microtubule morphogenesis.* J Cell Sci. **118**(Pt 23): p. 5421-30.
15. Oda T, Yanagisawa H, Kamiya R, Kikkawa M. 2014. *A molecular ruler determines the repeat length in eukaryotic cilia and flagella.* Science. **346**(6211): p. 857-60.
16. Hill KL. unpublished.
17. Ralston KSHill KL. 2006. *Trypanin, a component of the flagellar Dynein regulatory complex, is essential in bloodstream form African trypanosomes.* PLoS Pathog. **2**(9): p. e101.
18. Nguyen HT, Sandhu J, Langousis G, Hill KL. 2013. *CMF22 is a broadly conserved axonemal protein and is required for propulsive motility in Trypanosoma brucei.* Eukaryot Cell. **12**(9): p. 1202-13.
19. Kabututu ZP, Thayer M, Melehani JH, Hill KL. 2010. *CMF70 is a subunit of the dynein regulatory complex.* J Cell Sci. **123**(Pt 20): p. 3587-95.
20. Edwards BFL, Wheeler RJ, Barker AR, Moreira-Leite FF, Gull K, Sunter JD. 2018. *Direction of flagellum beat propagation is controlled by proximal/distal outer dynein arm asymmetry.* Proc Natl Acad Sci U S A. **115**(31): p. E7341-e7350.
21. Ralston KS, Kabututu ZP, Melehani JH, Oberholzer M, Hill KL. 2009. *The Trypanosoma brucei flagellum: moving parasites in new directions.* Annu Rev Microbiol. **63**: p. 335-62.

22. Subota I, Julkowska D, Vincensini L, Reeg N, Buisson J, Blisnick T, Huet D, Perrot S, Santi-Rocca J, Duchateau M, Hourdel V, Rousselle JC, Cayet N, Namane A, Chamot-Rooke J, Bastin P. 2014. *Proteomic analysis of intact flagella of procyclic Trypanosoma brucei cells identifies novel flagellar proteins with unique sub-localization and dynamics.* Mol Cell Proteomics. **13**(7): p. 1769-86.
23. Absalon S, Blisnick T, Kohl L, Toutirais G, Dore G, Julkowska D, Tavenet A, Bastin P. 2008. *Intraflagellar transport and functional analysis of genes required for flagellum formation in trypanosomes.* Mol Biol Cell. **19**(3): p. 929-44.
24. Franklin JBUllu E. 2010. *Biochemical analysis of PIFTC3, the Trypanosoma brucei orthologue of nematode DYF-13, reveals interactions with established and putative intraflagellar transport components.* Mol Microbiol. **78**(1): p. 173-86.
25. Huet D, Blisnick T, Perrot S, Bastin P. 2014. *The GTPase IFT27 is involved in both anterograde and retrograde intraflagellar transport.* Elife. **3**: p. e02419.
26. Varga V, Moreira-Leite F, Portman N, Gull K. 2017. *Protein diversity in discrete structures at the distal tip of the trypanosome flagellum.* Proc Natl Acad Sci U S A. **114**(32): p. E6546-e6555.
